# The Use of Artificial Intelligence (AI) to Support Dietetic Practice Across Primary Care: A Scoping Review of the Literature

**DOI:** 10.3390/nu17223515

**Published:** 2025-11-10

**Authors:** Kaitlyn Ngo, Simone Mekhail, Virginia Chan, Xinyi Li, Annabelle Yin, Ha Young Choi, Margaret Allman-Farinelli, Juliana Chen

**Affiliations:** 1 Nutrition and Dietetics Discipline, Susan Wakil School of Nursing and Midwifery, Faculty of Medicine and Health, The University of Sydney, Sydney, NSW 2006, Australia; kaitlyn.ngoapd@gmail.com (K.N.); margaret.allman-farinelli@sydney.edu.au (M.A.-F.); 2 Charles Perkins Centre, The University of Sydney, Sydney, NSW 2006, Australia

**Keywords:** nutrition care process (NCP), medical nutrition therapy (MNT), primary care, healthcare, dietetics, artificial intelligence (AI)

## Abstract

**Background/objectives**: The nutrition care process (NCP) is an evidence-based practice framework used in Medical Nutrition Therapy for the prevention, treatment, and management of non-communicable chronic health conditions. This review aimed to explore available artificial intelligence (AI)-integrated technologies across the NCP in dietetic primary care, their uses, and their impacts on the NCP and patient outcomes. **Method:** Six databases were searched: MEDLINE, Embase, PsycINFO, Scopus, IEEE, and ACM digital library. Eligible studies were published between January 2007 and August 2024 and included human adult studies, AI-integrated technologies in the dietetic primary care setting, and patient-related outcomes. Extracted details focused on participant characteristics, dietitian involvement, and the type of AI system and its application in the NCP. **Results:** Ninety-seven studies were included. Three different AI systems (image or audio recognition, chatbots, and recommendation systems) were found. These were implemented in web-based or smartphone applications, wearable sensor systems, smart utensils, and software. Most AI-integrated technologies could be incorporated into one or more NCP stages. Seventy-nine studies reported user- or patient-related outcomes, with mixed findings, but all highlighted efficiencies of using AI. Higher patient engagement was observed with Chatbots. Seventeen studies raised concerns encompassing ethics and patient safety. **Conclusions:** AI systems show promise as a clinical support tool across most stages of the NCP. Whilst they have varying degrees of accuracy, AI demonstrates potential in improving efficiency, supporting personalised nutrition, and enhancing chronic disease management outcomes. Integrating AI education into dietetic training and professional development will be essential to ensure safe and effective use in practice.

## 1. Introduction

**Figure 1:**
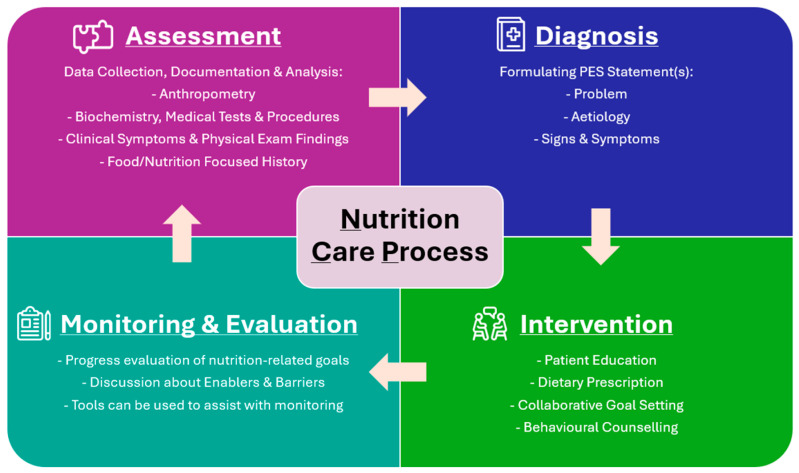
The nutrition care process model (adapted from the Academy of Nutrition and Dietetics) [4].

Non-communicable diseases (NCDs) currently account for approximately 74% of global deaths [1]. Poor diet and metabolic factors substantially elevate the risk of NCDs, including cardiovascular disease, colorectal cancer, and obesity-related conditions [1]. Modern food systems contribute to poorer diets characterised by energy-dense and nutrient-poor ultra-processed foods (typically containing added sugars, salt and/or saturated fats, food additives, and potential contaminants from packaging material) [2,3]. Registered Dietitians or Accredited Practising Dietitians are the only qualified healthcare professionals recognised to provide Medical Nutrition Therapy (MNT) to prevent, treat, or manage NCDs. MNT applies evidence-based practice (EBP) by following the nutrition care process (NCP) framework [4,5]. This is a standardised clinical framework that entails: 1. Nutrition Assessment (and reassessment) (including anthropometric, biochemical, clinical data collection, and interpretation), 2. Nutrition Diagnosis, 3. Nutrition Intervention, and 4. Monitoring and Evaluation (Figure 1) [4]. The NCP model is adaptable to patient progress, enabling dietitians to revisit prior stages as new clinical information emerges to support timely prevention or management of chronic conditions, particularly in primary healthcare [4,6]. Effectiveness of MNT in primary care is contingent on the dietitians’ ability to deliver tailored nutritional guidance and the patient’s active engagement in implementing and adhering to clinical recommendations.

Primary care is the first point of contact with healthcare services for individuals within the community for the prevention, treatment, and management of health conditions. Common primary care providers include family medicine or general practitioners, nurses, allied health professionals, and pharmacies [7]. Primary care dietitians navigate an ongoing challenge to balancing workflow efficiency with clinical effectiveness to meet patient needs and expectations [8]. The rising prevalence of chronic health conditions strains the efficiency and reach of the traditional NCP provided by dietitians, thus underscoring the importance of new technological innovations in dietetics. The evolving landscape of artificial intelligence (AI) has become an area of growing interest in nutrition [9]. AI relies on machine learning algorithms to build systems capable of analysing large datasets to detect patterns or relationships to inform predictions and outputs [9,10]. Through cycles of trial and error, AI systems can mimic human cognitive processes, including stimulus recognition, problem solving, and decision-making [9]. Leveraging machine learning and deep learning capabilities of AI could transform the ways in which dietitians engage with each stage of the NCP, from synthesising nutrition assessment data, tailoring interventions, and predicting patient nutrition-related outcomes [9,11].

Use of digital technologies and devices as a tool to assist in the NCP is not new to healthcare. Technologies such as wearable devices, sensors, interactive platforms, and mobile-based or mobile health (mHealth) applications (apps), alongside web-based tools, have become more commonplace and effective in supporting dietitians in clinical practice [11,12,13]. To date, the most notable contributions of AI embedded technology include assisting with detailed data collection in NCD management (e.g., dietary patterns, nutrient breakdowns, health monitoring), as well as screening tools (e.g., refeeding syndrome risk) [9,11,12,14]. These innovations bring notable efficiencies, such as accurate nutritional diagnosis with more streamlined patient data; greater time allocations to delivering nutritional interventions; and the encouragement of active patient involvement to facilitate patient-driven care [14,15]. There will be projected growth in clinical efficiencies as AI models continue to evolve. 

While AI is a promising tool to supplement dietetic clinical practice, concerns surrounding ethical frameworks and privacy are controversial topics that need to be addressed [9,15]. These include biases of ingrained AI algorithms stemming from low-quality training datasets representative of certain genders, ethnicities, or socioeconomic groups. In turn, inappropriate recommendations or advice can be generated for underrepresented populations, which can ultimately be detrimental to patient health and safety [9]. Concerns over data privacy will continually need to be addressed through regulations and security measures built into AI-integrated technologies to ensure sensitive patient data remain protected [9].

To our knowledge, the prior literature has generally offered a broad picture of prospective AI-integrated technologies in nutrition. This review provides a refined focus on primary care—the fundamental setting for managing and treating NCDs through clinicians engaging with patients to guide behavioural changes. By synthesising the latest evidence, this scoping review aims to provide a comprehensive overview of how AI-integrated technologies can be integrated into dietetic practice to assist in NCP delivery and its impact on patient outcomes.

## 2. Materials and Methods

This review was registered with the Open Science Framework (https://osf.io/2dwyh, accessed on 5 August 2025). The scoping review process was conducted in accordance with the Joanna Briggs Institute Manual (Chapter 10) Scoping Reviews outline [16]. The review process and reporting were aligned with Preferred Reporting Items for Systematic and Meta-Analyses extension for Scoping Reviews (PRISMA-ScR) to ensure consistency and adherence to best practice [17].

### 2.1. Search Strategy

A preliminary pilot search of MEDLINE and IEEE was undertaken to identify articles relevant to the topic. Keywords contained in the titles, abstracts, and medical subject headings (MeSH) of articles were used to generate a search strategy which was adapted across six electronic databases: MEDLINE, Embase, PsycINFO, Scopus, IEEE, and ACM digital library. These databases were searched for peer-reviewed, original literature published from 1 January 2007 to 20 August 2024, a period during which research on the use of AI was rapidly evolving. Keywords and subject headings (including MeSH) were guided by the Population, Concept, and Context (PCC) Framework (Table 1). Key search terms included artificial intelligence, machine learning, deep learning, image recognition, generative AI, chatbots, medication nutrition therapy, nutrition care process (NCP), nutritionist/dietitian, private practice, and related synonyms. The final searches for each database are included in the Supplementary Materials (Tables S1–S5).

### 2.2. Source of Evidence Selection

All citations were exported into EndNote 21 citation management software (Clarivate Analytics, Philadelphia, PA, USA), and duplicate records were removed. Citations were then imported into Covidence 2024 software (Veritas Health Innovation, Melbourne, Australia) for screening.

**Figure 2:**
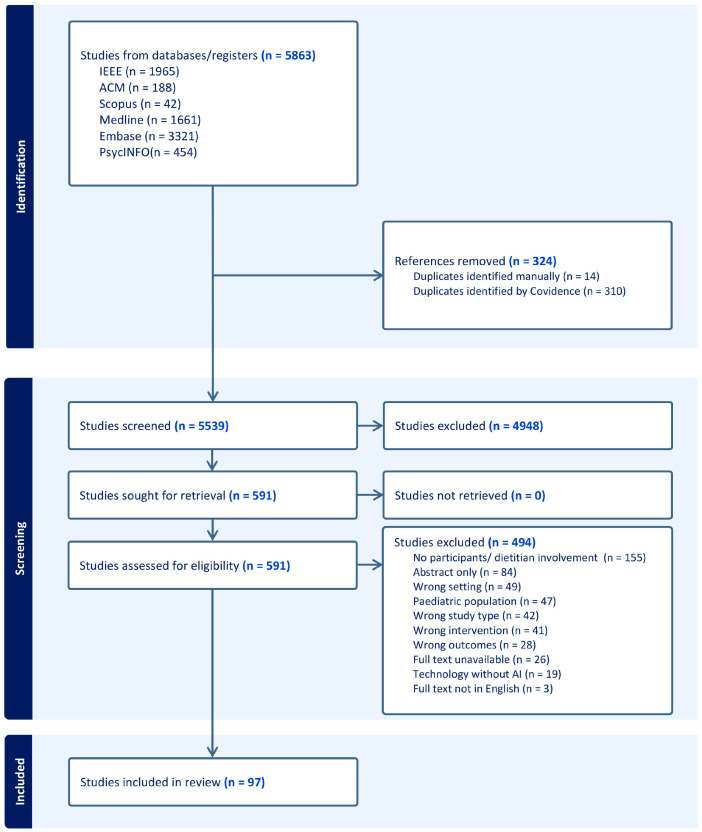
PRISMA flow diagram for scoping review study selection.

Titles and abstracts were dual screened separately by two independent reviewers (K.N. and S.M.) to minimise bias. The same two reviewers (K.N. and S.M.) conducted a full-text review to identify articles eligible for inclusion in the review. The same review criteria (Table 1) were consistently applied across all stages of screening. For any articles that were excluded, a reason was documented and reported in a PRISMA-ScR flow diagram (Figure 2). Discrepancies at each screening stage were discussed between the two reviewers (K.N. and S.M.), and an additional reviewer (J.C./V.C./M.A.F.) was consulted to reach a consensus.

### 2.3. Data Extraction

Data from relevant studies were extracted by two reviewers (K.N. and S.M.) to ensure accuracy and completeness. An Excel spreadsheet was utilised as the main data extraction and collation tool. Extraction of data was guided by areas of interest that would address the research aim as outlined in Table 2.

### 2.4. Data Analysis and Presentation

Main findings addressing the scoping review’s objectives were collated and presented in tabular form and diagrams, with an accompanying narrative synthesis in Section 3. 

## 3. Results

### 3.1. Findings from Search Strategy

The search retrieved 5863 records (Figure 2). After the removal of duplicates, 5539 studies underwent title–abstract screening, where a further 4948 studies were excluded. Full-text screening was conducted for 591 studies, in which only 97 studies were eligible.

### 3.2. Characteristics of Included Studies

From the 97 studies, 34 were descriptive studies, 7 were mixed methods studies, ad 56 were classified as aalytical studies (experimental, observational, or randomised controlled trials (RCTs)). In total, 11 were RCTs, and 6 were pilot studies. First authors had academic backgrounds in informatics or computer science (*n* = 23), various fields of engineering (*n* = 19), nutrition science and dietetics (*n* = 19), medicine (*n* = 18), biological and life sciences (*n* = 5), public health (*n* = 4), and economics (*n* = 1). The included papers were predominantly published from the following countries: United States of America (*n* = 23), Switzerland (*n* = 8), Taiwan (*n* = 6), Australia (*n* = 5), Germany (*n* = 5), Japan (*n* = 5) and Italy (*n* = 5). The remaining publications were from a variety of other countries (*n* = 40) (Supplementary Table S6)

Chronic medical conditions of focus included weight management (overweight or obesity) (*n* = 13), diabetes mellitus (type 1, type 2, and prediabetes) (*n* = 12), gastrointestinal (*n* = 7), renal (*n* = 3), cardiovascular (*n* = 1), metabolic Syndrome (*n* = 3), elderly nutrition (particularly malnutrition) (*n* = 3), two or more comorbid medical conditions (*n* = 2), and others (includes cancer, neurological, and food allergies) (*n* = 3). The remaining studies focused on healthy participants (*n* = 35) or did not specify a specific medical condition (*n* = 18) (Table 3). There were varying sample sizes (range: 1–123,787) and age ranges (range: 18–91 years old) amongst the participants in the included studies (Supplementary Table S6). Approximately two-thirds (68%) of the studies included dietitian, nutritionist, or nutrition expert involvement (*n* = 66), whilst the remainder did not (*n* = 31).

The AI-integrated technologies explored could be classified under three categories: image or audio recognition (*n* = 53), chatbots (*n* = 22), and recommendation systems (*n* = 50). Three-quarters (75%) of the AI-integrated technologies only utilised one form of AI system (*n* = 73). Whilst with other technologies, they utilised a combination of either two (*n* = 20) or all three forms (*n* = 4). These AI-integrated technologies were available via the web (*n* = 28), smartphone apps (*n* = 34), a combination of both web and smart phone apps (n = 5), wearable sensor systems (*n* = 21), smart utensils (*n* = 2), and system or database development (*n* = 7) or were not clearly specified by study (*n* = 3). When further deconstructed by subdomains and specific techniques of AI, digital technologies employed machine learning (*n* = 31), deep learning (*n* = 17), natural language processing (inclusive of fuzzy logic, language learning models, and generative pre-trained transformers) (*n* = 19), and genetic/evolutionary algorithms (*n* = 3). In some cases, digital technologies utilised more than one subset of AI (*n* = 10). There are some studies that did not explicitly specify the subset of AI embedded in their technology (*n* = 17) (Table 4).

Sixty-two studies assessed the performance of AI-integrated technologies using evaluation metrics. More notable assessment metrics included accuracy (*n* = 27), precision (*n* = 8), recall (*n* = 7), F1 scores (the harmonic means between precision and recall) (*n* = 6), and user experience (*n* = 6) (Table 4).

### 3.3. AI Involvement in the Stages of the Nutrition Care Process (NCP)

**Figure 3:**
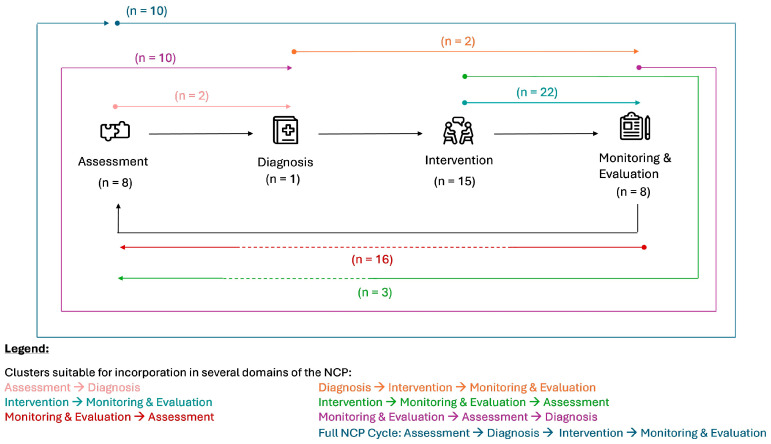
Distribution of included studies (*n* = 97) based on AI-integrated technology’s potential contribution to the stage(s) of the nutrition care process (NCP). The NCP is a standardised cyclical framework that entails the following: 1. nutrition assessment (and reassessment) (including anthropometric, biochemical, clinical data collection, and interpretation); 2. nutrition diagnosis; 3. nutrition intervention; 4. monitoring and evaluation [4]. Legend in the diagram outlines the clusters of different NCP stages that AI-integrated technologies can support. Bold lines indicate involvement at that NCP stage. Dotted lines indicate that a certain NCP stage is not involved within the cluster.

The results relating to the application of AI-integrated technologies in dietetic primary care can be further grouped in accordance with the four main elements of the NCP cycle: assessment, diagnosis, intervention, and monitoring and evaluation [4,5]. Across the 97 studies, the functions of AI-integrated technologies could be incorporated into at least one (*n* = 31), two (*n* = 41), three (*n* = 15), or all four (*n* = 10) stages of the NCP (Figure 3). Table 4 summarises the different AI-integrated technologies and their application in the NCP model’s stages. 

#### 3.3.1. Nutrition Assessment

Eight studies focused on the use of smartphone apps and wearable sensor technologies (e.g., glasses, in-ear microphones) to capture images or audio files and detect or identify participant food intake to aid automated diet histories [20,30,35,70,74,86,87,105]. Wearable sensors employed classifier algorithms to label instances and types of food consumed by participants. The common inputs for these algorithms included food images, audio files (e.g., chewing sounds), or electromyography (EMG: measure of electrical activity from nerve stimulation of muscles). For instance, a study by Papapanagiotou and colleagues (2021) explored the use of commercially available Samsung Galaxy earbuds to record audio to input into machine learning and deep learning algorithms, determine bite weight estimation, and record food intake occasions for participants [87]. A notable study by Papathanail and colleagues (2022) explored a deep learning convoluted neural network system which could take inputs from apps collecting diet history and calculate an adherence score with respect to the Mediterranean diet’s guidelines [90]. Similarly, AI-integrated technologies developed based on data mapping and machine learning models have been able to yield patient dietary intake profiles that are comparable to validated standards, such as ASA24 reports [34,79].

#### 3.3.2. Nutrition Diagnosis

One study focused on technology that could specifically support dietitians in reaching nutrition diagnoses in the NCP [32]. This study proposed and evaluated a web-based nutrition expert diagnosis system which provided recommendations to assist clinical dietitians in reaching a more informed diagnosis. The nutrition diagnosis expert system was programmed with a specific set of fifty different nutritional diagnosis rules. This system relied on a dietitian’s input of dietary assessment data to specifically produce a nutrition diagnosis report addressing protein energy malnutrition. The AI-integrated technology was more efficient and achieved 100% accuracy, outperforming a human dietitian who served as the standard of comparison. 

Another two studies [98,102] also proposed technologies that had the potential to assist with the diagnosis, intervention, and monitoring and evaluation stages. Recommendation systems and image recognition AI algorithms formed the basis of both innovations, using personalised dietary data analysis and patient diagnosis to develop meal recommendations to meet daily macronutrient needs [102] or support obesity management [98].

#### 3.3.3. Nutrition Intervention

Among the studies, four studies solely focused on assisting the provision of nutrition interventions employed chatbots [19,83,91,92], eight used recommender systems [26,31,53,57,62,76,88,113], and three employed a combination of both types of AI systems [28,49,60]. In terms of dietetic interventions, the benefits of AI technology encompass providing more personalised and precise nutrition interventions [18,24,26,27,44,56,57,60,88,92,93,94,102,107,113] that are more tailored to specific chronic disease populations’ needs [18,24,44,57,60,88,92,93,107], assisting with brainstorming a greater variety of actionable recommendations to suit patient needs in order to support increased adherence [26,56,57,60,62,75,88,92,93,94,102,113] and utilising data-driven insights to predict patient outcomes for more tailored interventions [18,40,54]. AI-integrated technologies also have the capacity to empower patients to take an active role in chronic disease management through increasing engagement and self-awareness and building relevant nutritional knowledge and skills to support self-efficacy [28,38,43,44,45,46,52,60,61,64,66,75,91,93,94,95,96,98,99,101]. The chatbots employed in these studies were generally easily accessible to the public via the internet (e.g., ChatGPT versions 3.0, 3.5, and 4.0, Bard AI, and Bing Chat). In other cases, innovations that assisted with interventions had embedded recommender systems within a digital web platform or smartphone app interface for users to interact with. These systems generally provide recommendations to patients for menu or recipe ideas, with considerations of evidence-based guidelines for their chronic disease management and personal taste and cuisine preferences. For instance, a 2024 study combined both patient profile data (e.g., anthropometric values, past medical history) to formulate nutrient requirements to guide ChatGPT’s output of culturally tailored and accurate meal plans for chronic disease patients [88]. 

The integration of food recognition coupled with recommender systems also unlocks the potential for AI-integrated technologies to assist with both nutrition intervention alongside monitoring and evaluation. For instance, studies that focused on mobile apps generally had food recognition and recommender systems to underpin app functionalities to target specific clinical conditions (e.g., liver disease, irritable bowel syndrome (IBS) and low fermentable oligosaccharides, disaccharides, monosaccharides, and polyols (FODMAP)) [52,64,93]. In turn, this would increase patient dietary awareness and reinforce nutrition education for the intervention phase. Similarly, smart utensils were a new AI-integrated technological innovation explored by Nakaoka and colleagues (2021). Sensor-based chopsticks could be used to assist in providing patients with real-time feedback on the healthiness category of food consumed (i.e., whether food was healthy or not) and the rate of food consumption, which, in turn, actively reinforces the application of real-time nutrition education [81]. 

Anthropometric parameters were examined across twenty studies [18,23,25,26,38,40,45,46,50,63,64,69,75,82,85,94,98,99,108,109]. A number of these studies reported weight loss outcomes for metabolic health conditions such as obesity [18,45,75,82], type 2 diabetes mellitus [18,45,63,69,99,108], and Non-alcoholic fatty liver disease (NAFLD) [64]. Of these weight loss studies, three also reported a concurrent reduction in waist circumference in participants [45,75,108]. De Marchi and colleagues (2022) observed successful weight stabilisation, with the prevention of further weight loss in amyotrophic lateral sclerosis patients who engaged with an eHealth Chatbot platform compared to the control group that experienced a 3 kg weight loss over 6 months [38]. For significant weight management-focused studies, AI-integrated technology provided users with real-time interactions via chatbots [38,75] and food intake tracking [23,45,50,63,64,69,82] and recommended advice or meal ideas [63,69,82]. Five studies explored how AI systems could assist with data-driven insights to predict an individual’s weight trajectory and provide accompanying meal recommendations [18,94,98]. Health professionals gauged weight management program engagement and efficacy [40] and nutrition support for malnutrition in aging populations [109].

Biochemical and microbiological outcomes changes in participants’ biomarkers or as data integrated within AI-integrated technologies were reported across fifteen studies [24,26,44,45,56,57,60,63,64,69,82,85,99,107,108]. A study conducted by Kiriakedis and colleagues (2024) observed that ChatGPT (Version 4.0) exhibited poor performance in detecting and advising on abnormal urinalysis calcium and citrate levels, as it was unable to address 30% of the abnormalities presented. However, it exhibited strong performance for interpreting urinalysis biomarkers within normal levels [60]. Six studies highlighted overall improvements in glycaemic control [44,45,63,69,99,108]. Of these, four studies specified reductions in HbA1c levels using AI-based smartphone apps [63,69,99,108]. Kwon and colleagues (2024) observed significant improvements with respect to liver function test biomarkers (AST, ALT, and ɣ-GT) in non-alcoholic fatty liver disease patients as they developed health-related self-management skills over 6 months with the assistance of a nutrition advisor integrated in the SMART-liver app [64]. A key study unveiled that engagement with a generative pretrained transformer for dietary guidance significantly reduced the proportion of haemodialysis patients with hyperkaliemia from 39.8% to 25% [56]. Three studies exploring precision nutrition highlighted alterations in patient gut microbiome populations [24,57,107]. Two studies reported that greater favourable changes to microbiota occurred with increased adherence to the personalised diet, as opposed to the conventional Mediterranean [24] and low-FODMAP diets [107]. Similarly, Karakan and colleagues (2022) asserted that a personalised diet tailored for the gut microbiome of each IBS case led to increases in *Faecalibacterium* genus, *Bacteroides*, putatively probiotic genus, and *Propionibacterium* populations [57].

Clinical symptom-related outcomes were explored in fourteen studies [26,27,28,33,45,54,57,64,75,85,93,94,99,107]. The studies centred around patients with IBS and diarrhoea-predominant IBS (IBS-D) (*n* = 2) [54,57] and all IBS subtypes [93,107]. Of these, one study reported improved symptom management for participants receiving the AI-assisted personalised diet for all IBS subtypes compared to the FODMAP diet, which was only effective in the management of symptoms for IBC-C and IBS-D subtypes [107]. Across these studies, it was observed that 81% of participants with IBS/IBD had symptom improvements at week 5 [54], while another study reported a change in the categorisation of IBS symptoms from severe to moderate over 6 weeks [57], both of which utilised AI recommender systems. Buchan and colleagues (2024) unveiled significant improvements in quality of life and symptom management for 82% and 88% of cancer patients, respectively, after interaction with INA, a virtual assistant platform [27]. A study on type 2 diabetic patients using Twin Precision Nutrition observed significant improvements in metabolic health markers (e.g., Hba1C, weight, HOMA-IR), with most patients ceasing the use of medications (including insulin, metformin, DPP-4 inhibitors, and liraglutide) [99]. However, one study noted reduced confidence with diabetes management after using web-based diabetes nutrition care platforms that meal preparation and planning [28].

Sixty studies reported diet quality-, nutrient-, and food intake-related observations [20,21,22,27,28,29,30,33,35,37,38,41,43,44,45,46,49,50,52,53,55,56,57,58,59,60,61,62,64,66,68,70,71,74,75,76,77,78,79,80,82,83,84,85,86,88,89,90,91,92,93,94,95,96,97,98,99,100,101,102,103,104,105,106,110,113]. For more patient-focused studies, nine studies explored alterations to dietary intake and behaviours. Of these, two studies helped with improving participant confidence in core food preparation skills, such as meal planning [28] and meal preparation and cooking [43]. Many study participants were receptive to AI-integrated technologies, which was observed through an uncovered increase in tendency to select healthier choices [45,46,96,101] and more regulated portion sizes [46,101] and improvements in food intake patterns (e.g., reduced snacking) [33,96]. More specifically, two studies on participants interacting with Paola (an AI virtual chatbot on the Slack communication platform) observed improvements in the selection of foods, leading to greater dietary compliance with prescribed diets, such as the Mediterranean diet [37,75].

Alternatively, some studies focused on evaluating the quality-of-food-related outputs from AI systems [29,49,56,61,71,76,79,80,83,89,90,92,94,104,106,110]. For studies that focused on AI system recommendations, some utilised healthcare professionals or experts’ responses as a standard of comparison, including clinical diabetes educators [29], dietitian clinical judgements and recommendations [61,71,76,104], Mediterranean diet adherence scores by dietitians [90,110], and nutrition experts [94]. Alternatively, studies also compared AI outputs to recognised dietary standards for the keto diet [104], allergy diets [83], Mayo Clinic Renal Handbook [56,92], National Dietary Reference Intakes (USDA DRI) [49], and various international clinical guidelines for management of NCDs [80,91]. For some studies exploring AI’s capabilities with respect to macronutrient estimation, the AI system outputs were compared to validated tools such as the ASA-24 [79], dietitians carrying out 24 h dietary recalls [89], and nutritionists [106]. Studies on wearable devices commented on performances in detecting important stimuli indicative of eating occasions, such as sound patterns [20,21,22,30,68,86], movements associated with fluid intake [22,58,59], and food intake gestures [30,41,58,59,97].

#### 3.3.4. Monitoring and Evaluation

Eight studies solely focused on patient monitoring and evaluation, which mostly incorporated food recognition technologies, some recommender systems, and the reduced involvement of chatbots. Of these AI-integrated technologies, they were predominantly embedded in smart device-based apps and platforms (*n* = 6) [36,37,50,65,69,84], and the remaining AI-integrated technologies explored were wearable devices (*n* = 2) [21,51].

Fifteen studies monitored patient adherence. Of these studies, greater levels of engagement and retention and lower dropout rates were observed in studies which implemented Chatbots in their platform (*n* = 4) [27,38,63,75], AI biochemical data-driven personalised nutrition (*n* = 2) [54,107], meal recommendation apps (*n* = 2) [26,43], and apps with a health behaviour coach feature (*n* = 4) [46,63,64,75]. Lower engagement and adherence were seen with respect to dietary support apps or web-based platforms (*n* = 4) [26,28,55,102] and food recognition systems (*n* = 1) [89]. Changes in perception or interest in health outcomes over time (*n* = 2) [26,55], use in older or chronically ill populations (*n* = 1) [28], compliance with instructions (*n* = 1) [89], and the necessity for regular reminders and support follow-through during intervention delivery (*n* = 1) [102] were some of the factors contributing to reduced engagement with AI-integrated technologies.

#### 3.3.5. All Stages of the NCP 

Ten studies explored AI-integrated technological innovations with the potential to support all four stages of the NCP (Table 4) [38,54,66,79,80,94,99,101,104,109]. Most of these digital technologies utilised recommender systems [54,66,80,94,99,101,104,109]. For instance, a Personal Intelligent Nutrition (PIN) system designed to automate patient health assessments and meal plans received 88.7% agreement from nutrition experts for weight and body fat percentage recommendations and was commended by experts due to its ability to identify nutrients of concern and the meal plans that address them [94]. 

Two studies explored recommender systems paired with image or audio recognition in the form of nutritional intake platforms [66], which could aggregate user-reported data and external sources to provide seamless accessibility to nutritional care, and technology-assisted dietary assessment mobile apps that improved the user’s diet quality and eating behaviours [101]. De Marchi and colleagues’ (2022) e-Health chatbot app encouraged patient adherence with respect to registering dietary intake, and they supported dietitians in prescribing a personalised caloric dietary plan [38].This resulted in weight stabilisation in amyotrophic lateral sclerosis patients, with only 3 kg weight loss over 6 months compared to the baseline in the control group [38]. Only Sun and colleagues (2023) utilised all three forms of AI systems (food recognition, chatbots, and recommender systems) to propose an AI nutritionist program based on ChatGPT and a generative pre-trained transformer to provide easy and affordable access to dietetic care and all stages of the NCP [104]. 

### 3.4. Efficiencies of AI-Integrated Technology Use

Ninety-three studies [18,19,20,21,22,23,24,25,26,27,29,30,31,32,34,35,36,37,38,39,40,41,42,43,44,45,46,47,49,50,51,52,53,54,55,56,57,58,59,60,61,62,63,64,65,66,67,69,70,71,72,73,74,75,76,77,78,79,80,81,82,84,85,86,88,89,90,91,92,93,94,95,96,97,98,99,100,101,102,103,104,105,106,107,108,109,110,111,112,113,114,115] commented on potential efficiencies in utilising AI-integrated technologies compared to traditional methods of dietetic care. Another notable benefit for both dietitians and patients was the reduced burden in collecting and analysing food intake records for dietary assessment (*n* = 25) [18,20,22,33,34,53,55,59,66,70,72,73,74,75,78,79,81,82,90,97,100,106,110,111,114] and monitoring and evaluation (*n* = 29) [18,22,35,38,39,41,47,48,51,55,56,57,58,63,64,65,66,67,70,78,81,84,85,86,90,101,108,111,114].

More specifically, for dietitians, AI-integrated technologies could potentially improve the flow of consultations by supporting clinicians in summing up all relevant data to support decision-making processes for nutrition assessment and delivering tailored nutrition interventions (n = 20) [23,32,53,54,55,56,59,60,69,76,78,79,80,88,94,102,109,110,111,114]. Through the patient perspective, benefits of implementing AI-integrated technologies included easier access, use, and interaction across smart devices (e.g., smartphones, iPads or tablets) (*n* = 21) [19,25,27,31,43,46,48,55,60,61,71,80,82,91,93,95,98,101,103,104] and relevant nutrition-related information or feedback on demand (*n* = 14) [25,52,55,62,82,88,90,91,92,93,95,96,98,111]. AI-integrated technologies were also cited to be cost-effective (*n* = 13) [19,55,56,59,61,77,80,82,85,91,102,104,108] for accessing nutrition care and managing chronic health conditions to minimise long-term healthcare costs. In more regional or rural areas or locations, there are limited healthcare resources (e.g., healthcare professionals), and AI-integrated technologies assisted in extending dietetic care to individuals who needed it (*n* = 10) [27,31,45,60,71,76,91,95,102,104].

### 3.5. Limitations and Safety Concerns with AI-Integrated Technology Use

Among the six studies (Table 5) [19,27,72,80,83,104] that mentioned patient safety, two studies touched on the provision of warnings and disclaimers with ChatGPT responses [19,80], and four studies encouraged further verification via seeking professional advice [80,91,92,104].

Studies also emphasised that, depending on how the user phrased their prompts (*n* = 4) [19,49,80,104], the time and date of prompt entry (*n* = 2) [60,80], and the complexity of the cases (e.g., presence of multiple chronic conditions or food allergies) (*n* = 3) [27,61,91], chatbot systems could potentially provide inconsistent or misleading responses. Therefore, it was highlighted that individuals without prior nutrition knowledge were vulnerable to nutritional misinformation (*n* = 7) [19,25,60,61,71,80,91]. For instance, a study utilised ChatGPT to generate allergen-specific menu and food recommendations, which incorrectly integrated almond milk into a nut-free allergy diet [83]. Furthermore, concerns were highlighted regarding how virtual assistants may be limited in being able to safely offer additional guidance for more complex patient cases with multiple chronic medical conditions, food intolerances, or allergies [27], and discrepancies were observed between chatbot food item recommendations and best practice guidelines for specific diets [104]. Professional oversight by dietitians was integrated across some studies to mitigate these safety concerns [27,83,104]. Lin and colleagues (2020) advised that employing recognised nutritional guidelines (i.e., European Food Safety Authority (EFSA)) into AI systems would further assist and guide a more accurate and robust learning recommendation system [72]. Some authors emphasised that refining these AI-integrated technologies should be a collaborative task between dietitians, nutrition experts, or other healthcare professionals to ensure appropriateness for clinical use (*n* = 5) [70,71,80,83,90].

Across the AI-integrated technologies explored, some were still in the preliminary stages of prototype development and were only trained either using smaller datasets or a specific set of clinical nutrition guidelines (*n* = 22) [24,28,33,34,35,37,49,52,54,56,65,69,71,77,88,92,95,100,109,111,113]. In turn, some studies suggested that their AI systems undergo further refinement with larger datasets to address low varieties in certain food groups or mixed-food selections and recommendations (*n* = 8) [34,46,55,56,62,89,94,98], to include different cuisines (*n* = 4) [26,56,104,106], to cover more nutrition-specific diets (*n* = 2) [18,62], and to account for dietary supplements (*n* = 1) [79]. Studies also noted factors such as user self-desirability bias (n = 1) [29] and user confidence (*n* = 1) [110], alongside underreporting (*n* = 1) [79] or omissions (*n* = 1) [27] by AI systems requiring self-reported outcomes. 

More specifically, for wearable sensor systems, studies highlighted limiting factors, including poor performance in deciphering food attributes and intake (*n* = 6) [20,21,47,68,70,86], fluid intake detection (*n* = 5) [36,39,47,59,86], and prototype design impacting the user and their comfort (*n* = 5) [22,51,68,73,86], or they were more sensitive to interference by surrounding factors (*n* = 5) [72,78,87,97,112]. Additional barriers to user engagement included the presence of technical jargon (*n* = 3) [25,46,60] and poor design of interactive features (e.g., generic message outputs) (*n* = 3) [26,50,92] in relation to the user interface. Other prominent barriers cited include access to the appropriate technology (e.g., specific smart device, internet access) (*n* = 3) [45,48,64], poor technological literacy (*n* = 2) [70,75], and elderly populations (*n* = 2) [33,85].

### 3.6. Ethical Considerations

Eleven studies commented on ethical considerations relating to patient privacy in AI-integrated technologies (Table 5) [19,25,36,50,56,85,92,97,99,105,108,111]. Two studies explored alternative privacy-preserving designs for wearable devices. These included the use of depth cameras [36] and the repositioning of camera angles [97] to minimise the collection of unnecessary surrounding images. Further studies on wearable sensor systems highlighted the potential of sharing unwanted images or other sensitive information, such as GPS location, with third parties (e.g., annotator, human observer) to assist in training AI systems for image recognition functions [31,50]. One study had sharing-based authorisation, whereby patients could grant healthcare professionals access to their wearable device’s data [111]. Additionally, one study raised concerns with respect to the anonymity and privacy of the user when utilising a health chatbot to provide information-based support and answers [25]. 

Three studies explained that participants’ data would be transmitted and stored in a security system to protect them [85,99,108]. These included incorporating layers of security to control users’ and administrators’ access, restricting the collection of personal identification information [85] and providing a secure transmission pathway [99,108]. Two studies raised the importance of the development of standardised ethical guidelines on patient autonomy and informed consent [56,92]. In contrast, an additional three studies advocated for the importance of warnings and disclaimers for potential AI errors that could compromise patient safety [19,27,80]; hence, they advocated for the value of healthcare professional oversight to mitigate this risk [19,27,72,80,83,104,116].

## 4. Discussion

Embedding AI-integrated technologies into the NCP presents a promising opportunity to address rising healthcare demands driven by the growing prevalence of chronic health conditions [8,15]. The salient findings include the following: (i) three main AI systems (image or audio recognition, chatbots, and recommendation systems) being employed; (ii) AI technologies could be integrated into one or more stages of the NCP to aid dietitians with workflow and patient outcomes; and (iii) AI-integrated technologies potentially enhance patient outcomes for chronic disease management through strengthening patient engagement and targeting drivers of behavioural change. Nevertheless, AI-integrated technologies are not a replacement for dietetic expertise, given their susceptibility to errors which can impact patient safety and ethical concerns.

### 4.1. Nutrition Assessment

The AI-integrated technologies in our review have largely targeted the nutrition assessment component of the NCP. Numerous studies have reported that AI-integrated technologies are a solution for alleviating the burden for both patients and dietitians in collecting data for dietary assessments, as this constitutes a significant proportion of initial consultations [71]. AI food and audio recognition systems are time-efficient and seamless for recording food intake and calculating the corresponding nutrient composition [117]. Prerecorded dietary intake can be more readily evaluated during dietary assessments and diagnosis stages, thus allowing more time to be allocated to targeted nutrition education and behavioural counselling. Such types of AI systems have mostly been embedded in wearable sensor systems (e.g., necklaces, wristbands, glasses) and utensils or incorporated in smartphone or web-based apps. These systems have demonstrated the ability to accurately detect food intake (e.g., solid foods) [22,41,59]. Additionally, AI addresses the shortfalls of conventional dietary assessment methods (e.g., using 24 h recalls for diet history), which are susceptible to biases relating to memory, measurement, and under-reporting due to social desirability [118]. However, the accuracy of wearable or sensor-based AI-integrated technologies remains inconclusive, as many examples included in this review were in the preliminary stages of development and may have only been tested in laboratories (e.g., [20,22,30,34,48]). Further development of AI-driven image and audio recognition systems requires fine-tuning to address detection inaccuracies, such as fluid intake and identifying variable food attributes (irregular shape, lower weight, and soft foods), to better capture and reflect an individual’s daily dietary variability.

AI food recognition algorithms have expanded beyond use in nutrition research and become increasingly integrated into commercially available apps (e.g., MyFitnessPal) [70]. AI algorithms can leverage food composition and nutrient databases to enhance the accuracy and efficiency of individual dietary intake analyses [8,12]. For instance, the Keenoa app demonstrated strong comparative validity relative to conventional methods for recording dietary intake and nutrient estimation [55,78]. Participants preferred app-based food intake recording over more traditional methods (three-day food diary and ASA-24) due to reduced self-burden with increased ease and convenience for food logging [55,78]. However, dietitian oversight is still required to appraise the app’s nutritional assessment, override errors, and manually enter more complex food items (e.g., dietary supplements) [55,78]. While these AI-integrated technologies may exhibit greater accuracy, adaptability, and efficiency, their degree of performance and their reliability have limitations. They are inherently dependent on the quality and comprehensiveness of their underlying databases. Therefore, it is crucial to dedicate additional efforts to optimising nutrition databases, expanding them to include cultural foods, cooking styles, and branded pre-packaged items [70]. AI integration automates dietary data collection and analysis, streamlining the assessment process. Consequently, dietitians can focus on building rapport and trust with patients to better implement motivational interviewing and behavioural counselling techniques [8]. 

### 4.2. Nutrition Diagnosis

Nutritional diagnoses are essential for guiding appropriate, targeted interventions. Although this review uncovered only one suitable AI diagnostic system for primary care, AI-integrated technologies have been widely explored for acute inpatient settings. For instance, a trial on machine learning models using electronic medical record (EMR) admission data (e.g., biochemical tests, anthropometry, disease severity scores, nutrition assessments) has been designed to predict malnutrition diagnoses in accordance with the Global Leadership Initiative on Malnutrition (GLIM) diagnostic framework [119]. Similar studies may serve as examples for where AI algorithms can be adapted for primary care clinical data. However, the development of a truly representative diagnostic tool is a challenge as nutrition-related diseases arise from a complex interplay of health and lifestyle factors. In primary care, patients present with a variety of more unique and complex chronic conditions (e.g., multiple complex comorbidities, allergies, and intolerances). Based on this review, there is limited evidence for the accuracy of AI-integrated technologies in formulating nutritional diagnoses for various clinical conditions [32]. Instead, this review provides insight into how AI systems can support dietitians by presenting data aligned with the most relevant clinical diagnostic tools, frameworks, and guidelines. This approach streamlines the process of identifying a list of potential diagnoses. Dietitians can select the most appropriate diagnosis while considering the patient’s social, socioeconomic, and environmental context. Through informed clinical decision-making, dietitians retain professional autonomy within an increasingly AI-integrated healthcare environment. In the diagnostic domain, AI-integrated tools are supportive adjuncts, offering data-driven insights that align with clinical practice guidelines.

### 4.3. Nutrition Intervention

AI has created new opportunities to advance precision nutrition by leveraging patient-specific data, including genetic, metabolic, and physiological parameters and gut microbiota profiles [9,15,88,120]. Personalised diet programs like ZOE have gained interest for delivering tailored nutrition advice based on multiple biochemical inputs (blood lipids, blood glucose profile, and gut microbiome) collected using convenient at-home test kits [121]. In the 18-week ZOE Measuring Efficacy Through Outcomes of Diet (METHOD) RCT, ZOE was more effective in improving the markers of cardiometabolic health (body weight, waist circumference, HbA1c, and triglycerides) compared to generalised dietary advice (United States Dietary Guidelines for Americans 2020–2025) [122]. Precision nutrition intervention participants received personalised food scores in conjunction with generalised nutrition and lifestyle education remotely via the ZOE app. Tailored recommendations from ZOE’s algorithm (2022) considered various factors, including food characteristics, biochemical data from at-home collection kits, cardiovascular disease risk, and past medical history [122]. Similarly, this was echoed in this review, where precision nutrition yielded significant clinical improvements for patients over more conventional dietary pattern prescriptions (e.g., Mediterranean diet, low-FODMAP) [57,99,120]. However, like ZOE, AI-driven precision nutrition remains largely confined to clinical trials and higher socioeconomic groups, limiting its accessibility and equitable reach among the general population [120]. Rapid advancements in precision nutrition have warranted its spotlight in the NIH 2020–2030 Strategic Plan for Nutrition Research. This plan further advocates for the use of AI and machine learning to more effectively integrate genetic, microbiome, behavioural, and environmental data to generate individualised dietary recommendations [123]. The Australian Academy of Science’s decadal plan also recognises precision nutrition as one of the four pillars of nutrition science, elucidating its potential to tailor dietary guidance in accordance with an individual’s biology, culture, and context [124]. With continual refinement, AI systems will become better equipped to deliver more precise and effective recommendations for the wide variety and complex, multifactorial nature of NCDs treated in primary care [15,88].

This review also examines AI-integrated apps that offer greater accessibility to the population than precision nutrition. Our findings highlight that these technologies support patients in improving patient dietary intake and health outcomes, with predominantly positive implications for weight loss [23,63,69,82], gain [18] and stabilisation (e.g., in patients with amyotrophic lateral sclerosis) [38]. Further software refinement and up-scaling trials will enable some of these innovations to extend their reach and ease of access to dietetic care in regional, rural, and underserved areas with constraints in healthcare professional availability [91,104]. mHealth apps commonly integrating one aspect or a combination of image recognition, chatbots, and recommender systems better enabled users to receive real-time feedback. For instance, the eTRIP weight management program integrated an adjunct mHealth app with personalised reminders, chatbots providing real-time feedback, and food logging with image recognition [33]. Additionally, the integration of an appealing and user-friendly interface enhances engagement and adherence to dietary recommendations [33]. This builds upon Salas-Grove and colleagues’ (2023) review, reinforcing that mHealth nutrition apps with self-monitoring tools, personalised feedback, goal setting, educational content, reminders, prompts, and social support features (e.g., connection with peers or healthcare providers) pave the way for sustained behaviour change and, in turn, improve health outcomes in chronic disease populations [125]. AI-integrated technologies could be a cost-effective method of reducing the long-term economic burdens of chronic conditions, enabling individuals to seek necessary healthcare guidance for effective self-management rather than a reliance on later-stage medical treatments [126].

Generative AI chatbots such as ChatGPT are a publicly accessible tool that could be valuable as a complementary aid for patients beyond or outside clinical consultations; however, they are not a replacement for dietitians or clinical advice. Caution should be exercised as chatbots have been observed to mislead users on their credibility and accuracy, as factually incorrect outputs have been generated in a confident manner (known as “hallucinations”) [80]. When supporting intervention delivery, chatbot use requires oversight by a dietitian to assess the quality of outputs. AI chatbots may unlock the potential to design personalised educational materials or appropriate food recommendations to better tailor diets to patient preferences (e.g., taste, cuisine preference) [8]. When used in conjunction with clinician counselling, adjunct chatbot interaction enhances nutrition interventions through targeted nutrition education and recommendations (e.g., diet-compliant meal plans). This approach not only reinforces dietary prescriptions beyond consultations but may improve patient receptiveness to behavioural changes and adherence. A similar case was explored with respect to physiotherapy, elucidating the current limitations of publicly accessible AI. The study examined ChatGPT’s ability to offer advice in accordance with clinical guidelines, but it lacked nuanced clinical reasoning for more complex patient presentations and, thus, should only be used as a supplementary tool [127]. Hence, further research efforts may explore designing patient-facing chatbots based on evidence-based guidelines, and recommendations would ensure greater degrees of patient safety and efficacy rather than relying on publicly accessible AI chatbots. Mistakes in healthcare lead to detrimental harm, and clinical experience and expertise are crucial safeguards for addressing patient-specific concerns, monitoring clinical progress, and ensuring patient safety by clarifying misinformation [70,80,91,92,116].

### 4.4. Monitoring and Evaluation

A key component mediating the effectiveness of the prevention and management of chronic health conditions is patient engagement and adherence to prescribed interventions [128]. Our findings indicate that a prominent overlap occurs in the AI-integrated technologies designed to target the NCP stages of nutrition intervention, monitoring and evaluation, and dietary assessment (recording food intake). Various AI-integrated technologies, ranging from virtual health chatbots to smartphone apps designed for diet management, are tools that have been shown to facilitate higher levels of patient engagement [120,129]. Greater levels of patient satisfaction, engagement, and adherence were observed with AI-integrated technologies featuring chatbots [27]. This review offers a glimpse into the features that facilitate positive user experiences, which include app interfaces that are well-tailored to the needs, interests, and lifestyle of the user. For instance, the HealthAware advice desktop platform was designed to target office workers. Through active engagement with HealthAware, a majority (>50%) of users developed greater awareness of their current dietary patterns (e.g., snacking or selecting unhealthy food choices) and were more receptive to healthier dietary recommendations suggested by HealthAware. AI’s ability to reflect real-time analytics, optimising goal setting and tracking, facilitates patient engagement [75]. Additional AI-driven features, including reminders and nudges, sustain motivation and prevent behavioural relapse, targeting the user’s adherence and sense of accountability [33,81,102,125]. 

From a dietitian’s perspective, the emergence of generalist clinical AI systems capable of addressing three or more domains of the NCP will have broad implications. These include AI-integrated technologies designed with image or audio recognition and recommender systems that streamline the collection of dietary intake data and summarise it in relation to patient progress and goals. For instance, the clinical decision support system (CDSS) minimised the time required for clinicians to analyse patient self-reported dietary intake data via an app and offered clinicians dietary recommendations based on patient input data [29]. In turn, AI can bridge critical gaps limiting patient-centred care and would be very beneficial during follow-up consultations where time is more limited.

### 4.5. Additional Practical Implications

#### 4.5.1. Healthcare Professionals

Balancing clinician benefits, professional identity, and risk of deskilling are essential as AI becomes increasingly integrated in healthcare. Aspiring and current dietitians must develop a thorough awareness of the strengths, limitations, and risks of AI-integrated technology use. This is important as healthcare registration and accreditation bodies emphasise that healthcare professionals are responsible and accountable for the use of AI-integrated technologies in clinical practice and patient care. Key future learning initiatives include embedding course content regarding practical, safe, and ethical use of AI-integrated technologies in clinical practice into university health and medicine curricula (e.g., healthcare ethics, exposure in clinical rotations/placement or simulation clinics), professional development courses, or onboarding modules in healthcare workplaces [130]. In the technology acceptance model, training is a critical factor mediating perceived usefulness and increasing receptiveness through shifting mindsets and attitudes towards AI integration in healthcare [131,132]. Training initiatives to enhance dietitians’ self-efficacy in using mHealth tools may adopt a two-phase model comprising an initial education workshop followed by a supervised integration phase [130,133]. Rather than deskilling, AI’s growing presence presents an opportunity to upskill dietitians as their expertise and identity evolve, while still preserving professional autonomy.

Furthermore, EBP provides a clinical decision-making framework that integrates the best available research evidence and clinical experience and patients’ contextual values [134]. Limited time outside patient care and financial constraints are significant barriers to dietitians’ ability to remain up to date with the latest clinical research for EBP [135,136]. AI-integrated clinical technologies are a practical solution, enabling dietitians to efficiently strengthen their clinical dietetic knowledge. AI chatbots can improve accessibility, facilitate the synthesis of large research databases to enhance confidence in knowledge translation, and apply emerging evidence in practice. Clinician-facing AI-integrated technologies such as the 2023 Mayo Clinic Platform Accelerate Program, OpenEvidence, have been evaluated using primary care cases (hypertension, hyperlipidaemia, type 2 diabetes mellitus, depression, and obesity) to deliver pertinent outputs that support a physician’s clinical judgement in medical decision-making rather than replacing it [137]. OpenEvidence’s design promotes transparency in its outputs by incorporating reputable journal citations (e.g., New England Journal of Medicine, Journal of American Medical Association). This fosters clinician trust and reinforces a collaborative clinician–AI working environment in which clinicians retain autonomy to act on or reassess AI-generated recommendations. For dietitians, adapting a similar system could enhance point-of-care support, especially given the breadth of clinical presentations in primary care.

#### 4.5.2. Reimbursement Models

The emergence of AI-integrated technologies presents both opportunities and challenges for achieving equitable access to healthcare, particularly dietetic services. Compared to larger healthcare precincts (e.g., metropolitan), there is an underutilisation of AI-integrated clinical technologies in smaller, regional, or rural health services. This imbalance is driven by differing capacities and resource availability (e.g., workforce shortages, financial constraints, and infrastructure). Instead, AI-integrated technologies should be regarded as a cost-effective solution to address these shortcomings [45,91]. Generalist AI-integrated technologies currently being trialled in medicine are capable of engaging multi-step integrated analysis and thus present a promising opportunity to ensure that healthcare services can be equally accessible regardless of location [138]. Similarly, this review highlights potential generalist AI-integrated technologies that can support all stages of the NCP.

However, outside of certain medical specialties (such as radiology and pathology), the deployment of AI-integrated technologies has generally been slower [139,140]. A primary barrier for its slower adoption in healthcare stems from uncertainty with respect to evidence supporting the accuracy, value, and utility of AI-integrated technologies. Implementation requires considerable costs for primary care organisations: for example, purchasing software licenses, adequate professional training, digital infrastructure adjustments, and continual maintenance and monitoring. Current reimbursement models in primary care (a fee-for-service payment of discrete definable medical services and episodes of patient care) are not reflective of the capabilities of AI and the evolving roles of healthcare professionals [138]. Additionally, given the ongoing development of AI-integrated technologies, it would be limiting for dietitians to restrict their use to a single tool or a select few. A proposed revised government-funded healthcare model (e.g., Medicare, National Healthcare System) articulates three guiding principles: 1. bundling AI services with complementary services; 2. setting a separate reference fee or transitional add-on payment for AI-enabled services; 3. regular re-evaluation of Medicare fees to reflect cost fluctuations and the associated value of clinical work [141]. Future planning for widespread AI integration should draw on the successes and challenges experienced during the global transition from paper-based patient records to EMR systems.

#### 4.5.3. Patient Safety, Efficacy, and Evaluation Tools

A lack of consensus exists on how AI-integrated technologies could be evaluated for patient safety and efficacy and the identification of accountable stakeholders. There are multiple means of evaluation, yet no standardised benchmark, tool, or reporting guidelines exist. For instance, the current evaluation metrics used include consumer (patient or clinician) usability ratings and business operation key performance indicators (e.g., time efficiency, patient turnover rates). Whilst these metrics have largely highlighted the positive impacts of AI-integrated technologies, some reveal safety concerns, including advice that may be harmful or that conflicts with nutritional guidelines. This underscores the need for a thorough investigation into current evaluation approaches. Governing regulatory bodies such as the Food and Drug Administration (US) and Therapeutic Goods Administration (Australia) oversee the clearances of medical devices fit for consumer use. Although certain types of AI-integrated technologies fall within existing regulatory frameworks, others may fall outside their scope and be overlooked, posing a risk to patient safety. Future efforts should focus on developing standardised criteria or frameworks for AI-integrated healthcare technologies. These frameworks should be able to provide the following: 1. clear definitions of the intervention and context of use; 2. specify and explain the mechanisms underlying the AI system; 3. capture relevant health outcomes and infer the effect or causality of the AI-integrated technology [139]. 

RCTs are the gold standard for inferring causality. However, RCTs may not be feasible due to limitations (e.g., increased time requirements, funding). Only a small fraction of AI-integrated technologies were evaluated using this type of study design (Supplementary Table S6). Instead, for primary care settings, cohort observational data (e.g., based on wearable sensor data) and quasi-experimental designs may be more practical with some additional considerations (e.g., training participants to accurately collect data, additional statistical expertise for data cleaning) [139].

For quality evaluation, ongoing efforts are underway to adapt and extend pre-existing tools, statements, and guidelines, including APPRAISE-AI, CONSORT-AI, TRIPOD + AI, and the MARS tool [139,142,143,144,145]. All these efforts can contribute to consumer safety certifications and mandated labelling processes which currently apply to products such as medications, foods, and medical devices. Product specifications should include a clear overview of designs, intended use, and performance (reliability, safety, compliance, and monitoring), enabling end-users to be better informed.

#### 4.5.4. AI-Integrated Technology Development

In this review, some studies on AI-integrated technologies were in stages of prototype refinement and preliminary testing, accounting for differences in relative performance. A lack of a standardised framework for protocol development, training, and testing with respect to AI-integrated technologies targeted for the healthcare industry may lead to poorly designed systems [129]. Various studies indicated differences in foundational AI algorithms and models, the quality of training datasets (sample sizes and representativeness), and mechanisms. Absence of design transparency poses difficulty for peer-reviewed assessments of AI output quality and reproducibility. In cases where dataset quality is overlooked, AI algorithmic biases can continue to perpetuate pre-existing healthcare disparities (e.g., variation in health condition diagnoses across different sociodemographic groups), especially when deployed on a system-wide scale [146]. Clinician involvement in the development of AI-integrated technologies is often limited, yet it is critically important [126,129,147]. Only two-thirds (68%) of the studies in this review reported dietitian, nutritionist, or nutrition expert involvement. Dietitians bring a nuanced understanding of clinical nutrition, clinical workflows, and real-world constraints [120,129,147]. Clinicians contribute significantly to patient-facing care. Thus, they are well positioned to advocate for patient feedback, which can be under-represented in digital healthcare development. Moving forward, all stakeholders play pivotal roles in successful integration and quality improvement cycles as AI-integrated technologies continue to evolve.

Early responses by the World Health Organisation included the launch of the “Ethics and Governance of AI for Health” (2021) manual. Internationally, countries have adjusted and reviewed patient privacy legislations, such as HIPAA (United States of America); GDPR (Europe); DCB0129, DTAC, DSPT, Cyber Essentials, and the Data Protection Act (United Kingdom); PIPEDA (Canada); Australian Privacy Principles (Australia); and Information Privacy Principles (New Zealand) [126]. In this review, there were particular concerns regarding patient privacy, confidentiality of patient data that are collected and analysed by apps, web-based tools, and wearable sensor systems or used to train AI systems, and secure data storage (Table 5). To mediate this, the correct authorisation of access should be practiced, and patient data should be encrypted and anonymised [111,129]. However, there are other concerns as anonymised patient data can be nullified due to the ability of new algorithms to reidentify data [129]. Therefore, it is crucial that regulatory frameworks and legislation are continually revised in accordance with the rapidly evolving AI-integrated technology landscape.

### 4.6. Strengths and Limitations

Our review offers a novel focus on primary care settings. Primary care plays a critical role in relieving the burden on emergency medicine and inpatient care, especially with the growing prevalence of chronic NCDs as the current population ages. This differs from more recent reviews, which generally provided more generalised insight into AI-integrated technologies for nutrition research or acute care clinical practice (e.g., [9,14,129,148,149,150]).

The scoping review design was selected to capture the breadth and diversity of the literature on how AI-integrated technologies contribute to the NCP rather than assessing AI’s effectiveness. Following established frameworks [17,151], two independent reviewers conducted a comprehensive search across six health and technology databases. Through this approach, key healthcare ethical considerations were highlighted for future action through a collaborative effort by research, policy, and technology development stakeholders. 

However, as this is a scoping review, the included studies have not undergone a quality and bias assessment. Precautions should be exercised when interpreting individual study results due to the potential risk of bias within study methodologies and designs. Future research endeavours should consider a systematic review to assess study quality or a meta-analysis to enable statistical inferences to be derived for addressing the effectiveness of AI-integrated technologies with respect to patient health and behavioural outcomes. This can strengthen our understanding of the feasibility of implementing AI-integrated technologies in the NCP. 

Furthermore, some AI-integrated technologies found in this review were only in preliminary stages of development, and thus, individual findings may not be conclusive and generalisable. Across numerous studies, small sample sizes and the recruitment of specific populations were emphasised as limiting factors that impact the generalisability and conclusiveness of findings. Therefore, any transition and implementation of AI-integrated technologies demand thorough reassessment and reporting to ensure compatibility, safety, and appropriateness for clinical use. Additionally, our targeted focus on primary care excluded AI-integrated technologies that could streamline dietitians’ administrative workflow beyond patient consultations (e.g., billing, letter transcripts, EMR documentation). A significant proportion of a clinician’s patient care activities beyond consultations is often overlooked, thus warranting additional exploration of how AI-integrated technologies can also transform this space [152].

This review also does not explore AI-integrated technologies for inpatient clinical dietetics. Given the differences between acute inpatient care vs primary care, such as length of patient interactions (e.g., short vs. long term) and breadth of clinical presentations (e.g., malnutrition screening, refeeding syndrome risk, parenteral or enteral nutrition support vs. chronic disease management), this topic would be more suited as a separate avenue for future exploration. AI integration into inpatient clinical dietetics is also a concurrently evolving research field that addresses common dietetic presentations unique to critical care and hospital admissions. Notable AI-integrated technologies have addressed muscle mass evaluation, malnutrition screening, prediction of enteral nutrition needs and feed tolerance, and the risk of refeeding syndrome in intensive care unit patients by integrating diverse data streams from the EMR [9,153]. Nevertheless, concerns around interoperability and patient privacy continue to be echoed. Last of all, this scoping review was constrained to English-only articles, which may have impacted the breadth of the literature survey and the data analysed. It is recommended that future research incorporate studies published in other languages to uncover other potential AI-integrated technologies that may emerge.

## 5. Conclusions

AI-integrated technologies enhance how primary care dietitians engage with all stages of the NCP. AI-integration in wearables, sensors, apps, web-based tools, and software are revolutionising dietary tracking and monitoring and personalised nutrition interventions. For clinicians, AI-integrated technologies support summative data analysis and clinical decision-making particularly in nutrition assessment and diagnosis stages, allowing more time to engage patients in nutrition education and behavioural counselling. This shift facilitates improvements in adherence to recommended dietary patterns and clinical markers that promote better long-term health outcomes. Challenges that arise include implementing professional development initiatives to upskill dietitians and protect professional autonomy; revising current reimbursement models; ensuring ongoing clinician oversight for patient safety; and establishing standardised technology development frameworks and validation tools alongside new regulatory and legal standards. Addressing these concerns requires a multidisciplinary approach (including AI technology developers, clinicians, researchers, and patients). This actively promotes a collaborative and expanding clinician–AI ecosystem built on trust, transparency, and acceptance. AI-integrated technologies offer transformative potential in advancing primary care dietetics and optimising chronic disease management.

## Figures and Tables

**Table 1:** Inclusion and exclusion criteria incorporating the Population Concept Context framework. This table was applied throughout all stages of screening.

	Inclusion Criteria	Exclusion Criteria
Population	Human studies on adult participants (≥18 years old)	Participants < 18 years old Studies not involving human subjects (e.g., modelling studies)
Concept	Artificial intelligence (AI)	Does not include AI and/or its application
Context	Clinical dietetic care: nutrition care process (NCP) Primary care settings (e.g., outpatient clinics, care facilities, clinics, community health centres, private practice)	Does not address NCP or its elements in dietetic care Address non-nutrition related specialised care Not conducted within a primary care setting or ambiguous setting
Study Design Attributes	Any country of publication Published in the English language Peer-reviewed original research articles Primary research methods studies Publication period: 2007–2024 Full text available	Published in a language other than English Non-peer-reviewed or non-original research articles Studies using secondary research methods (e.g., reviews, editorials, reports, commentaries) Publications prior to 2007 Full text not available

**Table 2:** Details of interest that were extracted from included studies during the data extraction process.

Data Extracted	
Article details	Article citation, country of publication, discipline
Population characteristics	Age, sex, sample size, chronic medical condition
Concept characteristics (artificial intelligence (AI))	AI system (image or audio recognition, chatbot, recommendation) Type of AI (e.g., machine learning, deep learning, natural language processing) Digital technology platform for AI (e.g., smartphone app, web-based platform, wearable sensor system)
Context characteristics	Location of the study (e.g., country, healthcare/laboratory/free-living setting)
Primary outcome	Where and how AI-integrated technology can be used in the NCP
Secondary outcomes	Patient outcomes after AI-integrated technology use (e.g., dietary behaviour changes, adherence, changes to medical/health parameters, user experience) Efficiencies with AI use Barriers with AI use in patients Ethics, safety, and risks

**Table 3:** Summary of the characteristics and outcomes of included studies (*n* = 97).

Author	Medical Condition	Dietitian Involvement	User/Patient-Focused Outcomes (A: Anthropometry, B: Biochemistry, C: Clinical Symptoms, D: Diet and Food Related, PA: Patient Adherence)	Evaluation Metric	Efficiencies	Limitations/Barriers of Use
Abeltino et al., 2022 [18]	Weight management—overweight	Development of nutrition plans	A: 500 kcal energy deficit/day resulted in an average weight loss of −0.4 ± 0.2 kg/week. 500 kcal energy surplus resulted in an average weight gain of 0.77 ± 0.63 kg/week.	-	Seamless and time-efficient method as a food diary for dietary assessment and monitoring Minimises incidence of recall bias Personalised nutrition diet plan based on predicted weight and potential to integrate nutrigenomics and metabolomics	System lacks training in conditions where individuals have engaged in more extreme diets (e.g., keto diet) Burden on users for continuous data collection via requirement to wear smart band Requires access to the internet and smart phone
Agne et al., 2024 [19]	Weight management—obese	NS	-	-	Easy to comprehend and understand complex information (e.g., scientific papers) Free and easy accessibility	Requires users to be able to input well-formulated questions and have prior nutrition and dietetic knowledge to identify misinformation or misinterpretation in responses May not be conducive to behavioural change due to lack of emotional intelligence in chatbots Does not consider other diet-mitigating factors (e.g., budget, time, food availability)
Amft et al., 2007 [20]	Healthy	NS	D: Classification and determination of sounds patterns for different food types: soft foods (no changes in sound pattern in ordered sequence) and hard foods (clear sequential structure in sound pattern).	Accuracy: 80%	Potential use for food classification in dietary assessment	Cannot accurately determine all types of food, especially softer and fibrous structure foods due to sound patterns and sequence
Amft et al., 2009 [21]	Healthy	NS	D: Bite weight prediction using acoustic chewing recordings is a feasible approach for solid foods. Good food recognition results for individual chewing events. Foods with low bite weight had higher prediction errors.	Recall: 80% Precision: 60–70% Accuracy: 94%	Automatic dietary monitoring to reduce self-reporting burden for participants	Lubrication with foods (e.g., adding sauces and butter) or the deterioration of foods can impact accuracy
Amft et al., 2008 [22]	Healthy	NS	D: Body movements and chewing sounds could be accurately identified using on-body sensors; detection of drinking movements can be used to monitor fluid consumption and avoid dehydration.	Accuracy: Movement: 79% Chewing: 86% Swallowing: 70%	On-body dietary monitoring	Careful and correct application of wearable sensors required
Ben Neriah et al., 2019 [23]	Weight management—overweight, obese	NS	A: Utilisation of the food photo recognition feature embedded within the app was associated with greater % of weight loss. This effect was mediated by increased duration of use and more logged days. PA: Photo users logged more days (6.1 days) compared to non-photo users	-	Greater efficiency during dietary assessment stage for dietetic consults Improves awareness of portion sizes and amount of food intake. Arises as from the need to photograph foods prior to eating Interactive and motivation building	-
Ben-Yacov et al., 2023 [24]	Prediabetes	Dietary guidance during intervention and follow- up	B: Greater changes observed in gut microbiota composition with AI-designed postprandial targeting diet compared with the MED diet.	-	Personalised and precision nutrition: targets glycaemic control, cardiometabolic markers, and gut microbiome	Generalisability of current findings—RCT on small sample size Limited statistical power to support potential association between diet, microbiota, and clinical features and produce prediction models
Beyeler M et al., 2023 [25]	Weight management—gastric bypass	Provided nutrition counselling at bariatric centre appointments	A: Nil significant difference between the pre- and post-surgery patient groups over several months.	Usability score: 87/100 Usefulness: 5.28/7 Satisfaction: 5.75/7 Learnability: 6.26/7	User friendly, easily accessible, and practical Provision of information on a variety of topics (with detailed and correct answers) User anonymity	Concerns over privacy, data storage, and confidentiality Presentation of answers by the HealthBot may contain technical jargon. Not easily comprehended by users without nutrition-related knowledge
Bohn et al., 2024 [26]	Aging population (within healthy weight range)	NS	A, B, C: Use of app and meal recommender system over several months (field trials) did not result in any improvement nor deterioration. No significant changes in health biomarkers (anthropometry, blood pressure) in the target population. PA: Limited adherence (reduction in optimism and perception of healthy changes/outcomes) with increased time of intervention (>3 months).	Consideration of app use: Netherlands: 9% Portugal: 47%	Personalised meal recommendations to meet macronutrient requirements based on user’s details (age, gender, personal food restrictions, and preferences)	Needs further refinement for simple and user-friendly interface (e.g., amount and presentation of nutrition information and values) Motivation features needed to encourage engagement and motivation Refinements required to meal recommender to cater for cultural populations and age groups (e.g., elderly) Does not consider factors that influence food intake (e.g., social eating occasions, dependency or mobility in older populations)
Buchan et al., 2024 [27]	All cancer types—primarily genitourinary, gynaecologic, gastrointestinal, lung	Development of database for algorithm. Manual review and modification of algorithm responses for more complex cases/questions	C: Improved QoL in 82% and better symptom management in 88%. D: Overall beneficial impact on dietary intake. PA: High levels of engagement via testing questions to INA. User retention: 8.8 months; 84% applied advice to guide diet; 47% used recommended recipes.	User satisfaction: 94%	Improved patient access to personalised dietetic-related assistance	Not linked to health record data, with reliance on self-reported outcomes (potential for omissions in patient information when reporting baseline information)
Bul et al., 2023 [28]	Prediabetes, T1DM, T2DM	NS	No mean difference in reported general health status with the use of the platform. A: Reduction in weight and waist circumference. C: Participants were less confident in diabetes management after platform use. D: Participants felt more confident in meal planning and choosing healthy food choices after intervention. PA: Challenges in maintaining user engagement with chronically ill and older populations over longer time periods.	User experience ratings: No additional assistance required for use: 52% User-friendly: 38% Easy to use: 38%	-	Generalisability of current findings: small and potentially biased sample Participants engaging in trials were generally healthier
Burgermaster et al., 2020 [29]	T2DM	Conducted interviews, established a standard for comparison, final evaluation comments	D: Inference engine recommendation outputs aligned with clinical diabetes educator (CDE) narrative observations 74% of the time; 63% consistency with the CDE gold standard.	-	Comparable standard of performance to clinical diabetes educator	Self-desirability bias: self-reported values for diet and blood glucose
Chen et al., 2023 [30]	Healthy	NS	D: Feasible for detection of different types of foods through analysing different bite–chew combinations and hand gestures with accompanying wrist band device.	Accuracy: 93.3%	Socially acceptable wearable system (camera-free and glasses-like)	-
Chen et al., 2015 [31]	Hypertension	Provided meal proof standard values	-	Accuracy: 100%	Improved efficiency for dietitians by providing food recommendation ideas. Ease of access to system (e.g., from home) and, hence, reduces time to travel for dietetic consults	-
Chen et al., 2012. [32]	CKD— haemodialysis (protein energy malnutrition)	Gathering patient information including physical exam findings and diagnoses. Assisted with nutritional diagnosis guidelines to convert into programming rules	-	Rates of diagnosis: Correct diagnosis by expert system: 100% Human misdiagnosis: 3 times	Increased accuracy and reduced duration required to reach nutrition diagnoses by dietetic professionals	-
Chew et al., 2024 [33]	Weight management—overweight	NS	C: No significant improvements in anxiety-related symptoms. D: Week-long intervention improvements in overeating habits, snacking, self-regulation of eating habits, depression, and physical activity.	-	Food image recognition system: time-efficient and reduced user burden with logging food intake. Overall enhanced user experience App development considers population-specific determinants of overweight and obesity, allowing for increased applicability to this population	Generalisability of findings: conducted on Singaporean population Digital literacy: not as accessible for certain populations (e.g., older vs. younger populations)
Chin et al., 2019 [34]	Healthy	Supervised a team which were estimating lactose values from the ASA24-reported foods and manual look up of foods in NDSR	-	Ranking classifier accuracy: combined XGB model > LASSO and Ridge classifier	Successful estimations of NCC-exclusive nutrient (lactose) for food reported in ASA24	Small dataset used for training and testing: underrepresentation of certain food groups, mixed dishes or recipes
Chotwanvirat et al., 2024 [35]	Other	Common practices of Thai Dietitians were applied to arrange ingredients and prepare ready to eat foods	D: Newer system outperformed v4-based system in estimations of fat and protein. No significant difference in measurements of carbohydrates.	-	Eases and reduces user burden in tracking dietary intake	Labour-intensive and costly to obtain a large and detailed image dataset for effective training for a deep learning system
Cohen et al., 2023 [36]	Other	NS	-	-	Both types of models (2D or 3D) are practical for implementation in the real-world setting Privacy preservation	Limitations with detecting occlusion; the amount of liquid ingested could not be determined
Davis et al., 2020 [37]	Weight management—overweight, obese	Gathered a list of frequently asked questions from previous diet and exercise trials	D: High levels of dietary compliance: participants achieved recommended dietary intakes 67% of the time. High levels of compliance to key MED diet food groups.		Positive user experience promotes user engagement	Generalisability of study findings: sample was 68% females and 32% males (different levels of willingness to engage)
De Marchi et al., 2022 [38]	Neurology—amyotrophic lateral Sclerosis	Conduced assessment with multidisciplinary team. Designed a personalised, flexible, normo-caloric dietary plan	A: Weight stabilisation within initial increase for group exposed to the Chabot webapp. Prevention of further weight loss in amyotrophic lateral sclerosis patients. PA: Low rate of drop-out in chatbot use.	-	Potential feasible option for various patient populations Easily integrated into daily patient routines through collection of nutritional data via a conversation-style system	
Farooq et al., 2016 [39]	Healthy—no medical conditions that would impact chewing	NS	-	-	Automatic, objective, and accurate monitoring of self-reported food intake that overcomes the limitation of traditional dietary intake monitoring methods	Testing not conducted for the device’s ability to detect intake of beverages and liquid-based foods Limited variety of foods tested: conducted under laboratory-controlled conditions
Fernandes et al., 2023 [40]	Weight management—obesity	Evaluated whether explanations produced by the tools were understandable and reliable for use	A: Model predicts weight loss as early as 2 weeks into the intervention	Accuracy: 81%; specificity: 86%; sensitivity: 69%	Use for early prediction of success in a weight loss program to allow for timely modifications to enhance effectiveness of intervention	-
Fontana et al., 2014 [41]	Healthy—no conditions that would impact normal food intake	NS	D: Correctly detected major meals (breakfast, lunch, and dinner), 27 episodes of snacking were incorrectly predicted.	Accuracy: 89.9%	Efficient and reliable collection of self-reported dietary intake Long battery life Device is convenient to wear as a pendant on a lanyard. Potential behaviour modification tool for targeting food intake patterns (for weight management and other eating disorders)	Development of the device assumed that eating occasions would be a sedentary task. Does not account for eating whilst active (e.g., walking) Potential reporting error (self-reporting aspect of technology) Assumption: jaw motions in relation to liquid intake would resemble eating intake
Fontana et al., 2012 [42]	Healthy	NS	-	Accuracy: 90.52% Time resolution: 15 s	Automated detection of either food intake or no food intake	-
Garcia et al., 2019. [43]	Healthy	Served on a panel of human expert validators	D: Behavioural changes towards nutrition and food—participants become more equipped to altering eating habits. Assisted users with building meal preparation ideas and skills for cooking via step-by-step recipes enabling more consistent dietary change and greater awareness of health. Positive feedback on app functionality and ability to generate meal plan with optimal macronutrient distribution with respect to daily calorie intake. PA: Users expressed willingness and trust with interacting with the meal recommendation system.	AQEL (nutrition app quality evaluation tool)	Accessible on multiple smart devices Assists with behaviour change and building diet-related knowledge Development self-efficacy and health awareness to support more consistent dietary changes	-
Gonzalez-Flo et al., 2023 [44]	T2DM	NS	B: Regulation of glycaemic control D: Evolutionary algorithm was able to provide tailored assisted regulation of glycaemic control by establish patterns of food intake and insulin doses (basal and postprandial).	-	Personalised advice can be provided in accordance with patient characteristics to enhance chronic disease management	-
Hansel et al., 2017 [45]	T2DM obesity	NS	A: Improvement in cardiometabolic and anthropometric risk factors, weight loss, and reduction in waist circumference. B: Improved glycaemic control and aerobic fitness. No significant differences in physiological biomarkers between the two arms at 4 months. No significant differences in terms of change in blood pressure, plasma lipids, aminotransferases, gamma glutamyl aminotransferase, uric acid, fasting glucose, VO2 max, or hs-CRP were observed between the two arms at 4 months. C: No significant difference in blood pressure. D: Improvements in dietary habits and intake of healthier foods (e.g., lipids, saturated fats, sodium and empty calories).	-	Reach: potential to be used in remote or rural population to provide dietetic service and is a cost-effective option	Internet access required
Hauptmann et al., 2022 [46]	Weight management—overweight	NS	A: Minimal changes to physique of patients. D: Positive impact on nutritional behaviour evidence by optimal intake of nutrients. Reporting of changes to healthier eating behaviours (e.g., eating more fruits or smaller portion sizes). PA: Greater engagement with visual feedback screens, which led to change towards healthy behaviours compared the recommender features of the app.	-	Easy to access on mobile device Visual reflective feedback facilitating better engagement for behaviour change Interviews with users indicated that crucial factors to consider when designing apps are as follows: stress, tracking efforts, social occasions	Nutrition technical jargon within the app design—more difficult to use Low recommendation diversity: only basic recipe recommendations (due to open data source) Greater consideration of personalisation and user context required
Heremans et al., 2020 [47]	Dyspepsia	NS	**-**	Accuracy: Experiment 1: 0.93 Experiment 2: 0.83	Feasible method for monitoring food intake using other measures (i.e., electrocardiogram heart rate variations) for management of chronic health conditions (e.g., obesity, diabetes)	Generalisability of findings: testing only conducted in laboratory settings Does not consider different food intake patterns (e.g., snacking, fasting) and type of food consumed
Hezarjaribi et al., 2018 [48]	Healthy	NS	**-**	Accuracy: 80.6%	Ease of use and convenient: accessed on smartphone and reduces user burden to self-record information	Not as user-friendly for non-native speakers Access to smartphone is required
Hieronimus et al., 2024 [49]	Other	Data was compared to dietary reference intakes calculated using the USDA DRI calculator used by health professionals	D: Daily meal plans generated contained meals and snacks. AI was able to differentiate between health and unhealthy options. ChatGPT provided food items compliant with specific diets. Bard generated incorrect diet plans. Some micronutrients did not meet recommended values for ChatGPT and Bard. ChatGPT had small portion sizes.	-	Useful for brainstorming general food or meal inspiration	Results from the chatbots may differ depending on phrasing on input Model was not tested on more vulnerable populations (children, pregnant, or lactating women), which have differing nutritional requirements
Holmes et al., 2019 [50]	Weight management—overweight	NS	A and D: Progress tracking was determined based on the most important features. Self-reporting aspects should retain minimal interactions to maintain convenience.	-	Encourages user engagement Seamless integration into everyday living	Use of generic messages provided by the system were less preferred by users
Hossain et al., 2020 [51]	Healthy—no chewing conditions	NS	-	Average precision: 82 ± 3% Average F-score: 74 ± 2%	More detailed and accurate information provided than self-report and sensor-based methods	Additional computational costs (e.g., larger battery) and larger storage requirements required for continuous image capture, which can be impractical for everyday living conditions (e.g., user comfort)
Hsiao et al., 2011 [52]	Healthy	NS	D: The flexible personalised meal service selection system was able to deliver a tailored meal plan tailored to the user’s requirements. Young adults rely on the internet as the main source for self-learning of healthcare information (e.g., diet). Perceived usefulness, peer influence, social network, and trust in professionals have a positive impact that drives engagement with the system.		Tailored to engagement needs of young adults to deliver relevant and accurate diet-related information to the public Target behavioural change aspects of dietary change through utilising the decomposed theory of planned behaviour as the research model.	The flexible personalised meal service selection system has not been validated and tested yet
Hsu et al., 2011 [53]	Healthy	Development of database of menus, retrieval of participant data from dietary records, Adjustments of personalised dietary recommendations and nutrition information Evaluation process	D: The recommended menus generated by the fuzzy decision model are reliable and valid. Dietary analysis and recommendations can be used a decision-making tool for dietitians.	-	Tailored interface for both different types of users: public users and dietitian use	-
Jactel et al., 2023 [54]	IBS, Crohn’s disease, ulcerative colitis	Evaluation of nutritional adequacy based on the participant’s list of trigger foods	C: 67.7% achieved total symptomatic resolution by the end of the intervention; 89% reported improves to QoL. Engagement in the program observed improved symptoms and symptomatic resolution in patients with IBS and comorbid IBS/IBD. Significant improvements were reported by 81% at midpoint and persisted for 70% by the end of the study. Measured on IBS symptom severity score and Patient Simple Clinical Colitis Activity Index. PA: Adherence: 89%; retention: 95%.	Patient satisfaction: 92%	Measurement of primary outcome using clinically validated symptom severity scores rather than self-reported symptoms Precision nutritional management for IBS and comorbid IBS/IBS populations	Limited generalisability of findings to other populations and health conditions
Ji et al., 2020 [55]	Healthy	Evaluation of food images and nutrition analysis. Application of the Dietitians of Canada Handy Guide to Servings Sizes	D: Caution should be taken when interpreting results when considering predictions for nutrient content (e.g., carbohydrates, energy, protein, %fat, saturated fatty acids and iron). Significant difference between participant and dietitian data from the app with 12/22 nutrients. Use of data from the app for nutrition assessment would require dietitian review. More effective and stronger levels of validity vs. 3-day food diary when analysing food intake on groups or population levels compared to individuals. PA: High attrition rates (26.5%) due to time or interest in using app.	System usability scale preference for Keenoa app: 34.2%; preference for 3-day food diary: 9.6%	Cost-effective and time-efficient Reduced manual data entry by users and dietitians as it linked to the Canadian Nutrient File (2015), therefore reducing possibility of errors. Strong generalisability of findings (based on sample size of current study) App can be accessed remotely, benefiting occasions of eating out (no need to carry physical food diary)	High motivation of required by user to capture food intake Requires dietetic verification of patient data entries prior to processing with nutrition analysis and assessment Limited database—cultural food content which limits extent of food selection
Jin et al., 2024 [56]	End stage renal disease haemodialysis	NS Note: Nephrologists were consulted to assess GPT’s accuracy	B: Significant reduction in patient’s serum potassium levels when individuals followed the system’s recommendations vs. conventional dietary guidance (4.57 +/− 0.76 mmol/L vs. 4.84 +/− 0.94 mmol/L). Not statistically different for pH levels. D: Dietary education provided by GPT tool significantly reduced the proportion of haemodialysis patients with hyperkalaemia from 39.8% to 25%.	Accuracy of outputs compared to the Mayo Clinic Renal Diet Handbook: Overall accuracy: 65% Accuracy for higher potassium foods: 85% Accuracy for lower potassium foods: 48%	Viable and cost-effective option Contribution to the provision of more personalised efficient dietary and medical care that potentially enhances patient outcomes Potential use as an adjunct to support conventional dietary interventions	Diversity and nutritional content of foods from different cultures may not be full represented, potentially impacting model’s performance in other clinical situations Current model utilises a limited database, resulting in poorer degrees of precision
Karakan et al., 2022 [57]	IBS (M-subtype)	Designing and administrating diet based on AI recommended micronutrient profiles. Monitoring diet adherence	B: Increase in Faecal bacterium genus, *Bacteroides*, putatively probiotic genus, and *Propionibacterium* populations in gut microbiome samples in the personalised nutrition group. C: Change in categorisation of IBS-SSS from severe to moderate was observed only in the intervention group. Change over time of IBS-SSS from pre- to post-intervention was significantly greater in the personalised nutrition group. D: AI-based dietary modification targeting microbiome modulation resulted in significant improvements in symptoms of patients with IBS-M.	Accuracy: 91%	Personalised nutrition based on an individual’s need, progress, and medical condition	-
Karmakar et al., 2023 [58]	Healthy	NS	D: Multimodal MS-TCN performs well in recognising common eating and drinking behaviours.	Accuracy: 83.03% F1 scores were noted as reasonable for each individual class tested	Reduced patient burden: used to assist in dietary monitoring (particularly children and elderly populations)	-
Khan et al., 2022 [59]	Healthy	NS	D: System can distinguish between drinking instance, solid food, and other activities. Observed weaker performance in identification of instance of fluid consumption (e.g., drinking water).	Averaged class-wise precision: 84.65%; average recall: 80.81%; average F-measure: 82.61%; average accuracy: 92.65%	Automatic—reduced user burden, affordable solution/technology Provides user comfort, low power consumption for day-long use Potential use in cross-checking with dietary recall to capture all data for a more accurate assessment	Weaker model performance for fluid consumption Designed for measurement of dietary habits and does not provide estimations on caloric intake
Kiriakedis et al., 2024 [60]	Nephrolithiasis	Evaluation of ChatGPT’s responses for accuracy, completeness and appropriateness	B: System had poorer performance when detecting and responding to urine test abnormalities, especially in calcium and citrate levels. Unable to address 30% of abnormalities impacted quality of recommendations offered. Good performance in identification of biomarkers within normal range parameters. D: System could generate personalised recommendations. Performed well in terms of accuracy, completeness, and appropriates for advising hydration.	Likert Scales: Accuracy (5.2/6), completeness (2.4/3), appropriateness (2.6/3)	Reducing administrative burden whilst enhancing efficiency and effectiveness of dietitians’ work Potential for supportive tool for dietary management of nephrolithiasis through providing recommendations Foundations for improved accessibility and quality of personalised patient care	Inconsistencies in responses can result in misinformation. Requires some nutrition knowledge to assess responses Correct responses were reliant on prompts embedded with specific guiding medical terminology ChatGPT’s model is constantly evolving results from this study only reflect performance at one point in time. Did not compare results from another point in time Does not cater to different user reading levels
Kirk D. et al., 2023 [61]	Healthy	Gathered the most asked nutrition questions with corresponding dietitian answers. Graded ChatGPT responses for scientific accuracy, applicability, and comprehensibility	D: Capability of answering commonly asked nutrition questions ChatGPT scored higher in comparison to dietitians in areas of scientific correctness (5/8), accountability (4/8), and comprehensibility (5/8).	Overall grading: ChatGPT responses were higher for 5/8 questions	Empower patients to become more involved and aware of their own health. Free service and readily accessible at any time. Quick responses to questions	Responses provided by ChatGPT do not have indications of confidence Users require some background knowledge to assess responses provided Does not account other complex factors that influence an individual’s state of health.
Kobayashi et al., 2024 [62]	Dyslipidaemia, diabetes	NS	D: Diabetes case: Recommended and non-recommended recipes are highly appropriate for the purpose of health improvement. Dyslipidaemia case: There was no significant difference in the top and bottom 5 recommended recipes. This indicates that the inference system may not have worked as well for this medical condition. Recommendations made by the system were lacking in variety and intrigue.	Precision: >0.99	Used in dietary interventions to provide a larger variety of actionable ideas for patients	Further refinement required to balance variety of recipe output for conditions that have dietary restrictions
Krishnakumar et al., 2021 [63]	T2DM	NS	A: Improvements in weight management of diabetes: weight reduction of 1.32 kg and BMI reduction by 0.47 kg/m^2^. B: Improvements in management of diabetes. Incremental reduction in blood glucose biochemistry markers (HbA1c, FBG, and PPBG); 63.7% (65/102) patients had improved HbA1c levels after intervention. PA: Greater levels of engagement and retention rate. Over 16 weeks: average duration of time spent with the following: Personal health coach was 106 min. AI powered chatbot: 88 min.	-	Effective in assisting the documentation and monitoring of changes over time (e.g., HbA1c levels) and in improving glycaemic control	
Kwon et al., 2024 [64]	NAFLD	Consultations with participants on goal setting and evaluation. Provided monthly feedback from dietary intake recorded in app	A: Significant improvements in physiological outcomes (weight loss and BMI) for 6 months B: Significant improvements in liver panel tests (AST, ALT, and GGT) for 6 months. C: Significant improvements in psychological outcomes (self-management, fatigue, depression, and QoL) for 6 months. D: Greater engagement observed higher levels of self-management and knowledge. PA: Mean compliance with respect to using app: 3 months: 82.6%; 6 months: 79.8%.	-	Encourages individuals to develop self-management skills, facilitating more long-term beneficial health change behaviours	Limited to Android phone users
Lázaro et al., 2010 [65]	Aging population (preventing malnutrition)	NS	-	System was able to differentiate the level of complexity of the randomly selected recipes at a higher percentage: 78%	Allows nutritional management to be more effective through influencing the user and automated and seamless monitoring; enables elderly population to remain autonomous Provides a nutrition estimate based on nutritional habits of the user	Preference for free apps Generalisability of results; prior testing was only conducted in simulation lab conditions
Lee et al., 2024 [66]	Other	NS	D: Utilisation of the platform enhances accessibility and engagement, allowing individuals to obtain nutrition guidance in a seamless manner. Information gathered by the bot was able to synchronise data from external databases and user-reported data to allow for the provision of continuous care.	-	Addresses the challenges of traditional dietary assessment AI-driven comprehensive nutrition care and management informed by monitoring nutritional intake using user-reported data with precision analytics	-
Lee et al., 2017 [67]	Healthy	NS	-	F1 score: 0.96 Overall accuracy: approximately 90% Average accuracy: 99% (identification of plate sections MLP) Average accuracy: 90.40% (estimation of food portion and weight)	User friendly diet monitoring system that assists with promoting health eating habits	-
Lee et al., 2020 [68]	Healthy	NS	D: Ultrasound modality resulted in lower recognition rate for certain foods that had similarity of softness and crunchiness.	Recognition performance for artificially added noise: 90.13% Recognition performance for noisy environments: 89.67%	-	Limited number of foods recognised. Improvements required for a more compact size and improved wearing comfort Certain food types are more suited for utilisation; audio recognition rather than ultrasound
Lee et al., 2023 [69]	T2DM	Sending personalised nutrition intervention messages	A: Utilisation of the platform resulted in weight loss. B: Better management of glycaemia levels with significant reductions in HbA1c levels (baseline to 24 and 48 weeks).	-	Potential efficacious tool for building self-management of T2DM in relation to dietary management	Limited generalisability of results: testing conducted on a specific demographic (no utilisation of insulin, glucagon-like peptide 1 agonist, or anti- obesity medication in participants; HbA1c 7.0–8.5% and BMI 23 mg/m^2^)
Li et al., 2024 [70]	Other	Dietetic students assessed nutrition-related apps for their suitability to be applied into the NCP	D: Noom was the highest scoring app. MyFitnessPal and Fastic had the highest degree of accuracy. Generally, energy content was overestimated for Western meals and underestimated for Asian meals.	Accuracy: MyFitnessPal: 97% Fastic: 92%	Provides an estimation of food components and energy estimation Can be used as an adjunction to provide professional guidance, evidence-based recommendations, and counselling that complements the behaviour change techniques offered in apps	Collaborations with dietitians are essential to identify reputable apps and improve credibility Comparative validity limited by food database underpinning the app. Some apps require payment or additional training for users Food image estimation is limited by constraints of the image taken (e.g., lighting, angle, background) Digital and technological literacy
Liao et al., 2024 [71]	Other	Assessed quality of ChatGPT’s dietary advice	D: Responses by ChatGPT scored highly in readability but lacked understandability, practicality, and completeness. Dietitians commented that the responses often lacked thoroughness and rigour, which can potentially lead to misunderstanding.	Accuracy: 84.38% Objective Nutrition Literacy Test (NL): 7.50% to 37.56% (suboptimal performance)	Enhance the efficiency and reach of nutritional counselling (especially in settings with high client volumes and limited resources) Valuable tool for nutrition education as information is quick and accessible	Require further development and fine-tuning prior to clinical use Use should be approached with caution as it requires professional judgement or nutrition-related knowledge to assess responses for presence of misinformation Limited generalisability of findings: study focuses on college students
Lin et al., 2020 [72]	Healthy	NS	D: System can recognise eating motions and provide estimations in chewing and swallowing incidents.	Accuracy: 95% Error: 10%	Automated dietary monitoring as opposed to manual food logging	Measurements by the device can be influenced by surrounding sounds
Liu et al., 2024 [73]	Healthy	NS	-	F1 scores: Four food-related intake activities: 86.4% Classification of seven types of foods: 64.2%	Automated dietary monitoring via tracking of the bites taken to estimate food intake. Could be used as a food intake diary	User discomfort with long periods of use: direct contact with skin required for continuous impedance monitoring
Lozano et al., 2023 [74]	Weight management – overweight	NS	D: Errors in automated energy estimates were relatively high prior to adjustments for beverage-based food items. After adjusting for beverage, the remainder of error was lower (16%). Most energy estimates were driven by the grain products.	Matches for food items: Exact: 46% (118/255); 41% (105/255) were a fair match, and 13% (13/255) were intrusions.	Comparable rates of error in semiautomated energy estimates when compared to traditional dietary assessment methods Presence of barcode scanning feature could be efficient with respect to the identification and quantification of packaged foods	
Maher et al., 2020 [75]	Weight management—overweight, obese	NS	B: Average loss of weight of 1.3 kg and 2.1 cm from waist circumference. C: No significant changes in blood pressure. D: Adherence to the MED diet increased during the intervention. PA: Adherence to the MED diet increased during the intervention; 70% engagement with Paola.	-	Enables users to converse in a conversational manner with Paola Use of objective health measures and intention to treat analysis Can be used to support dietary education on the MED diet and assist with monitoring and evaluation	Digital and technological literacy: barrier making it difficult to engage in the program
Marashi-Hosseini et al., 2023 [76]	Metabolic syndrome	Commented on results from questionnaire used to establish recommended macronutrient requirements for health condition. Development of gold standard to compare algorithm’s output	D: No significant differences between the diet set by nutrition professionals and the diet recommended by the fuzzy logic model. Proposed system enhanced the reliability, speed, and accuracy of the decision-making of dietitians in setting an optimal diet for patients with multiple chronic conditions	Accuracy: approx. 97% (suitable performance)	Support tool for increasing dietitians’ confidence, precision, and efficiency when delivering interventional diets for patients via calculating the macronutrient requirements for daily consumption, weight, and chronic conditions Improved quality of care as multiple sources of information are integrated to inform the decision-making process for dietitians Enhances reach of dietetic services	-
Martino et al., 2021 [77]	Aging Population (prediction of malnutrition risk)	NS	D: High levels of accuracy and recall in detection of individual nutritional status through combining data from nutritional intake, dietary habits, and body composition data (models tested: LR with LASSO regularisation, RF, AB, RUS Boost).	Best-performing ML models for malnutrition risk: Accuracy: 94% Recall values: 92%	Cost-effective approach	System development using a small database. Large database preferred for future re-iterations
Mertes et al., 2020 [78]	Healthy—able to self-feed	NS	D: Current model can produce a higher recall and the lowest error. Algorithm is more prone to under-reporting actual food intake.	Precision and Recall: 0.78	Reduced dietary recording burden and potential for increased food tracking adherence for longer periods of time and minimised attrition bias Improvements in food estimates (e.g., whether meals have been finished); reduces burden or workload of health professionals when gathering patient food intake data More accurate intake estimation in nursing homes or long-term care residential facilities	Algorithm was programmed for the use of chopsticks and was not programmed for the use of another cutlery. Prone to errors in bite detection when there is user interaction with the plate: rearrangement of foods, leaning on plate when eating or increased eating speeds
Moyen et al., 2022 [79]	T1DM, T2DM	Manual adjustment of incorrect food intake entries into the app by participants	D: Acceptable agreement between both tools without bias. Moderate-to-strong relative validity compared to ASA24 in terms of macro- and micronutrient intakes for healthy and diabetic adult populations (fibre and iron were reported to be higher and sodium was lower when using Keenoa rather than ASA24). No significant differences in estimated energy intake between tool with similar rates of underreporting and no overreporting observed.	Mean usability score (perceived ease of use): Keenoa: 77% ASA-24: 53% Preference for Keenoa over ASA-24: 74.8%	Increased user acceptance due to reduced invasiveness or burden on user New and alternative tools that facilitate the work of the dietitian; improvements in food-tracking adherence for longer periods, with reductions in attrition bias Larger database compared to prototype 0.3.7 version [55]—more cultural food content from other national databases to facilitate patient data entry Ease of use integrated functions (e.g., ability to copy over previous meals) may increase adherence, leading to better estimations of food intake	Does not prompt users with questions (e.g., addition of salt) that may lead to under-reporting Does not include supplements—needs to be manually add by HCP
Naja et al., 2024. [80]	Metabolic syndrome	Evaluation of ChatGPT’s responses in accordance with Nutrition Care Manual with focus on accuracy, clarity, coherence, and practicality	D: Outputs for dietary management of T2DM and metabolic Syndrome were often partially/incomplete or did not align with NCP recommendations. Impacts patient care and accuracy of output. Dietary advice did account for energy balance/intake modification. Assessment of nutritional status was incomplete (did not account for current intake of macronutrients and anthropometric markers). PES statements and appropriate diagnostic terminology were not utilised.	-	For patients: Available resource, easily accessible, interface had excellent levels of clarification, coherence, and practicality. For dietitians: Provides a quick dietetic second opinion or brainstorms intervention ideas	Limitations in being able to provide a holistic approach to interventions. Does not account for multidisciplinary care (not reflective of current healthcare practices) Professional oversight for nutrition-related recommendations required: Requires dietitians to correctly define the issues and utilise the appropriate input prompt for an appropriate and accurate output Additional education for patients is essential on how use the chatbot in a correct and safe manner to aid dietary monitoring and intervention Inconsistencies and variations in generated responses over time with model optimisation and training and with different prompt phrasings Credibility of ChatGPT is inconclusive due to lack of mentioned references
Nakaoka et al., 2021 [81]	Other	NS	-	-	Does not require participants to wear special devices, which may interfere with eating behaviours Real-time feedback to users, which can be used as a reinforcement and encouragement for healthy and mindful eating	-
Nakata et al., 2022 [82]	Weight management—overweight, obese	NS	A: Significant body weight decreases between intervention and control groups. B: No changes in blood biochemistry measures. D: No differences between two groups for energy. Group that used dietary logs on the app had a decrease in energy intake between the initial and final 2 weeks (−152.3 ± 304.0 kcal).	-	Easier recording method Efficient provision of feedback to users Free access, which can be used in healthcare settings Significant body weight decreases between intervention and control groups. No significant change in blood biochemistry measures	-
Niszczota et al., 2023 [83]	Food allergies	Generation of prompts and assessment of responses by ChatGPT	D: Potential for outputs with errors that involve inaccuracies in portions or calories of food, meals, or diets. In some cases, there can be severe health consequences: for instance, including almond milk in nut-free diet. Most menus correctly excluded allergens of interest (52 of 56 prompts). Can be used for meal formulation using basic recommendations. Displays cautionary safety labels to raise awareness in users. Miscalculation of energy values for food, meal, or complete menus. Portion sizes and quantities recommended were very specific and impractical. Repetition of products and meals were common, which makes outputs monotonous and challenging.	-	-	Requires further fine-tuning in collaboration with HCPs to review ChatGPT outputs and identify misleading information
Ocay et al., 2017 [84]	Other	NS	D: Nutritional awareness of respondents was low. After interaction with app, there were high user satisfaction ratings and acceptance (user interface, ease of use, nutritional content estimation, basal metabolic rate calculation, reliability of food recognition, food intake monitoring, and improved diet awareness). Potential to improve user awareness through tracking and dietary monitoring.	Likert scale: User acceptability: 4.43/5 Reliable photo recognition: 5/5	Convenience with dietary monitoring through using photo recognition nutrient estimation Increases user’s awareness of nutritional content and food intake	-
Oh et al., 2022 [85]	T2DM hypertension	NS	A: No significant differences in body weight, BMI, and body composition between two groups. Any changes in body fat mass were attributed to medicine intake. B: No significant differences HbA1c between the two groups Any changes in HbA1c levels were attributed to an increase in medicine intake. C: No significant difference for blood pressure between both groups. D: Significant low input rate for food intake data with attrition. The notable difficult functions for input of food intake were recording food intake and locating food items from the provided list. Medication input systems experience greater input adherence and were more appealing to users.	Likert Scale: Helpful: 3.4/5 Easy: 2.9/5 Well-functioning: 3.1/5	Potential to encourage positive behavioural changes and allow for the development of better self-management skills and adherence Cost-effective tool to assist management of chronic health conditions and reduce costs associated with healthcare for aging populations	Inconclusive impacts of long-term efficacy of integrated mHealth Digital literacy and difficulties adapting to new technologies; apps and medical devices may be less accessible for aging populations.
Papapanagiotou et al., 2017 [86]	Healthy	NS	D: Utilisation of a combination of signals from all sensors yield improved results compared to results from individual signals (e.g., audio only).	Accuracy: 0.938 Precision: 0.794 Recall: 0.807 F1 score: 0.761	Potential for use in dietary monitoring apps: low sampling rate and computational requirements	Future reiterations and fine tuning required to cater to user comfort and integration with mobile devices Unable to detect fluid intake
Papapanagiotou et al., 2021 [87]	Healthy	NS	-	Mean absolute error: <1 (3 out of 4 of the food types)	-	Technology was built from smaller datasets (not under free-living conditions) and there are requirements for annotations of individual chewing instances Training for correct fitting is also required to achieve optimal higher amplification of chewing signals
Papastratis et al., 2024 [88]	Other	NS	D: Meal plans generated were appropriate in terms of energy and nutritional requirements. High levels of accuracy and validated on 3000 virtual user profiles and 1000 real person profiles with 91,000 meal plans generated from the Protein NAP database (large open-source collection of international meals).	Macronutrient accuracies: Average: 87%; fat: 84.04%; SFA: 89.55%; protein: 86.78%; carbohydrates: 83.18%	Used to develop personalised nutrition and diet planning: weekly meal plans to achieve dietary goals for users with health conditions (CVD and T2DM) without the constraints of a small meal database. Speed and simplicity; can provide generalised information acquired from nutritional guidelines. System (ChatGPT and deep generative model) able to account more complex cases with different backgrounds and medical conditions System (ChatGPT and deep generative model) could be potentially leveraged to provide personalised nutritional advice for prevention or management of symptoms	Proposed system has yet to be incorporated with user-orientated technology. It is designed to be incorporated into apps, such as healthcare apps, fitness apps, or dietary consulting services, to be used in real-life applications
Papathanail et al., 2023 [89]	Healthy	Carried out 24 h dietary recalls on participants	D: New system has comparable energy and macronutrient estimations performance in comparison to dietitian’s 24 h recall. The re-iteration of the system only requires one image instead of two images. The newer method does not exhibit a statistically significant difference in mean absolute percentage error compared to the two-image system. PA: Discrepancies arose for user compliance with recording meals, omissions, and neglecting the use of reference cards.	Macronutrient estimation errors (system vs. 24 h recall): CHO: 31.27% Protein: 39.17% Fat: 43.24% Misestimation of the new method: Energy: 2.16%; CHO: 0.34%; protein: 3.46%; fat: 0.02% User satisfaction: Tracking food intake: 52.4%; neural: 45.2% recommend to others: 71.4%	Complete automatic system that allows for energy estimation and macronutrient profiles from a single meal image. Comparable performance to the two-image system More user friendly with decreased user burden for two images Potential to facilitate dietary monitoring with reduced costs associated with dietary assessment Raises individual awareness	Refinement required for system to interpret closely viewed items, image acquisition, and user compliance Current testing is on data that is not reflective of real-world environments. Future trials are focused on incorporating the system into smartphones to evaluate performance, accuracy, and validity. Improvement required to improve user compliance.
Papathanail et al., 2022 [90]	Other	Group identified 31 categories of food items	D: Validation of a fully automatic food and drink recognition system that requires a single image. System able to estimate serving sizes and calculate user’s MDA score.	Mean absolute error of MDA score (system vs. dietitian): 3.5% (non-significant) Mean average precision: 61.8% Positive response from users: 83%	Automatic food recognition system that can calculate MDA scores and provide tailored feedback to the user	Lack of correlational or causal evidence for impacts of system on anthropometric markers and patient adherence to the MED diet Refinement in app design required to meet the needs of users. Needs further input/consult with HCPs and users with respect to perspectives on usability and acceptance
Ponzo et al., 2024 [91]	Dyslipidaemia, hypertension, T2DM, obesity, NAFLD, CKD, sarcopenia	Questions and prompts generated for input were formulated by medical doctors and registered dietitians	D: Reasonable level of accuracy with general dietary advice for non-communicable diseases. Able to provide practical examples of foods to include or exclude from the diet. For some cases, incomplete recommendations (few guidelines missing) were provided for T2DM, obesity, dyslipidaemia, NAFLD, and CKD. Unable to provide suitable guidance if multiple conditions coexisted.	Appropriateness: 55.5–73.3%	Clear, easy, and simple avenue for people to obtain nutrition advice and assistance Provision of broad nutrition recommendations with practical examples of food Flexible with round-the-clock accessibility as it could be easily accessed across devices Extends reach of nutritional advice and dietetic services across rural and underserved areas Cost-efficient	Unable to provide recommendations in more complex cases, e.g., multiple health conditions present, leading to contradictory or inappropriate advice Dietitian supervision required with use due to partially incomplete responses and information contradicting guidelines (should not replace a nutrition professional or expert) Absence of warning and notification for food allergies for recommended food
Qarajeh et al., 2023 [92]	Other	NS	D: Emerging AI models manifest a diverse range of accuracy in discerning potassium and phosphorus content in foods suitable for CKD patient. ChatGPT 4 performed better in classification of high- and low-potassium and -phosphorous foods compared to ChatGPT3.5	Accuracy of model compared to Mayo Clinic Renal Diet Handbook: ChatGPT 4: 81% for potassium foods; 77% accuracy for phosphorous foods ChatGPT 3:66% for potassium foods; 85% accuracy for phosphorous foods Bing Chat: 81% for potassium foods; 89% for phosphorous foods Bard AI: 79% for potassium foods; 100% for phosphorous foods	Can be used by dietitians to completement personalised dietary planning for CKD patients Supplementary tool to enhance nutrition education	Refinements in AI models required for optimal utility and applications in dietetic practice (e.g., user interface). Comparison of AI model performance only limited to the Mayo Clinic’s Renal Diet Handbook Requirement of nutrition expert/dietitian oversight recommended
Rafferty et al., 2021 [93]	IBS	Co-designed software with software engineers	C: Improvements in quality-of-life outcomes and bowel-related and habit-related symptoms (2-fold improvement), with 43% of participants in the intervention group no longer meeting the criteria for IBS (Rome IV criteria). QoL improvement scores were correlated with improvements in IBS symptoms scores. D: Both app and control groups had improvements in knowledge and adherence.	Reduction in total IBS symptom severity score: 24% greater for the APP group vs. CON group	AI-integrated app developed with a focus on IBS and low-FODMAP diet with the aims of improving quality of life and disease outcomes Provided evidence supported education and real-time feedback on food choices via a personalised match rating (using the traffic light system) to support user adherence to difficult diets (e.g., low-FODMAP diet). Easily accessible	Inconclusive impact on anthropometric measures.
Salloum et al., 2018 [94]	Healthy	Evaluation of participant data and findings	A: Body fat percentage and weight recommendations produced by the system: 88.7% agreement with nutrition experts. C: Automation Health State Assessor system: rated > 3.5/4 by 31.2% of nutrition expert ratings. D: Meal plan generator system: experts agree with meal plan suggestions provided, including nutrients of focus and assigning food items. Improvements required for food variability.	-	Potential to aid in gathering and reaching nutritional diagnosis Providing personalised interventions to increase patient adherence and engagement	-
Samaan et al., 2023. [95]	IBD	Evaluation of ChatGPT responses	D: Comprehensive response to 62.5% of question relating to nutrition and diet needs for surgery, 92.3% questions relating to tube feeding and parenteral nutrition, 64.7% general diet questions, 50% of questions relating to diet for reducing symptoms/inflammation, and 81.8% of questions relating to micronutrients/supplementation needs.	Correct responses: 83% Comprehensive responses: 69% Incorrect/contradicts guidelines responses: 17% High reproducibility in accuracy: 92%	Potential to be used as a complementary source of IBS nutrition information or education for IBD patients Extends the reach of dietetic information or service in locations where healthcare professionals are not readily available Relevant and accessible for young adult patient populations with high computer literacy Empowers patients, providing them with a greater sense of patient autonomy	Further improvement on performance required: requires additional training with medical databases and the literature Lacks readiness for clinical use: can provide inaccurate, outdated information; unable to comprehensively assess individual health status, address behavioural needs, or monitor patient progress
Sano et al., 2015 [96]	Healthy	Evaluation of participant data and findings	D: ~ 40% of the participants ate more balanced meals and increased vegetable intake; ~20% participants report changes to poor dietary habits, i.e., avoiding snacking/eating occasions prior to sleep based on the system’s recommendations. >50% of participants became more aware of their diet, sleep, activity, and stress through tracking behaviours and engaging with surveys; 73% found diet advice was helpful	-	Increasing self-awareness for behaviour change and support patient engagement Provides actionable nutrition-related advice that is readily applicable to the working patient population	-
Schiboni et al., 2018 [97]	Healthy	NS	D: Specific orientation and position of the camera limit privacy-infringing content captured in videos. Eliminates the need to integrate error-prone anonymisation to limit privacy concerns. Model performance suggests this is a feasible method.	Average recall > 90%	Reduces patient burden with dietary recall as it assists with gathering food intake and monitoring patient adherence to interventions	Additional training of models requires accommodations due to the high variability of food items under free-living conditions. This process can be laborious and expensive Potentially, food detection readings can be impacted by user motions, posture, and activities
Sefa-Yeboah et al., 2021 [98]	Weight management—obese	Involved in testing of the program	D: System was also able to generate a personalised nutrition report which includes daily nutritional needs (e.g., macronutrients) and energy intake and offers appropriate recommended meals with high levels of accuracy. Different energy targets were used (1000, 1600, 2000, 2400, 2800, and 3200 kcal) in testing.	-	Software can be deployed on either a mobile or web-based platform Assists in development of self-management strategies	Data utilised by the system does not give an accurate estimation of energy expenditure as it uses predefined levels of physical activity from the Hospital of Special Surgery’s, USA, categories for exercise. This impacts overall output by the system Users are limited to selection options to report food intake—does not have barcode scanning feature
Shamanna et al., 2020 [99]	T2DM	NS	A and B: Continuous AGP monitoring. Adherence for 3 months resulted in a 1.9% decrease in HbA1c, 6.1% weight decrease, 56.9% reduction in HOMA-IR, significant decline in glucose time below range, and reduction in or elimination of T2DM medication use (most patients). C: All 12 patients on insulin discontinued used within 3 months. Most patients on other T2DM medications such as metformin and DPP-4 inhibitors also ceased medications. Patients on liraglutide stopped the medication. D: Helped patients avoid foods that cause blood glucose spikes and replaced them with foods that do not produce glucose spikes. Daily precision medicine guidance based on continuous glucose monitoring; food intake data ML algorithms can provide benefit to T2DM patients.	-	Helped patients avoid foods that cause blood glucose spikes and replace them with foods that do not produce glucose spikes Potential nutrition intervention applicable in outpatient settings, employing home-cooked foods and making the intervention sustainable in the long term	-
Shao et al., 2021 [100]	Healthy	Provided the relative energy per food item in each image used. Conducted structured interview with participants for 24 h dietary recall	D: Outperforms participant estimates and can accurately estimate portion size from a single image. Ability to account for all components of a meal (e.g., oils, dressings, sauces) which would generally be overlooked. Higher levels of accuracy with respect to estimation and applied data augmentation (rotating, cropping, and flipping).	Higher levels of accuracy for food image estimation: MAE: 56.33 calories MAPE: 11.47% (significantly outperforms human estimates by 27.56%)	More accurate recording of estimated food intake as it can consider condiments, dressings, and oils	Additional training of model required as it is trained on a limited dataset
Shoneye et al., 2019 [101]	Healthy	NS	D: Participants in the CHAT intervention reported being shocked and surprised about the feedback on their dietary intake but were receptive to feedback Participants who agreed that dietary feedback made them think about their eating behaviours were more likely to improve their diet during the intervention period (increased vegetable intake by half a serving and decreased intake of energy-dense nutrient-poor foods by half a serving).	-	Effective approach for increasing user awareness, contemplation (important mediator), and motivation around their diet and assists with developing behavioural change Utilisation of text-messaging, which is appealing to younger adults	Study and testing did not include measures of user autonomy and self-regulation
Silva et al., 2022 [102]	Metabolic syndrome	NS	D: This food recommendation system can analyse an individual’s dietary data and provide personalised dietary recommendations. Items eligible for recommendation included whole cereals, tubers and roots, beans and other legumes, oilseeds, fruits, vegetables, white meats and fish, and low-fat dairy products and milk. Utilisation of user-based collaborative filtering (UBCF) vs. item-based collaborative filtering (IBCF). PA: Human and professional support are still required to ensure patient adherence (such as following through with interventions) and to maximise impacts of interventions.	Precision: 88–91% (similar between UBCF and IBCF) Error metrics: Root means square error (RMSE): 1.49 (UBCF) vs. 1.67 (IBCF) Mean square error (MSE): 2.21 (UBCF) vs. 2.78 (IBCF)	Cost-effective and improves outcomes through ability to reach a larger proportion of the target population Ability to provide support for health professionals in delivery of personalised healthcare interventions (e.g., dietary advice) as they can understand the diet characteristics of the individual to promote more better dietary recommendations that can enhance adherence Considers specific sociodemographic and clinical profiles of the user Can be used as a complementary approach—support from healthcare professionals still required to maximise intervention effectiveness and outcomes	-
Sowah et al., 2020 [103]	T1DM, T2DM	Clinical requirements and design analysis of the system was based on discussions with collaborators from the Department of Nutrition and Food Science—the diet type of patients was determined to be an essential approach suitable for the diabetes management system	D: Able to predict and label new food images with high levels of accuracy. The meal recommender model and chatbot were able to provide appropriate recommendations that met the user’s caloric needs and could address user questions via a user-friendly interface.	Accuracy: >95% (food recognition and classification for specific caloric intakes)	Helps with addressing dietary recommendations and medication notifications for diabetics. Flexible and accessible—software can be run on either mobile or web-based apps	-
Sun et al., 2023 [104]	T2DM	Evaluation of ChatGPT response to professional clinical dietitian responses	D: In terms of the ketogenic diet, recommendations mostly followed best practice guidelines, with an overlap rate of 80.7% between dietitian’s recommendations and ChatGPT. Inconsistencies with root vegetables and dry bean food items.	Food recognition model: F1 score: Dino V2: 0.825 Inverse cooking model: 0.477 162/168 favourable reviews of responses from dietitians ChatGPT accuracy: 60.5% Error rates: 64.6% GPT 4.0 accuracy: 74.5% Error rates: 70.6%	Affordable and easily accessible nutrition education and dietetics care and management. Extends reach of essential care to individuals where there are constraints in HCP availability	Systems are less familiar with more culture-specific ingredients and food items Potential for the system to generate different answers for the same prompt Dietitian oversight is still advised in the outputs by the system Additional training for the system is still required
Sun et al., 2015 [105]	Healthy	NS	D: Mostly foods with an irregular shape, small size, or poor-quality images resulted in large errors in food identification.	85/100 food items identified: Presence of Error: 30% Mean absolute relative error (MARE): 16.4% Root mean square error (RMSE): 20.5%	Comfortable for long-term usage: more passive in operation; convenient size and appearance can be personalised	Power management and efficiency: battery supply for the device can only last 4 to 8 h depending on the capacity of the rechargeable battery Design is applicable for real-world use, but there are other factors (e.g., privacy, costs, only small-scale studies conducted so far) that are still considered prior to introduction for real-world use
Thames et al., 2021 [106]	Healthy	Provided portion estimations which were then compared	D: Prediction of caloric and macronutrient values of complex, real-world dishes at an accuracy that outperforms professional nutritionists.	Professional nutritionist absolute error: 41% Non-nutritionist’s absolute error: 53% Calorie per gram prediction with model using data from Nutrition5K database; mean Absolute Error: 16.5%	Robotic automation that utilises depth information for portion estimation, allowing for more efficient data collection	Data collection used for proposed model training is limited to mostly Western-style dishes; reduced exposure to other cuisines
Tunali et al., 2024. [107]	IBS	Face-to-face consultations and delivery of tailored menu plans	C: Microbiome-assisted personalised diet (PD) resulted in significant improvements in IBS-SSS scores (across all subtypes) and IBS-QOL in addressing symptoms (for IBS-C, IBS-D, and IBS-M) (FODMAP diet resulted in improvements in IBS-C and IBS-D). PD led to significant microbiome diversity shifts compared with FODMAP diet. PA: Completion rate of 81%.	-	Personalised and precision nutrition interventions may optimise treatment outcomes and overall health via addressing gut microbiome	-
Turnin et al., 2021 [108]	T2DM	NS	A: Metabolic benefit was correlated with frequency of use of the device, which was reflected in significant weight loss (female participants) and reduction in waist circumference. B: Home telemonitoring and tele education did not significantly improve glycaemic control in T2DM subjects. Slight significant decrease in HbA1c levels (female participants)	-	Reduced healthcare costs—tailored to patient’s needs, medical progress, and availability Allow for more flexibility in patient follow-up for chronic disease management—facilitating frequency of face-to-face consultations with healthcare professionals based on individual needs and availability	-
Valero-Ramon et al., 2019 [109]	Aging population (weight changes and malnutrition screening)	NS	A and D: Models in this study present cases of patients who are malnourished. Indicated malnutrition is related to weight and other factors (e.g., quality and quantity of nutrients and level of activity). These can be used to predict and monitor the risk of malnutrition.	-	PALIA algorithm creates graphical models which can be easily interpreted by healthcare professionals Dynamic models were able to show behaviour and variability over time	Training of this model utilised datasets (short term: 6 months) that were incomplete and, thus, could omit relevant variables that could impact patient outcomes
Vasiloglou et al., 2020 [110]	Other	Manual calculation of MDA scores using the scoring system outlined in the paper Creation and correction of the Oviva database	D: Proposed system and four experienced DTs indicated similar results/predictions in MDA assessment for free-living conditions. Better performance in multi-label food recognition and serving size estimation compared to baseline method (ResNet101 and ImageNet) by 11% for food recognition and 2% for serving size recognition.	RestNet 101: mAP: 0.47; MAPE: 63%; GCN-based: mAP: 0.58 * MAPE 61% (better performance)	Reduced user burden as only one image is required for evaluation of food items Can cater for mixed food items: linking correlations between certain food categories to improve accuracy of output Efficient method of evaluating patient adherence to MD diet	Some reliance on user confidence on portion-size estimation to input some data that cannot be captured by food images (e.g., use of oils, spreads). Training may be required for users to build confidence Focus on generalised MED diet—may not be applicable for specialised subgroups of MED (e.g., vegan)
Walker et al., 2014 [111]	Healthy	NS	-	Correctly identified ingestion sounds: >94% False positive: 9%	Replacing the need for self-reporting/monitoring and limitations associated with it (lower accuracy, lack of awareness/omissions of food intake) Automated and real-time refresh and feedback from user, which allows health professionals to assess and monitor meal lengths and ingestion activity during meals Method for personal record-keeping and essential for the clinical monitoring and management of obesity	Only preliminary studies have been conducted
Wang et al., 2024 [112]	Healthy	NS	-	F1 scores: Eating gestures: 0.896 Drinking gestures: 0.868	Easily applicable in free-living environments due to the integration of non-feeding gestures	Further training required: Data was used to train the system in a static environment, and model can potentially misidentify intake gestures
Wang et al., 2023 [113]	Healthy—endurance athletes	NS	D: Most impactful features included carbohydrate intake rate, age, light sleep, activity levels, which are potential areas for targeted interventions, and personalised strategies to optimise CPS intake. BP neural network model displayed slightly more superior performance than the GBRT model.	-	Delivering tailored and personalised recommendations for carbohydrate protein supplements	System is designed for endurance athlete population
Zhang et al., 2023. [114]	Prediabetes, T2DM	NS		Calorie prediction improvement compared to the following: best CGM model: 10.8%; best image model: 19.5%	Improvements in accuracy in dietary assessment and monitoring, which combined capturing food images (food intake and caloric estimation) with CGM data to support diagnosis	-

Abbreviations: Type 1 diabetes mellitus (T1DM); type 2 diabetes mellitus (T2DM); irritable bowel syndrome (IBS); irritable bowel disease (IBD); non-alcoholic fatty liver disease (NAFLD); chronic kidney disease (CKD); randomised controlled trial (RCT); not specified (NS); body mass index (BMI); haemoglobin A1C (HbA1c); problem, etiology, signs, and symptoms statement (PES Statement); cardiovascular disease (CVD); Mediterranean diet (MED); Mediterranean diet adherence (MDA); fermentable oligosaccharides, disaccharides, monosaccharides, and polyols (FODMAP); quality of life (QoL); healthcare professional (HCP); continuous glucose monitoring (CGM).

**Table 4:** Summary of the primary outcome of AI-integrated technologies included in the scoping review. Table includes each technology, its potential application in the stages of NCP, and types of AI.

Author	Technology	Stages of NCP				Type of AI Technology			
		Ax	Dx	I	M&E	Image/Audio Recognition	Chatbot	Rec. Sys.	AI Subdomain and Specific Techniques
Amft et al., 2007 [20]	Wearable sensor: microphone, EMG	✓	-	-	-	✓	-	-	GA
Chen et al., 2023 [30]	First-bite/first-chew wearable system (glasses paired with wristband, inertial measurement units, machine learning microcontroller unit)	✓	-	-	-	✓	-	-	ML
Chotwanvirat et al., 2024 [35]	Institute of Nutrition, Mahidol University iFood app	✓	-	-	-	✓	-	-	DL
Li et al., 2024 [70]	Various free-to-use and paid/premium versions of nutrition-related apps available on the Australian Apple App Store or Google Play Store	✓	-	-	-	✓	-	-	ML, DL
Lozano et al., 2023 [74]	OpenFit mobile app	✓	-	-	-	✓	-	-	DL—neural network
Papapanagiotou et al., 2017 [86]	Chewing detection system: in-ear microphone and photoplethysmography sensor	✓	-	-	-	✓	-	-	ML—support vector machine
Papapanagiotou et al., 2021 [87]	Samsung Galaxy ear buds for bite weight estimation	✓	-	-	-	✓	-	-	ML, DL—linear regression, support vector regression, and neural network-based estimators
Sun et al., 2015 [105]	eButton: food image recognition using sensors (cameras, light sensor, a 3-in-1 inertial measurement unit, accelerometer, gyroscope, magnetometer), audio processor, proximity sensor, barometer, GPS receiver	✓	-	-	-	✓	-	-	ML—support vector machines (SVMs), hidden Markov model
Chen et al., 2012. [32]	Nutritional diagnosis expert system (web-based)	-	✓	-	-	-	-	✓	Not specified
Agne et al., 2024 [19]	ChatGPT Food4Me algorithm	-	-	✓	-	-	✓	-	NLP, GPT
Niszczota et al., 2023 [83]	ChatGPT (Version 3.0)	-	-	✓	-	-	✓	-	NLP—LLM
Ponzo et al., 2024 [91]	ChatGPT (Version 3.5)	-	-	✓	-	-	✓	-	NLP—LLM
Qarajeh et al., 2023 [92]	ChatGPT (Version 3.5), ChatGPT (Version 4), Bard AI (Gemini), Bing Chat	-	-	✓	-	-	✓		NLP—LLM
Bohn et al., 2024 [26]	LiFANA dietary support app	-	-	✓	-	-	-	✓	Not specified
Chen et al., 2015 [31]	Diet recommendation system for chronic diseases	-	-	✓	-	-	-	✓	ML—decision tree
Hsu et al., 2011 [53]	Web-based food composition system	-	-	✓	-	-	-	✓	NLP—fuzzy decision model
Karakan et al., 2022 [57]	Al algorithm for personalised nutrition strategy accounting for gut microbiome	-	-	✓	-	-	-	✓	ML
Kobayashi et al., 2024 [62]	Food knowledge graph and recipe recommendation system	-	-	✓	-	-	-	✓	Probabilistic logic programing
Marashi-Hosseini et al., 2023 [76]	Clinical decision-making support system: using fuzzy interference system	-	-	✓	-	-	-	✓	Fuzzy inference system
Papastratis et al., 2024 [88]	ChatGPT and deep generative model	-	-	✓	-	-	-	✓	NLP—LLM, DL
Wang et al., 2023 [113]	Personalized recommendation system for “Carbohydrate-Protein” supplements	-	-	✓	-	-	-	✓	ML, DL—propagation (BP) neural networks, gradient-boosted regression trees (GBRT), enumeration method
Bul et al., 2023 [28]	Web-based diabetes nutrition care platform	-	-	✓	-	-	✓	✓	DL, NLP
Hieronimus et al., 2024 [49]	ChatGPT (Version 3.5), Bard (Gemini)	-	-	✓	-	-	✓	✓	NLP—LLM
Kiriakedis et al., 2024 [60]	ChatGPT (Version 4) by OpenAI	-	-	✓	-	-	✓	✓	NLP—LLM
Amft et al., 2009 [21]	Wearable sensor: microphone, EMG, weight scale	-	-	-	✓	✓	-	-	ML
Cohen et al., 2023 [36]	Contactless drinking and fluid intake detection using Lidar camera	-	-	-	✓	✓	-	-	DL
Hossain et al., 2020 [51]	AIM 2.0 wearable sensor system: flexible bend sensor, camera	-	-	-	✓	✓	-	-	DL—CNN-based image classifier
Ocay et al., 2017 [84]	Nutritrack app: food recognition (mobile app)	-	-	-	✓	✓	-	-	ML
Lee et al., 2023 [69]	Integrated digital healthcare platform using AI-based dietary management (mobile app)	-	-	-	✓	✓	-	✓	Not specified
Holmes et al., 2019 [50]	WeightMonitor: Chatbot	-	-	-	✓	-	✓	-	Not specified
Lázaro et al., 2010 [65]	PERSONA platform: nutritional management and facilitating tool for interventions	-	-	-	✓	-	✓	✓	Not specified
Davis et al., 2020 [37]	Paola: virtual AI health assistant (on Slack communication platform)	-	-	-	✓	-	-	✓	NLP
Lee et al., 2020 [68]	Joint audio–ultrasound food recognition: Doppler sonar at jaw and neck, sensor modules (ultrasonic transmitter) at hyoid bone and jaw	✓	✓	-	-	✓	-	-	DL—DNN
Thames et al., 2021 [106]	Nutrition5k: dataset of 5k diverse computer vision algorithm baseline for incorporating depth sensor data to improve nutrition predictions	✓	✓	-	-	✓	-	-	DL—CNN
Mertes et al., 2020 [78]	Plate system utilising weight sensors and bite algorithm	-	-	✓	✓	✓	-	-	ML—random forest
Vasiloglou et al., 2020 [110]	Medipiatto smartphone app to assess MED diet adherence (mobile app)	-	-	✓	✓	✓	-		DL—CNN
Hsiao et al., 2011 [52]	Diet management system with image recognition, personalised recommendations, and feedback (mobile app)	-	-	✓	✓	✓	-	✓	Not specified
Kwon et al., 2024 [64]	SMART-liver app: ambient-assisted nutrition advisor	-	-	✓	✓	✓	-	✓	Not specified
Nakaoka et al., 2021 [81]	Sensor-equipped chopsticks and nudging system (gateway device and digital art canvas)	-	-	✓	✓	✓	-	✓	DL
Rafferty et al., 2021 [93]	Heali App: AI dietary mobile app enhancing adherence to a low-FODMAP diet	-	-	✓	✓	✓	-	✓	Not specified
Kirk D. et al., 2023 [61]	ChatGPT by OpenAI (Version 3.0)	-	-	✓	✓	-	✓	-	NLP—LLM
Maher et al., 2020 [75]	Paola: AI virtual health coach (via Slack) for Mediterranean-style dietary intervention	-	-	✓	✓	-	✓	-	NLP
Samaan et al., 2023 [95]	ChatGPT (Version 4)	-	-	✓	✓	-	✓	-	NLP—LLM
Beyeler M. et al., 2023 [25]	HealthBot web-based tool	-	-	✓	✓	-	✓	✓	NLP
Buchan et al., 2024 [27]	INA: AI-powered virtual assistant platform	-	-	✓	✓	-	✓	✓	ML
Liao et al., 2024 [71]	ChatGPT (Version 3.5)	-	-	✓	✓	-	✓	✓	NPL—LLM
Abeltino et al., 2022 [18]	Personal metabolic avatar (PMA)	-	-	✓	✓	-	-	✓	DL
Burgermaster et al., 2020 [29]	Patient-generated health data-driven decision-making platform	-	-	✓	✓	-	-	✓	ML
Fernandes et al., 2023 [40]	PRIMO: Prime Implicant Maintenance of Outcome tool for weight management experts (mobile app)	-	-	✓	✓	-	-	✓	ML—random forest
Garcia et al., 2019 [43]	Pan-Cook-Eat: web-based meal planned recommendation app	-	-	✓	✓	-	-	✓	ML—forward chaining algorithm
Gonzalez-Flo et al., 2023 [44]	Evolutionary algorithm to assist with management of T2DM	-	-	✓	✓	-	-	✓	Evolutionary algorithm
Hauptmann et al., 2022 [46]	Nutrilize: mobile app	-	-	✓	✓	-	-	✓	ML—content-based algorithm
Sano et al., 2015 [96]	HealthAware advice platform (desktop app)	-	-	✓	✓	-	-	✓	ML
Tunali et al., 2024 [107]	Enbiosis personalized nutrition model: AI-based personalised low-FODMAP diet	-	-	✓	✓	-	-	✓	ML
Turnin et al., 2021 [108]	Remote monitoring programme including lifestyle education software: Nutri-Kiosk, Acti-Kiosk, and Nutri-Educ	-	-	✓	✓	-	-	✓	Not specified
Jin et al., 2024 [56]	Generative pretrained transformer-based dietary recommendation system for haemodialysis (web platform)	-	-	✓	✓	✓	✓	✓	GPT
Amft et al., 2008 [22]	Wearable sensor: microphone, EMG, body inertial sensors	✓	-	-	✓	✓	-	-	ML
Ben Neriah et al., 2019 [23]	LoseIt! mobile app	✓	-	-	✓	✓	-	-	Not specified
Chin et al., 2019 [34]	Machine learning models and data mapping (comparable to ASA24 reports)	✓	-	-	✓	✓	-	-	ML
Fontana et al., 2012 [42]	Wearable sensor system: jaw motion sensor, self-report push button	✓	-	-	✓	✓	-	-	ML—linear and RBF support vector machine (SVM) classifiers, pattern recognition
Heremans et al., 2020 [47]	Food intake detection via heart-rate variability on ECGs using artificial neural networks	✓	-	-	✓	✓	-	-	ML- ANN
Hezarjaribi et al., 2018 [48]	Speech to nutrient information (S2N1): nutrition monitoring system (mobile app)	✓	-	-	✓	✓	-	-	NLP and text mining
Karmakar et al., 2023 [58]	Multimodal MS-TCN model: recognition of common dietary behaviours: cameras, inertial measurement units	✓	-	-	✓	✓	-	-	DL—CNN
Khan et al., 2022 [59]	Wearable neck band: microphone, radio module	✓	-	-	✓	✓	-	-	ML—random forest classifier
Lee et al., 2017 [67]	FIT-EVE&ADAM wearable sensor system: EMG embedded in armband, food image data, thermal cameras	✓	-	-	✓	✓	-	-	NLP—LLM
Liu et al., 2024 [73]	iEat: wearable sensor system using wrist worn electrodes	✓	-	-	✓	✓	-	-	DL—neural network
Papathanail et al., 2022 [90]	AI-powered system that calculates Mediterranean diet adherence scores (mobile app)	✓	-	-	✓	✓	-	-	DL—CNN
Schiboni et al., 2018 [97]	Wearable camera: Raspberry Pi Zero, a camera attached to cap (privacy preserving feature)	✓	-	-	✓	✓	-	-	DL—DNN
Shao et al., 2021 [100]	Generative adversarial network, layer normalisation (LN), and group normalisation for food image analysis	✓	-	-	✓	✓	-	-	DL—generative adversarial network (GAN)
Krishnakumar et al., 2021 [63]	Wellthy CARE mobile app: e-coaching, decision support system	✓	-	-	✓	-	✓	✓	Not specified
Chew et al., 2024 [33]	eTRIP app to increase awareness on eating habits	✓	-	-	✓	✓	✓	✓	Not specified
Sowah et al., 2020 [103]	Diabetes management system	✓	-	-	✓	✓	✓	✓	ML, DL—tensor flow neural network; k-nearest neighbour (KNN) algorithm
Farooq et al., 2016 [39]	Wearable sensor system: glasses, piezoelectric strain sensor, accelerometer	✓	✓	-	✓	✓	-	-	ML—support vector machine (SVM) classifiers
Fontana et al., 2014 [41]	Automatic ingestion monitor: jaw motion sensor, hand gesture sensor, accelerometer	✓	✓	-	✓	✓	-	-	ML—ANN, pattern recognition
Ji et al., 2020 [55]	Keenoa mobile app	✓	✓	-	✓	✓	-	-	ML
Lin et al., 2020 [72]	WiEat: device-free eating monitoring system (mobile/laptop app)	✓	✓	-	✓	✓	-	-	ML—support vector machine (SVM)
Papathanail et al., 2023 [89]	goFOODTM: automatic system food segmentation, recognition, and nutrient estimation	✓	✓	-	✓	✓	-	-	DL—CNN
Walker et al., 2014 [111]	ID-HMS: wearable sensory system with an external throat microphone system, TMS (microphones, placed over the throat over the laryngeal prominence)	✓	✓	-	✓	✓	-	-	ML
Wang et al., 2024 [112]	Eat-Radar: Wearable sensor systems with radars and cameras to capture gestures in relation to food intake	✓	✓	-	✓	✓	-	-	ML, DL—3D temporal convoluted network
Zhang et al., 2023 [114]	Multi-modality model for processing food image extractions and metabolic readings (e.g., CGM) for calorie prediction	✓	✓		✓	✓	-	-	ML, DL
Oh et al., 2022 [85]	LIBIT app: integrative mHealth program	✓	✓		✓	✓	-	✓	Not specified
Martino et al., 2021 [77]	DoEatWell (m-health app) and decision-making support system: clinical decision-making support tool for malnutrition screening	✓	✓	-	✓	-	-	✓	ML
Nakata et al., 2022 [82]	CALO mama Plus: weight loss coaching mobile app	✓	-	✓	✓	✓	-	✓	Not specified
Ben-Yacov et al., 2023 [24]	Algorithm that provides personalised postprandial-targeting (PPT) diet	✓	-	✓	✓	-	-	✓	ML
Hansel et al., 2017 [45]	ANODE: e-coaching program is a web-based nutritional support tool	✓	-	✓	✓	-	-	✓	Not specified
Sefa-Yeboah et al., 2021 [98]	Mobile- and web-based genetic-algorithm-based platform for obesity management	-	✓	✓	✓	✓	-	✓	GA—genetic algorithm
Silva et al., 2022 [102]	Personalised Dietary Recommender System using user-based and item-based collaborative filtering	-	✓	✓	✓	✓	-	✓	ML
Moyen et al., 2022 [79]	Keenoa (mobile app linked to web platform) compared to ASA24	✓	✓	✓	✓	✓	-	-	ML
Lee et al., 2024 [66]	Nutritional intake model management system	✓	✓	✓	✓	✓	-	✓	DL—CNN
Shoneye et al., 2019 [101]	Technology-assisted dietary assessment or TADA mobile food record app (mobile app)	✓	✓	✓	✓	✓	-	✓	Not specified
De Marchi et al., 2022 [38]	e-health Chatbot (webapp app)	✓	✓	✓	✓	-	✓	-	Not specified
Naja et al., 2024. [80]	ChatGPT (Version 3)	✓	✓	✓	✓	-	✓	✓	NLP
Jactel et al., 2023 [54]	Machine learning-designed personalised elimination diet	✓	✓	✓	✓	-		✓	ML
Salloum et al., 2018 [94]	Personal Intelligent Nutrition (PIN): automates patient health assessment and meal plans	✓	✓	✓	✓	-	-	✓	Fuzzy logic
Shamanna et al., 2020 [99]	Twin Precision Nutrition (TPN) program underpinned by Twin Precision Technology	✓	✓	✓	✓	-	-	✓	ML
Valero-Ramon et al., 2019 [109]	Process mining algorithm to support malnutrition assessment	✓	✓	✓	✓	-	-	✓	ML—process mining
Sun et al., 2023 [104]	Artificial intelligence (AI)-based nutritionist program: ChatGPT and GPT 4.0	✓	✓	✓	✓	✓	✓	✓	NLP—LLM; DL

Abbreviations: Assessment (Ax); diagnosis (Dx); intervention (I); monitoring and evaluation (M&E); Rec. Sys. (recommendation system); machine learning (ML); EMG (electromyography); deep learning (DL); support vector machines (SVMs); natural language processing (NLP); language learning model (LLM); generative pre-trainer (GPT); convoluted neural network (CNN); deep neural network (DNN); genetic algorithm (GA).

**Table 5:** Ethical considerations (privacy and data) (*n* = 11) and safety (nutrition practice) (n = 6) themes explored in studies in the scoping review.

Ethics: Privacy (*n* = 11)	Safety in Nutrition Practice (*n* = 6)
Privacy Preservation: Captured and Accessed Privacy preservation using depth cameras ensures that the surrounding captured individuals are not identifiable [36,83]. Potential in capturing unwanted images of individuals surrounding the wearer [50]. Reduced privacy-infringing content captured [97]. Accounting for the collection of sensitive data, such as GPS location, and invasion of privacy of the device’s wearer and other surrounding individuals should be addressed using automated data processing to reduce human observation of user data [105]. User anonymity Patient/user anonymity is maintained [25]. Transmission and Storage of Data Data security was utilised in 5 systems: section encoding via secure socket layer (SSL) or transport-layer security. Critical information was encoded, user and administrator access were controlled, and personal information was collated with user agreement [85]. Patient data was transmitted daily via a secure cellular network [99]. A secure platform was designed for the monitoring interface linking participants to investigators and for the collection of personal data [108]. Authorisation of Data Authorised users may view biometric information from the individual. Authorisation is sharing-based—individuals may choose to share data with their physician) [111]. Development of Guidelines Focus on patient autonomy, informed consent, and human oversight is an important aspect of AI-supported dietary counselling and medical nutrition therapy [56,92]. Importance of development of ethical guidelines for integrating AI into medical nutrition therapy [56].	Warnings and Disclaimers ChatGPT responses were accompanied with a disclaimer for errors and suggested that individual seek a healthcare professional for more individualised advice [19]. Live dietitian team was available (8 a.m.–8 p.m.) to confirm or adjust selected AI machine guidance when platform does not have an answer or cannot adequately answer. Often for more complex patients with multiple chronic conditions or food intolerances/allergies [27]. Chatbots: Lack of accountability, provision of harmful/incomplete or incorrect advice; potential to fabricate non-existent references and develop nonsensical outputs based on the input/prompt; confident tone of Chatbot can lead to an overestimation of scientific rigor [80]. Human expert oversight Potential to suggest incorrect allergen-free meal ideas. Includes safety information on seeking healthcare professional guidance and allergen information for some products [83]. Nutritional guidelines (i.e., European Food Safety Authority (EFSA)) were used and guided the system towards accuracy and robustness [72]. For the identification of incorrect responses, it is advised that generated responses should be reviewed by a human nutrition expert within 48 h [104].

## Data Availability

The original contributions presented in the study are included in the article/Supplementary Materials; further inquiries can be directed to the corresponding author.

## Supplementary Materials

The following supporting information can be downloaded at https://www.mdpi.com/article/10.3390/nu17223515/s1, Table S1: Title and abstract search for IEEE Database; Table S2: Search terms for the ACM Library; Table S3: Search terms for Scopus; Table S4: Medline (Ovid) Search; Table S5: Embase (Ovid) Search; Table S6: Summary of the characteristics of the included studies (*n* = 97). Studies organised based on the study’s chronic medical condition of focus.
