# Supplementary material for: The Use of Artificial Intelligence (AI) to Support Dietetic Practice Across Primary Care: A Scoping Review of the Literature
Source: Nutrients. 2025 Nov 10;17(22):3515. doi: 10.3390/nu17223515 (PMC12655762; doi:10.3390/nu17223515)
Supplement: Supplementary file 1 [file nutrients-17-03515-s001.zip › nutrients-3921424-supplementary.pdf]

Review

The use of Artificial Intelligence (AI) to support dietetic practice across primary care: A Scoping Review of the Literature

Kaitlyn Ngo [1, 2, +], Simone Mekhail [1, 2,, +], Virginia Chan [1,2], Xinyi Li [1, 2], Annabelle Yin [1,2], Ha Young Choi [1,2], Margaret Allman-Farinelli [1,2], Juliana Chen [1,2,*]

Supplementary Material

Supplementary Material S1. IEEE Database Search

Table S1. Title and abstract search of the IEEE Database.

Title	Abstract
**(((“Title”: Artificial intelligence OR machine learning OR deep learning OR generative ai OR chatgpt OR chatbot OR fuzzy logic OR knowledge base OR neural network OR robotic OR ambient intelligence OR automated reason* OR cognitive technology OR heuristic OR cognitive comput* OR hyperheuristic OR multicriteria decision making OR multi* analys?s OR image recognition OR food recognition OR ingredient recognition OR meal recognition OR image based recognition OR optical character recognition) AND (“Title”: nutrition OR nutrition therap* OR nutrition care process* OR diet* OR dietary NEAR/2 assessment OR nutrition NEAR/2 assessment OR nutrition NEAR/2 monitoring OR dietary NEAR/2 monitoring OR diet therapy OR diet intake OR nutria* intake) AND (“Abstract”: feasibility OR pilot OR trial OR intervention OR human)))<br>Filters applied: Conference, Journals, Early Access Articles, 2024–2024**	((((“Abstract”: Artificial intelligence OR machine learning OR deep learning OR generative ai OR chatgpt OR chatbot OR fuzzy logic OR knowledge base OR neural network OR robotic OR ambient intelligence OR automated reason* OR cognitive technology OR heuristic OR cognitive comput* OR hyperheuristic OR multicriteria decision making OR multi* analys?s OR image recognition OR food recognition OR ingredient recognition OR meal recognition OR image based recognition OR optical character recognition) AND (“Abstract”: nutrition OR nutrition therap* OR nutrition care process* OR diet* OR dietary NEAR/2 assessment OR nutrition NEAR/2 assessment OR nutrition NEAR/2 monitoring OR dietary NEAR/2 monitoring OR diet therapy OR diet intake OR nutria* intake) AND (“Abstract”: feasibility OR pilot OR trial OR intervention OR human))))<br>Filters applied: Conference, Journals, Early Access Articles, 2024–2024

Supplementary Material S2. ACM Library Search

Table S2. Search terms for the ACM Library.

Search Terms
**[[All: “artificial intelligence”] OR [All: “deep learning”] OR [All: “machine learning”] OR [[All: “generat* ai”] AND [All: “chatgpt”]] OR [All: “chatbot”] OR [All: “fuzzy logic”] OR [All: “natural language process*”] OR [All: “automated reason*”] OR [All: “computer heurist*”] OR [All: “cognitive comput*”] OR [All: “cognitive robot*”] OR [All: “robot* process* auto*”] OR [All: “image* recogni*”] OR [All: “food* recogni*”] OR [All: “diet* recogni*”] OR [All: “meal* recogni*”] OR [All: “ingredient recogni*”] OR [All: “artificial intelligence algorithm”] OR [All: “machine learning algorithm”] OR [All: “deep learning algorithm”]] AND [[All: “diet#ian*”] OR [All: “dietetic*”] OR [All: “dietetic practice”] OR [All: “dietetic care”] OR [All: “diet* therap*”] OR [All: “diet* care”] OR [All: “diet* treat*”] OR [All: “nutria* therap*”] OR [All: “nutri* care”] OR [[All: “nutri* treat*”] AND [All: “nutri* intake”]] OR [All: “nutri* intervent*”] OR [All: “nutri* assess*”] OR [All: “nutri* assess*”] OR [All: “nutri* diagnos*”] OR [All: “nutri* monit*”] OR [All: “nutri* evaluat*”] OR [All: “nutri* record*”] OR [All: “nutri* therap*”] OR [All: “diet* intake”] OR [All: “diet* intervent*”] OR [All: “diet* assess*”] OR [All: “diet* assess*”] OR [All: “diet* diagnos*”] OR [All: “diet* monit*”] OR [All: “diet* evaluat*”] OR [All: “diet* record*”] OR [All: “diet* therap*”] OR [All: “food* intake”] OR [All: “food* intervent*”] OR [All: “food* assess*”] OR [All: “food* diagnos*”] OR [All: “food* monit*”] OR [All: “food* evaluat*”] OR [All: “food* record*”] OR [All: “food therap*”]] AND [E-Publication Date: (01 January 2024 TO*)]**

Supplementary Material S3. Scopus Search

Table S3. Search terms for Scopus

Search Terms
**(TITLE-ABS-KEY (“Artificial intelligence” OR “deep learning” OR “machine learning” OR “generat* AI” OR “ChatGPT” OR “chatbot” OR “fuzzy logic” OR “natural language process*” OR “automated reason*” OR “computer heurist*” OR “cognitive comput*” OR “cognitive robot*” OR “robot* process* auto*” OR “((image* or food* or diet* or meal* or ingredient) w/2 recogni*)” OR “((artificial intelligence or machine learning or deep learning) w/4 algorithm)”) AND TITLE-ABS-KEY (“diet#ian*” OR “dietetic*” OR “(dietetic w/3 (practice or care))” OR “((diet* or nutri*) w/3 (therap* or care or treat*))” OR “((nutri* or diet* or food*) w/3 (intake or intervent* or assess* or diagnos* or monit* or evaluat* record* or therap*))”)) AND PUBYEAR > 2006 AND (LIMIT-TO (LANGUAGE, “English”))**

Supplementary Material S4. Medline (Ovid) Search

Table S4. Ovid MEDLINE(R) ALL Search <1946 to 20 August 2024>.

**1**	**Exp Artificial Intelligence/**	**205,462**
**2**	exp telemedicine/	48,847
**3**	(Artificial intelligence or deep learning or machine learning or generat* AI or ChatGPT or chatbot).tw.	190,645
**4**	(fuzzy logic or natural language process* or automated reason* or computer heurist* or cognitive comput* or cognitive robot* or robot* process* auto*).tw.	11,318
**5**	((image* or food* or diet* or meal* or ingredient) adj2 recogni*).tw.	4012
**6**	((artificial intelligence or machine learning or deep learning) adj4 algorithm).tw.	10,984
**7**	1 or 2 or 3 or 4 or 5 or 6	361,940
**8**	exp Nutrition Therapy/	116,923
**9**	exp diet/	342,620
**10**	exp Nutritional Sciences/	20,935
**11**	medi* nutri* therap*.tw.	1001
**12**	(diet#ian* or dietetic*).tw.	9289
**13**	(dietetic adj3 (practice or care)).tw.	494
**14**	((diet* or nutri*) adj3 (therap* or care or treat*)).tw.	60,679
**15**	((nutri* or diet* or food*) adj3 (intake or intervent* or assess* or diagnos* or monit* or evaluat* record* or therap*)).tw.	213,016
**16**	8 or 9 or 10 or 11 or 12 or 13 or 14 or 15	580,562
**17**	7 and 16	2364
**18**	limit 17 to (english language and humans and yr = "2024-Current")	157

Supplementary Material S5. Embase (Ovid) Search

Table S5. Embase Classic + Embase Search <1947 to 20 August 2024>.

**1**	**Exp Artificial Intelligence/**	**113,247**
**2**	exp artificial intelligence software/	1175
**3**	exp artificial intelligence chatbot/	3479
**4**	(Artificial intelligence or deep learning or machine learning or generat* AI or ChatGPT or chatbot).tw.	223,480
**5**	(fuzzy logic or natural language process* or automated reason* or computer heurist* or cognitive comput* or cognitive robot* or robot* process* auto*).tw.	13,842
**6**	((image* or food* or diet* or meal* or ingredient) adj2 recogni*).tw.	4996
**7**	((artificial intelligence or machine learning or deep learning) adj4 algorithm).tw.	15,138
**8**	1 or 2 or 3 or 4 or 5 or 6 or 7	289,768
**9**	exp diet/	478,303
**10**	exp diet therapy/	461,213
**11**	exp dietary intake/	721,940
**12**	exp nutritional assessment/	43,380
**13**	medi* nutri* therap*.tw.	1492
**14**	(diet#ian* or dietetic*).tw.	15,899
**15**	(dietetic adj3 (practice or care)).tw.	798
**16**	((diet* or nutri*) adj3 (therap* or care or treat*)).tw.	85,690
**17**	((nutri* or diet* or food*) adj3 (intake or intervent* or assess* or diagnos* or monit* or evaluat* record* or therap*)).tw.	291,777
**18**	9 or 10 or 11 or 12 or 13 or 14 or 15 or 16 or 17	1,426,663
**19**	8 and 18	4039

Supplementary Material S6. Summary of characteristics of included studies (n = 97)

Supplementary Table S6. Summary of the characteristics of the included studies (n = 97). Studies organised based on the study's chronic medical condition of focus.

**Author (Country)**	**Study Type**	**Population**				**Concept**	**Context**
		Sample Size (N)	Age (years)	Medical Condition of Focus	Dietitian Involvement	Intervention: AI integrated technology	Setting
**Ben-Yacov et al. 2023 [1] (Israel)**	Analytical - RCT	200	50 ± 7	Prediabetes	Dietary guidance during intervention & follow- up	Algorithm designed post prandial-targeting (PPT) diet with real-time feedback via smartphone app	Community
**Bul et al. 2023 [2] (United Kingdom)**	Mixed Methods	73	59.0 ± 11.1	Prediabetes, T1DM, T2DM	NS	Web-based platform providing personalised recipes, menu planning and automated shopping lists	Community
**Burgermaster et al. 2020 [3] (United States of America)**	Mixed Methods - Pilot	12 (inclusive of clinical diabetes educators, dietitians & nurses)	NS	T2DM	Conducted interviews, established a standard for comparison, final evaluation comments	Automated data driven decision making support tool for dietary counselling, personal nutrition goal setting and recommendations	Outpatient clinical
**Gonzalez-Flo et al. 2023 [4] (Italy)**	Descriptive	NS - obtained from dataset of another study	NS	T2DM	NS	Evolutionary algorithm to assist with T2DM management via optimisation of food intake timing, calorie calculations and insulin administration	Community
**Krishnakumar et al. 2021 [5] (India)**	Analytical	102	50.8	T2DM	NS	Wellthy CARE mobile app: e-coaching, decision support system	Community
**Lee et al. 2023 [6] (South Korea)**	Analytical - RCT	294	56.11	T2DM	Sending personalised nutrition intervention messages	Digital integrated health care management platform with image recognition and AI driven dietary management (mobile app)	Community

**Moyen et al. 2022 [7] (Canada)**	Analytical	136	46.1 ± 14.6	T1DM, T2DM	Manual adjustment of incorrect food intake entries into the app by participants	Keenoa (mobile app): entry of food images and calculation of nutrient information for dietary intake monitoring (compared to ASA24)	Community
**Shamanna et al 2020 [8] (India)**	Analytical	64	52.4 ± 10.0	T2DM	NS	Twin Precision Nutrition (TPN) program utilising Twin Precision Technology for daily personalised nutrition guidance	Outpatient clinical
**Sowah et al. 2020 [9] (Ghana)**	Analytical	NS	NS	T1DM, T2DM	Clinical requirements and design analysis of the system was based on discussions with collaborators from the Department of Nutrition and Food Science - the diet type of patients was determined to be an essential approach suitable for the diabetes management system	Diabetes management system: determines the nutrition needs of patients and provide meal recommendations, notification on timing of medication administration, identification of factors that modulate blood glucose levels, physical activity tracking, education and provides report-based feedback to primary care physicians.	Laboratory setting
**Sun et al. 2023 [10] (China)**	Descriptive	206 patients, 26 endocrinology clinical doctors	20 - 60	T2DM	Evaluation of ChatGPT response to professional	Artificial intelligence (AI)–based nutritionist program: uses ChatGPT, GPT- 4.0 and image recognition for meal nutrient calculations and dietary recommendations	Outpatient clinical

					clinical dietitian responses		
**Turnin et al. 2021 [11] (France)**	Analytical - RCT	282	NS	T2DM	NS	Remote telemonitoring program software: Nutri-Kiosk & Nutri – Educ – including self-learning quizzes for assessment of nutrition knowledge, personalised nutrition education and advice	Outpatient clinical
**Zhang et al 2023. [12] (United States of America)**	Descriptive	27 (dataset)	NS	Prediabetes, T2DM	NS	Multi-modality model utilising data from continuous glucose monitoring and food images for food calorie intake estimation	Community
**Abeltino et al. 2022 [13] (Italy)**	Descriptive	4	43.5	Weight Management - Overweight	Development of nutrition plans	Personalised Metabolic Avatar (PMA) deep learning model utilising simulations and evaluations of diet plans and weight variations	Community
**Agne et al. 2024 [14] (Germany)**	Descriptive	20	NS	Weight Management - Obese	NS	ChatGPT (version 3.5) vs Food4Me algorithm for dietary based recommendations	Community
**Ben Neriah et al. 2019 [15] (United States of America)**	Analytical	123787	18-65	Weight management – Overweight, Obese	NS	LoseIt! Mobile app with food image recognition tool for automated dietary intake tracking	Community
**Beyeler M, et al. 2023 [16] (Switzerland)**	Mixed Methods	12	18-69	Weight management – Gastric Bypass	Provided nutrition counselling at bariatric centre appointments	Health Bot (HB) used to provide information-based support and answer patient questions	Outpatient clinical
**Chew et al. 2024 [17] (Singapore)**	Mixed Methods	230	31.25 ± 9.98	Weight management – Overweight	NS	eTRIP app: self-monitoring system to increase awareness of personal eating habits to build self-regulation	Community
**Davis et al. 2020 [18] (Australia)**	Analytical	31	45-75<br>56.2 ± 8	Weight management – Overweight, Obese	Gathered a list of frequently asked questions from	Paola: AI virtual assistant to provide responses to an individual's questions on Slack communication platform	Community

					previous diet and exercise trials		
**Fernandes et al. 2023 [19] (United States of America)**	Mixed Methods	14	45+/- 5	Weight management – Obesity	Evaluated whether explanations produced by the tools were understandable and reliable for use	Prime Implicant Maintenance of Outcome (PRIMO): tool developed to predict weight loss program engagement and success for weight management experts	Outpatient clinic
**Hauptmann et al. 2022 [20] (Germany)**	Mixed Methods	34	33 - 41	Weight management – Overweight	NS	Nutritilize: mobile application with a personalised nutrition assistance system to track dietary intake, recipe recommendations and visual feedback on dietary behaviour features	Community
**Holmes et al. 2019 [21] (United Kingdom)**	Descriptive	15	41-45 (median)	Weight management – Overweight	NS	WeightMentor: chatbot designed as a self-help motivation tool through reliance on self-reporting dietary intake. Hosted on Facebook Messenger.	Community
**Lozano et al. 2023 [22] (United States of America)**	Analytical - Pilot	24	35.0 +/- 9.5	Weight management – Overweight	NS	Openfit: application for automatic identification and quantification of food intake through short video capture for estimated energy intake	Laboratory setting
**Maher et al. 2020 [23] (Australia)**	Analytical	31	45-75<br>56.2 +/- 8.0	Weight management – Overweight, Obese	NS	Paola: virtual health coach to assist in MED Intervention (MediLiPal). Hosted on Slack communication platform.	Community
**Nakata et al. 2022 [24] (Japan)**	Analytical - RCT	141	Intervention: 42.3 ± 9.4<br>Control: 44.0 ± 9.1	Weight management – Overweight, Obese	NS	CALO mama Plus: application that functions a diet program provider (e.g., recording and analysis of diet , physical activity & provision of personalised advice)	Community

**Sefa-Yeboah et al. 2021 [25] (Ghana)**	Analytical	30	20-35 (Sample data provided in Table 7 of article; not complete)	Weight management – Obese	Involved in testing of the program	Genetic algorithm platform: Provides healthy food and meal recommendations for obesity management patients with a focus on energy balance and taking account other medical conditions	Community
**Heremans et al. 2020 [26] (United States of America)**	Analytical	126	18 -65	Dyspepsia	NS	System for food intake detection via analysing of heart rate variations on electrocardiograms using Artificial Neural Networks	Laboratory setting
**Jactel et al. 2023 [27] (United States of America)**	Analytical	39	18–65	IBS, Crohn's Disease, Ulcerative Colitis	Evaluation of nutritional adequacy based on the participant's list of trigger foods.	Digital nutrition tool using a machine learning algorithm to guide a personalised elimination diet with phase-to-phase monitoring	Community
**Karakan et al. 2022 [28] (Turkey)**	Analytical - Pilot	127	Intervention: 47.0 ± 10.0<br>Control: 44.9 ± 9.7	IBS - M	Designing and administrating diet based on AI recommended micronutrient profiles. Monitoring diet adherence.	AI algorithm designed personalised nutrition therapy based on the patient's gut microbiome.	Community
**Rafferty et al. 2021 [29] (Australia)**	Analytical - RCT; Pilot	58	Control: 25.7 +/- 11.9<br>Intervention: 27.2 +/- 9.5	IBS	Co-designed software with software engineers	Heali AI: App provides nutrition information and personalised evidence-based recommendations to improve adherence to a low FODMAP diet. Accounts for when user is at home, grocery shopping or eating out.	Community
**Samaan et al. 2023. [30] (United States of America)**	Analytical	2 (dietitians)	Not Specified	IBD	Evaluation of ChatGPT responses	ChatGPT (version 4) : Chatbot provides easy to understand and conversational responses to user's questions	Outpatient clinical

**Tunali et al. 2024. [31] (Turkey)**	Analytical - RCT	120	18 – 65	IBS	Face to face consultations and delivery of tailored menu plans	Enbiosis personalised nutrition model: AI based personalised Low FODMAP diet and recommendations on based on the patient's gut microbiome.	Outpatient clinical
**Kwon et al. 2024 [32] (South Korea)**	Analytical - RCT	102	48.9 +/-13.74	NAFLD	Consultations with participants on goal setting and evaluation. Provided monthly feedback from dietary intake recorded in app.	SMART-liver: app designed to support self-management of NAFLD via providing dietary education, real-time chat channel messaging and self-monitoring of dietary and lifestyle factors.	Outpatient clinical
**Chen et al 2012. [33] (Taiwan)**	Descriptive	100	NS	Chronic Kidney Disease Haemodialysis (Protein Energy Malnutrition)	Gathering patient information including physical exam findings and diagnoses. Assisted with nutritional diagnosis guidelines to convert into programming rules.	Nutrition diagnosis expert system: a rule-based engine that is used to assist with efficiency and accuracy of nutritional diagnosis of Protein Energy Malnutrition	Outpatient clinical
**Jin et al. 2024 [34] (China)**	Analytical	88	NS	End Stage Renal Disease Haemodialysis	NS	Generative Pre-trained Transformer Based Dietary Recommendation Guidance System: Delivers dietary guidance on potassium levels in foods and personalised dietary recommendations for Haemodialysis patients	Outpatient clinical

**Kiriakedis et al. 2024 [35] (United States of America)**	Descriptive	4 (1 Dietitian)	NS	Nephrolithiasis	Evaluation of ChatGPT's responses for accuracy, completeness and appropriateness	ChatGPT (version 4): Chatbot used to compare laboratory urine collection values to provide dietary recommendations for Nephrolithiasis patients	Outpatient clinical
**Chen et al. 2015 [36] (Taiwan)**	Descriptive	1	70	Hypertension	Provided meal proof standard values	Diet recommendation system: provides user with food and meal recommendations based on their chronic health conditions	Care Facility
**Kobayashi et al. 2024 [37] (Japan)**	Descriptive	NS	NS	Dyslipidaemia, Diabetes	NS	Knowledge graph and meal recommendation system: provides recommended recipes taking into account user's chronic health conditions	Laboratory setting
**Hansel et al. 2017 [38] (France)**	Analytical - RCT	120	18-75	T2DM Obesity	NS	ANODE e-coaching nutrition support tool: designed to improve patient lifestyle habits through education, dietary intake monitoring and advice according to national guidelines	Outpatient clinical
**Oh et al. 2022 [39] (South Korea)**	Analytical- Pilot	37	40 - 70	T2DM Hypertension	NS	LIBIT app: Records dietary intake, exercise and values from self-measuring devices to provide feedback.	Outpatient clinical
**Ponzo et al. 2024 [40] (Italy)**	Descriptive	NS	NS	Dyslipidaemia, Hypertension, T2DM, Obesity, NAFLD, CKD, Sarcopenia	Questions and prompts generated for input were formulated by medical doctors and registered dietitians	ChatGPT (Version 3.5)'s ability to respond to commonly asked health and nutrition questions	Outpatient clinical

**Marashi-Hosseini et al. 2023 [41] (Iran)**	Analytical	100	53.2 ±1.302	Metabolic Syndrome	Commented on results from questionnaire used to establish recommended macronutrient requirements for health condition. Development of gold standard to compare algorithm's output.	Clinical decision-making support system using fuzzy interference model.	Outpatient clinical
**Naja et al. 2024. [42] (United Arab Emirates)**	Descriptive	2 (Dietitians)	NS	Metabolic Syndrome	Evaluation of ChatGPT's responses in accordance to Nutrition Care Manual with focus on accuracy, clarity, coherence and practicality.	ChatGPT (Version 3): Chatbot assessed on dietary management, nutrition care process recommendations and menu planning	Outpatient clinical
**Silva et al. 2022. [43] (Brazil)**	Analytical	15 105	35–74	Metabolic Syndrome	NS	Personalised Dietary Recommender System using user-based, and item based collaborative filtering	Laboratory setting
**Bohn et al. 2024 [44] (Luxembourg)**	Analytical	Portugal: 53 Netherlands: 107	Portugal: 60-81 Netherlands: 52 - 86	Aging population (Within Healthy Weight Range)	NS	LIFANA app: personalised meal recommendation system considering user personal information, taste preferences and macronutrient requirements. Includes GoLive wearable clip.	Community
**Lázaro et al. 2010 [45] (Spain)**	Descriptive	10	55 -75	Aging population (Preventing malnutrition)	NS	Ambient Assisted Nutritional Advisor (AANA) system on PERSONA platform: meal recommender system	Laboratory setting

						that allows for meal nutritional modifications, taste preferences and provides feedback for collected dietary intake habits and adherence	
**Martino et al. 2021 [46] (Italy)**	Analytical	NS	NS	Aging Population (Prediction of malnutrition risk)	NS	DoEatWell (DEW) app + Decision Support System: Utilisation of DEW m-health app for collection of patient data (e.g., nutritional and body composition monitoring) accompanied with AI techniques and models for early detection of malnutrition risk.	Care facility
**Valero-Ramon et al. 2019 [47] (Spain)**	Analytical	96	83.5±12.3	Aging population (Weight changes and Malnutrition screening)	NS	PALIA: process mining algorithm to support decision making in malnutrition assessment	Care Facility
**Buchan et al. 2024 [48] (United States of America)**	Analytical	3310	18-91<br>Median: 57	All cancer types – primarily genitourinary, gynaecologic, gastrointestinal, lung	Development of database for algorithm. Manual review and modification of algorithm responses for more complex cases/questions.	INA: an on-demand virtual assistant expert platform to assist with facilitating nutrition counselling and self-management for cancer patients.	Community
**De Marchi et al. 2022 [49] (Italy)**	Analytical - Pilot	46	57.2	Neurology - Amyotrophic Lateral Sclerosis	Conduced assessment with multidisciplinary team. Designed a personalised, flexible, normo-caloric dietary plans.	eHealth Chatbot: Allows patients to register dietary through a conversation interface with a chatbot.	Community
**Niszczota et al. 2023 [50] (Poland)**	Descriptive	1	30	Food allergies	Generation of prompts and assessment of	ChatGPT (version 3): Chatbot used to generate allergen-specific menu and food recommendations.	Community

					responses by ChatGPT		
**Amft et al. 2007 [51] (Switzerland)**	Descriptive	4	20-30 years	Healthy	NS	Wearable Sensor Technology: Audio recordings of chewing sounds detection of food intake	Laboratory setting
**Amft et al. 2009 [52] (Switzerland)**	Descriptive	8	20 to 35 years	Healthy	NS	Wearable Sensor Technology: Pattern recognition system for audio recordings for recognition of food types and chewing cycles	Laboratory setting
**Amft et al. 2008 [53] (Switzerland)**	Evaluation of Technology	4	25-35	Healthy	NS	Wearable On-body Sensor Technology: continuous recognition of dietary-related activities (e.g., swallowing, arm movements, chewing)	Laboratory setting
**Chen et al. 2023 [54] (Japan)**	Descriptive	4	23-32	Healthy	NS	First Bite/First Chew (wearable sensor technology) - glasses and wristband with microcontroller and inertial measurement units for food intake classification	Laboratory setting
**Chin et al. 2019 [55] (United States of America)**	Descriptive	214, n = 63 used the ASA24-2014 version and n = 151 used the ASA24-2016	NS (Adults)	Healthy	Supervised a team which were estimating lactose values from the ASA24-reported foods & manual look up of foods in NDSR	Several machine learning models and data mapping for nutrient prediction (compared to ASA-24 reports)	Laboratory setting
**Farooq et al. 2016 [56] (Switzerland)**	Descriptive	10	29.03 ± 12.20	Healthy - no medical conditions that would impact chewing	NS	Wearable sensor technology: glasses, piezoelectric strain sensor and accelerometer. Designed for automatic detect food intake in presence of physical motion.	Laboratory setting
**Fontana et al. 2014 [57]**	Descriptive	12	26.7 ± 3.7	Healthy - no medical conditions that	NS	Automatic Ingestion Monitor (AIM) (Wearable sensor technology): integration of jaw and hand motion,	Community

**(United States of America)**				would impact normal food intake		accelerometer sensors. Use of sensor fusion and pattern recognition to detect food intake.	
**Fontana et al. 2012 [58] (United States of America)**	Descriptive	7	18-34	Healthy	NS	Wearable sensor system: piezoelectric film for jaw motion sensor and self-report push button. Pattern recognition algorithm for discriminate between solid food and fluid intake.	Laboratory setting
**Garcia et al. 2019. [59] (Philippines)**	Mixed methods	30 (24 general users, 6 dietitians)	NS	Healthy	Served on a panel of human expert validators	Pan – Cook – Eat: tailored meal plan recommendation app application based on Philippine Dietary Reference Intake values	Community
**Hezarjaribi et al. 2018 [60] (United States of America)**	Analytical	10	18 - 30	Healthy	NS	Speech to Nutrient Information (S2N1) smartphone program: extraction of dietary intake from verbal communication	Laboratory setting
**Hossain et al. 2020. [61] (United States of America)**	Descriptive	5<br>30 participants - first 25 used for training	23.5 ± 4.9	Healthy – no chewing conditions	NS	AIM 2.0 (wearable sensor system): wearable egocentric camera to detect food and no food images in real time	Community
**Hsiao et al. 2011 [62] (Taiwan)**	Descriptive	64	18-22	Healthy	NS	Intelligent Diet Management Service: mobile application with image recognition to capture and update meal content, provides personalised meal plans	Laboratory setting
**Hsu et al. 2011 [63] (Taiwan)**	Analytical	30 (Participants)<br>19 (Dietitians)	20-50	Healthy	Development of database of menus, Retrieval of participant data from dietary records Adjustments of personalised dietary recommendation	Web-based food composition system: includes search function for food composition databases, assists calculate dietary intake, and guidance tool for decision making in nutrition counselling	Laboratory setting

					s & nutrition information. Evaluation process.		
**Ji et al.2020 [64] (Canada)**	Analytical - RCT	72	38.5	Healthy	Evaluation of food images and nutrition analysis. Application of the Dietitians of Canada Handy Guide to Servings Sizes.	Keenoa (mobile app): entry of food images prior to consumption, manual entry of food intake and calculation of total nutrients (compared to 3-day food diary)	Community
**Karmakar et al. 2023 [65](Japan)**	Descriptive	30	NS	Healthy	NS	Multimodal MS-TCN model: recognition of the nine most common dietary intake behaviours and habits	Laboratory setting
**Khan et al. 2022 [66] (Bangladesh)**	Descriptive	20	20-25	Healthy	NS	Wearable Neck Band: microphone, radio module for automated dietary monitoring	Laboratory setting
**Kirk, et al. 2023 [67] (Netherlands)**	Descriptive	18 (dietitians) 9 (nutrition experts)	39	Healthy	Gathered the most asked nutrition questions with corresponding dietitian answers. Graded ChatGPT response for scientific accuracy, applicability and comprehensibilit y.	ChatGPT (version 3.0): assessment of the competency of chat to respond to common nutrition questions	Outpatient clinical
**Lee et al. 2017 [68]**	Analytical	7	NS	Healthy	NS	FIT-EVE&ADAM wearable sensory system: electromyography embedded armband, thermal cameras, food	Laboratory setting

**(United States of America)**						image data to provide feedback on eating cycles	
**Lee et al. 2020 [69] (Korea)**	Analytical	10	22 - 51	Healthy	NS	Joint Audio-Ultrasound Food Recognition: Doppler sonar at jaw & neck, sensor modules (ultrasonic transmitter) at hyoid bone & jaw	Community
**Lin et al. 2020 [70] (United States of America)**	Analytical	20	NS	Healthy	NS	WiEat: device free eating monitoring system (Mobile/Laptop App)	Community
**Liu et al. 2024 [71] (Germany)**	Analytical	10	NS	Healthy	NS	iEat: wearable sensor system using wrist worn electrodes for bioimpedance sensing to detect food intake activities	Community
**Mertes et al. 2020 [72] (China)**	Analytical	24	63 ± 10	Healthy – can self feed	NS	Plate sensing system comprised of weight sensor and bite algorithm to determine the quantity of food consumed per meal.	Outpatient clinical
**Papapanagiotou et al. 2017 [73] (Greece)**	Analytical	14 (dataset)	NS	Healthy	NS	Chewing detection system comprised of in-ear microphone, accelerometer and photoplethysmography sensor to detect chewing	Laboratory setting
**Papapanagiotou et al. 2021 [74] (Greece)**	Analytical	8 (dataset)	25±1.07	Healthy	NS	Bite weight detection and estimation through utilising audio and non-audio features from recordings obtained via using Samsung Galaxy ear buds	Laboratory setting
**Papathanail et al. 2023 [75] (Switzerland)**	Analytical	50	29.2 +/- 11.4	Healthy	Carried out 24-hour dietary recalls on participants.	goodFOOD: smartphone based program for assessing dietary intake via analysing food images for food segmentation, food recognition, food volume estimation, barcode scanning and nutrient estimation	Community
**Salloum et al. 2018 [76] (Lebanon)**	Analytical	16 (real case studies)	33.82	Healthy	Evaluation of participant data and findings.	Personal Intelligent Nutrition (PIN): automates patient health state	Outpatient clinical

		4 (nutrition experts)				assessment and uses it to generate personalised meal plan	
**Sano et al. 2015 [77] (United States of America)**	Analytical	30	<25 - 59	Healthy	NS	HealthAware advice desktop application provides recommendations for stress, sleep, diet and exercise. Paired with FitBit data.	Community
**Schiboni et al. 2018 [78] (Germany)**	Analytical	1	18 - 25	Healthy	NS	Head mounted wearable camera system: RaspberryPiZero, camera attached to cap and portable battery. Privacy consideration with camera angled to exclude irrelevant details from the environment	Community
**Shao et al. 2021 [79] (United States of America)**	Analytical	NS	18 -70	Healthy	Provided the relative energy per food item in each image used. Conducted structured interview with participants for 24-hour dietary recall	Generative Adversarial Network, Layer and Group Normalisation System to estimate of food portion size from single monocular images	Community
**Shoneye et al. 2019 [80] (Australia)**	Analytical - RCT	NS	24 ± 3.3	Healthy	NS	Technology Assisted Dietary Assessment (TADA) mobile app: recording food recall from user and provides personalised feedback	Community
**Sun et al 2015 [81] (United States of America)**	Descriptive	9	NS	Healthy	NS	eButton: circular chest pin sized wearable sensor device to detect food intake. Comprised of two camera, inertial measurement units, audio processes, proximity sensor, barometer and GPS.	Community
**Thames et al. 2021 [82]**	Analytical	16 (non-	NS	Healthy	Provided portion estimations	Nutrition5K: dataset of 5K diverse computer vision algorithm baseline to	Laboratory setting

**(United States of America)**		nutritionists)<br>4 (nutritionist)			which were then compared	improve nutrition predictions by incorporating depth sensor data	
**Walker et al. 2014 [83] (United States of America)**	Analytical	7	NS	Healthy	NS	AID-HMS: wearable sensor technology comprised of external microphones system to record audio of swallowing to identify instances of food ingestion	Laboratory setting
**Wang et al. 2024 [84] (Belgium)**	Analytical	70	NS	Healthy	NS	Eat-Radar: use of radar and camera to capture and recognise eating and drinking fine gestures to assist in automated food intake monitoring	Laboratory setting
**Wang et al 2023 [85] (China)**	Analytical	171	22.78 ± 2.43	Healthy - Endurance Athletes	NS	Personalised Recommendation System for Carbohydrate Protein Supplements for Athletes. Considers demands of an individual's daily life and health status.	Community
**Chotwanvirat et al. 2024 [86] (United Kingdom)**	Descriptive	NS	NS	Other	Common practices of Thai Dietitians were applied to arrange ingredients and prepare ready to eat foods.	iFood app: image-assisted macronutrient content estimation for dietary assessment. Developed by the Institute of Nutrition, Mahidol University.	Community
**Cohen et al. 2023 [87] (Canada)**	Descriptive	9	24±3	Other	Not Specified	Contactless fluid and drinking intake detection using Lidar camera as a sensor	Laboratory setting
**Hieronimus et al. 2024 [88] (Germany)**	Analytical	NS	NS	Other	Data was compared to dietary reference intakes calculated using the USDA DRI calculator used by health professionals	ChatGPT (version 3.5) compared to Bard (Gemini)'s ability to generate dietary meal plans to meet dietary reference intakes	Laboratory setting

**Lee et al. 2024 [89] (Taiwan)**	Analytical – RCT	NS	NS	Other	Not Specified	Nutritional intake model management system: food analysis algorithms, recipe analysis, feedback on nutritional intake and recommendations on optimal intake	Laboratory setting
**Li et al. 2024 [90] (Australia)**	Descriptive	NS	NS	Other	Dietetic students assessed nutrition-related apps for their suitability to be applied into the NCP	Various free and paid nutrition related apps available on Google Play Store and Apple App Store	Community
**Liao et al. 2024 [91] (Taiwan)**	Descriptive)	30 (Dietitians) 20 (college students)	NS	Other	Assessment on quality of ChatGPT's dietary advice	ChatGPT (Version 3.5)'s ability to provide dietary advice	Laboratory setting
**Nakaoka et al. 2021 [92] (Japan)**	Descriptive	1 (Demonstrator)	NS	Other	Not Specified	eat2pic: utensil sensor-based system. Comprised of sensors in chopsticks and interactive painting canvas. Aims to detect and provide feedback on diet quality (healthy foods) and eating pace. Real time feedback provided to user.	Laboratory setting
**Ocay et al. 2017 [93] (Philippines)**	Analytical	NS	NS	Other	NS	NutriTrack: Android app with photo recognition of food function to allow for nutrition estimations to increase user's awareness of nutrient content in foods	Laboratory setting
**Papastratis et al. 2024 [94] (Greece)**	Analytical	3000 virtual user profiles and 1000 real profiles	NS	Other	NS	Deep generative network and ChatGPT to generate and adjust weekly meal plans based on nutrition guidelines for personalised meal plans. Ability to cater for various cuisines.	Laboratory setting

**Papathanail et al. 2022 [95] (Switzerland)**	Analytical	10 non-expert annotators<br>24 users (feasibility study)	NS	Other	Group identified 31 categories of food items	Mobile app with an AI system that calculates MDA scores through using image recognition of food images taken by smartphone prior to meal.	Community
**Qarajeh et al. 2023 [96] (United States of America)**	Descriptive	Not Specified	NS	Other	NS	Four AI models—ChatGPT (version 3.5), ChatGPT (version 4), Bard AI (Gemini), and Bing Chat<br>Assessment on ability to correctly discern micronutrient content in foods	Community
**Vasiloglou et al. 2020 [97] (Switzerland)**	Analytical	Used Oviva platform<br>5 Annotators<br>4 Dietitians	NS	Other	Manual calculation of MDA scores using the scoring system outlined in the paper<br>Creation and correction of the Oviva database	Medipiatto smartphone app to assess adherence to MED. Relies on food image recognition, portion size estimation and users may manually add in food intake	Community

Abbreviations: type 1diabetes mellitus (T1DM); type 2 diabetes mellitus (T2DM); irritable bowel syndrome (IBS); irritable bowel disease (IBD); non-alcoholic fatty liver disease (NAFLD); Chronic Kidney Disease (CKD); Mediterranean diet (MED); Mediterranean diet adherence (MDA); fermentable oligosaccharides, disaccharides, monosaccharides, and polyols (FODMAP) , randomised control trial (RCT), Not Specified (NS)

References

1. Ben-Yacov, O.; Godneva, A.; Rein, M.; Shilo, S.; Lotan-Pompan, M.; Weinberger, A.; Segal, E. Gut microbiome modulates the effects of a personalised postprandial-targeting (PPT) diet on cardiometabolic markers: a diet intervention in pre-diabetes. *Gut* **2023**, *72*, 1486-1496, doi:https://dx.doi.org/10.1136/gutjnl-2022-329201.
2. Bul, K.; Holliday, N.; Bhuiyan, M.R.A.; Clark, C.C.T.; Allen, J.; Wark, P.A. Usability and Preliminary Efficacy of an Artificial Intelligence-Driven Platform Supporting Dietary Management in Diabetes: Mixed Methods Study. *JMIR Hum Factors* **2023**, *10*, doi:10.2196/43959.
3. Burgermaster, M.; Son, J.H.; Davidson, P.G.; Smaldone, A.M.; Kuperman, G.; Feller, D.J.; Burt, K.G.; Levine, M.E.; Albers, D.J.; Weng, C.; et al. A new approach to integrating patient-generated data with expert knowledge for personalized goal setting: A pilot study. *International journal of medical informatics* **2020**, *139*, 104158, doi:https://dx.doi.org/10.1016/j.ijmedinf.2020.104158.
4. Gonzalez-Flo, E.; Kheirabadi, E.; Rodriguez-Caso, C.; Macia, J. Evolutionary algorithm for the optimization of meal intake and insulin administration in patients with type 2 diabetes. *Frontiers in Physiology* **2023**, *14*, 1149698, doi:https://dx.doi.org/10.3389/fphys.2023.1149698.
5. Krishnakumar, A.; Verma, R.; Chawla, R.; Sosale, A.; Saboo, B.; Joshi, S.; Shaikh, M.; Shah, A.; Kolwankar, S.; Mattoo, V. Evaluating glycemic control in patients of South Asian origin with type 2 diabetes using a digital therapeutic platform: Analysis of real-world data. *Journal of Medical Internet Research* **2021**, *23*, e17908, doi:https://dx.doi.org/10.2196/17908.
6. Lee, Y.B.; Kim, G.; Jun, J.E.; Park, H.; Lee, W.J.; Hwang, Y.C.; Kim, J.H. An Integrated Digital Health Care Platform for Diabetes Management With AI-Based Dietary Management: 48-Week Results From a Randomized Controlled Trial. *Diabetes Care* **2023**, *46*, 959-966, doi:https://dx.doi.org/10.2337/dc22-1929.
7. Moyen, A.; Rappaport, A.I.; Fleurent-Gregoire, C.; Tessier, A.-J.; Brazeau, A.-S.; Chevalier, S. Relative Validation of an Artificial Intelligence-Enhanced, Image-Assisted Mobile App for Dietary Assessment in Adults: Randomized Crossover Study. *Journal of medical Internet research* **2022**, *24*, e40449, doi:https://dx.doi.org/10.2196/40449.
8. Shamanna, P.; Saboo, B.; Damodharan, S.; Mohammed, J.; Mohamed, M.; Poon, T.; Kleinman, N.; Thajudeen, M. Reducing HbA1c in Type 2 Diabetes Using Digital Twin Technology-Enabled Precision Nutrition: A Retrospective Analysis. *Diabetes Therapy* **2020**, *11*, 2703-2714, doi:https://dx.doi.org/10.1007/s13300-020-00931-w.
9. Sowah, R.A.; Bampoe-Addo, A.A.; Armoo, S.K.; Saalia, F.K.; Gatsi, F.; Sarkodie-Mensah, B. Design and Development of Diabetes Management System Using Machine Learning. *International Journal of Telemedicine and Applications* **2020**, *2020*, 8870141, doi:https://dx.doi.org/10.1155/2020/8870141.
10. Sun, H.; Zhang, K.; Lan, W.; Gu, Q.; Jiang, G.; Yang, X.; Qin, W.; Han, D. An AI Dietitian for Type 2 Diabetes Mellitus Management Based on Large Language and Image Recognition Models: Preclinical Concept Validation Study. *Journal of medical Internet research* **2023**, *25*, e51300, doi:https://dx.doi.org/10.2196/51300.
11. Turnin, M.C.; Gourdy, P.; Martini, J.; Buisson, J.C.; Chauchard, M.C.; Delaunay, J.; Schirr-Bonnans, S.; Taoui, S.; Poncet, M.F.; Cosma, V.; et al. Impact of a Remote Monitoring Programme Including Lifestyle Education Software in Type 2 Diabetes: Results of the Educ@dom Randomised Multicentre Study. *Diabetes Therapy* **2021**, *12*, 2059-2075, doi:https://dx.doi.org/10.1007/s13300-021-01095-x.
12. Zhang, L.; Huang, S.; Das, A.; Do, E.; Glantz, N.; Bevier, W.; Santiago, R.; Kerr, D.; Gutierrez-Osuna, R.; Mortazavi, B.J. Joint Embedding of Food Photographs and Blood Glucose for Improved Calorie Estimation. In Proceedings of the 2023 IEEE EMBS International Conference on Biomedical and Health Informatics (BHI), 2023; pp. 1-4.
13. Abeltino, A.; Bianchetti, G.; Serantoni, C.; Ardito, C.F.; Malta, D.; De Spirito, M.; Maulucci, G. Personalized Metabolic Avatar: A Data Driven Model of Metabolism for Weight Variation Forecasting and Diet Plan Evaluation. *Nutrients* **2022**, *14*, doi:https://dx.doi.org/10.3390/nu14173520.

14. Agne, I.; Gedrich, K. Personalized dietary recommendations for obese individuals - A comparison of ChatGPT and the Food4Me algorithm. *Clinical Nutrition Open Science* **2024**, *56*, 192-201, doi:https://dx.doi.org/10.1016/j.nutos.2024.06.001.
15. Ben Neriah, D.; Geliebter, A. Weight Loss Following Use of a Smartphone Food Photo Feature: Retrospective Cohort Study. *JMIR mHealth and uHealth* **2019**, *7*, e11917, doi:https://dx.doi.org/10.2196/11917.
16. Beyeler, M.; Légeret, C.; Kiwitz, F.; van der Horst, K. Usability and Overall Perception of a Health Bot for Nutrition-Related Questions for Patients Receiving Bariatric Care: Mixed Methods Study. *JMIR Hum Factors* **2023**, *10*, doi:10.2196/47913.
17. Chew, H.S.J.; Chew, N.W.S.; Loong, S.S.E.; Lim, S.L.; Tam, W.S.W.; Chin, Y.H.; Chao, A.M.; Dimitriadis, G.K.; Gao, Y.; So, J.B.Y.; et al. Effectiveness of an artificial intelligence-assisted app for improving eating behaviors: Mixed methods evaluation. *Journal of Medical Internet Research* **2024**, *26*, doi:https://dx.doi.org/10.2196/46036.
18. Davis, C.R.; Murphy, K.J.; Curtis, R.G.; Maher, C.A. A Process Evaluation Examining the Performance, Adherence, and Acceptability of a Physical Activity and Diet Artificial Intelligence Virtual Health Assistant. *International journal of environmental research and public health* **2020**, *17*, doi:https://dx.doi.org/10.3390/ijerph17239137.
19. Fernandes, G.J.; Choi, A.; Schauer, J.M.; Pfammatter, A.F.; Spring, B.J.; Darwiche, A.; Alshurafa, N.I. An Explainable Artificial Intelligence Software Tool for Weight Management Experts (PRIMO): Mixed Methods Study. *Journal of Medical Internet Research* **2023**, *25*, e42047, doi:https://dx.doi.org/10.2196/42047.
20. Hauptmann, H.; Leipold, N.; Madenach, M.; Wintergerst, M.; Lurz, M.; Groh, G.; Bohm, M.; Gedrich, K.; Krcmar, H. Effects and challenges of using a nutrition assistance system: Results of a long-term mixed-method study. *User Modeling and User-Adapted Interaction* **2022**, *32*, 923-975, doi:https://dx.doi.org/10.1007/s11257-021-09301-y.
21. Holmes, S.; Moorhead, A.; Bond, R.; Zheng, H.; Coates, V.; McTear, M. WeightMentor, bespoke chatbot for weight loss maintenance: Needs assessment & Development. In Proceedings of the 2019 IEEE International Conference on Bioinformatics and Biomedicine (BIBM), 2019; pp. 2845-2851.
22. Lozano, C.P.; Canty, E.N.; Saha, S.; Broyles, S.T.; Beyl, R.A.; Apolzan, J.W.; Martin, C.K. Validity of an Artificial Intelligence-Based Application to Identify Foods and Estimate Energy Intake Among Adults: A Pilot Study. *Current Developments in Nutrition* **2023**, *7*, 102009, doi:https://dx.doi.org/10.1016/j.cdnut.2023.102009.
23. Maher, C.A.; Davis, C.R.; Curtis, R.G.; Short, C.E.; Murphy, K.J. A Physical Activity and Diet Program Delivered by Artificially Intelligent Virtual Health Coach: Proof-of-Concept Study. *JMIR mHealth and uHealth* **2020**, *8*, e17558, doi:https://dx.doi.org/10.2196/17558.
24. Nakata, Y.; Sasai, H.; Gosho, M.; Kobayashi, H.; Shi, Y.; Ohigashi, T.; Mizuno, S.; Murayama, C.; Kobayashi, S.; Sasaki, Y. A Smartphone Healthcare Application, CALO mama Plus, to Promote Weight Loss: A Randomized Controlled Trial. *Nutrients* **2022**, *14*, doi:https://dx.doi.org/10.3390/nu14214608.
25. Sefa-Yeboah, S.M.; Osei Annor, K.; Koomson, V.J.; Saalia, F.K.; Steiner-Asiedu, M.; Mills, G.A. Development of a Mobile Application Platform for Self-Management of Obesity Using Artificial Intelligence Techniques. *Int. J. Telemed. Appl.* **2021**, *2021*, doi:10.1155/2021/6624057.
26. Heremans, E.R.M.; Chen, A.S.; Wang, X.; Cheng, J.; Xu, F.; Martinez, A.E.; Lazaridis, G.; Van Huffel, S.; Chen, J.D.Z. Artificial Neural Network-Based Automatic Detection of Food Intake for Neuromodulation in Treating Obesity and Diabetes. *Obesity surgery* **2020**, *30*, 2547-2557, doi:https://dx.doi.org/10.1007/s11695-020-04511-6.
27. Jactel, S.N.; Olson, J.M.; Wolin, K.Y.; Brown, J.; Pathipati, M.P.; Jagiella, V.J.; Korzenik, J.R. Efficacy of a Digital Personalized Elimination Diet for the Self-Management of Irritable Bowel

Syndrome and Comorbid Irritable Bowel Syndrome and Inflammatory Bowel Disease. *Clinical and translational gastroenterology* **2023**, *14*, e00545, doi:https://dx.doi.org/10.14309/ctg.0000000000000545.
28. Karakan, T.; Gundogdu, A.; Alagozlu, H.; Ekmen, N.; Ozgul, S.; Tunali, V.; Hora, M.; Beyazgul, D.; Nalbantoglu, O.U. Artificial intelligence-based personalized diet: A pilot clinical study for irritable bowel syndrome. *Gut microbes* **2022**, *14*, 2138672, doi:https://dx.doi.org/10.1080/19490976.2022.2138672.
29. Rafferty, A.J.; Hall, R.; Johnston, C.S. A Novel Mobile App (Heali) for Disease Treatment in Participants With Irritable Bowel Syndrome: Randomized Controlled Pilot Trial. *Journal of medical Internet research* **2021**, *23*, e24134, doi:https://dx.doi.org/10.2196/24134.
30. Samaan, J.S.; Issokson, K.; Feldman, E.; Fasulo, C.; Ng, W.H.; Rajeev, N.; Hollander, B.; Yeo, Y.H.; Vasiliauskas, E. Artificial Intelligence and Patient Education: Examining the Accuracy and Reproducibility of Responses to Nutrition Questions Related to Inflammatory Bowel Disease by GPT-4. *medRxiv* **2023**, doi:https://dx.doi.org/10.1101/2023.10.28.23297723.
31. Tunali, V.; Arslan, N.C.; Ermis, B.H.; Hakim, G.D.; Gundogdu, A.; Hora, M.; Nalbantoglu, O.U. A Multicenter Randomized Controlled Trial Of Microbiome-Based Artificial Intelligence-Assisted Personalized Diet Vs Low Fodmap Diet: A Novel Approach for the Management of Irritable Bowel Syndrome. *The American journal of gastroenterology* **2024**, doi:https://dx.doi.org/10.14309/ajg.0000000000002862.
32. Kwon, O.Y.; Lee, M.K.; Lee, H.W.; Kim, H.; Lee, J.S.; Jang, Y. Mobile App-Based Lifestyle Coaching Intervention for Patients With Nonalcoholic Fatty Liver Disease: Randomized Controlled Trial. *Journal of Medical Internet Research* **2024**, *26*, e49839, doi:https://dx.doi.org/10.2196/49839.
33. Chen, Y.; Hsu, C.Y.; Liu, L.; Yang, S. Constructing a nutrition diagnosis expert system. In Proceedings of the Expert Systems with Applications, 2012; pp. 2132-2156.
34. Jin, H.; Lin, Q.; Lu, J.; Hu, C.; Lu, B.; Jiang, N.; Wu, S.; Li, X. Evaluating the Effectiveness of a Generative Pre-trained Transformers-Based Dietary Recommendation System in Managing Potassium Intake for Hemodialysis Patients. *Journal of renal nutrition : the official journal of the Council on Renal Nutrition of the National Kidney Foundation* **2024**, doi:https://dx.doi.org/10.1053/j.jrn.2024.04.001.
35. Kiriakedis, S.; Duty, B.; Chase, T.; Wusirika, R.; Metzler, I. Using ChatGPT-4 to Analyze 24-Hour Urine Results and Generate Custom Dietary Recommendations for Nephrolithiasis. *Journal of Endourology* **2024**, *38*, 719-724, doi:https://dx.doi.org/10.1089/end.2024.0055.
36. Chen, R.C.; Ting, Y.H.; Chen, J.K.; Lo, Y.W. The nutrients of chronic diet recommended based on domain ontology and decision tree. In Proceedings of the 2015 Conference on Technologies and Applications of Artificial Intelligence (TAAI), 2015; pp. 289-295.
37. Kobayashi, A.; Mori, S.; Hashimoto, A.; Katsuragi, T.; Kawamura, T. Functional Food Knowledge Graph-based Recipe Recommendation System Focused on Lifestyle-Related Diseases. *2024 IEEE 18th International Conference on Semantic Computing (ICSC)* **2024**, 261-268, doi:10.1109/ICSC59802.2024.00048.
38. Hansel, B.; Giral, P.; Gambotti, L.; Lafourcade, A.; Peres, G.; Filipecki, C.; Kadouch, D.; Hartemann, A.; Oppert, J.-M.; Bruckert, E.; et al. A Fully Automated Web-Based Program Improves Lifestyle Habits and HbA1c in Patients With Type 2 Diabetes and Abdominal Obesity: Randomized Trial of Patient E-Coaching Nutritional Support (The ANODE Study). *Journal of medical Internet research* **2017**, *19*, e360, doi:https://dx.doi.org/10.2196/jmir.7947.
39. Oh, S.W.; Kim, K.-K.; Kim, S.S.; Park, S.K.; Park, S. Effect of an Integrative Mobile Health Intervention in Patients With Hypertension and Diabetes: Crossover Study. *JMIR mHealth and uHealth* **2022**, *10*, e27192, doi:https://dx.doi.org/10.2196/27192.
40. Ponzo, V.; Goitre, I.; Favaro, E.; Merlo, F.D.; Mancino, M.V.; Riso, S.; Bo, S. Is ChatGPT an Effective Tool for Providing Dietary Advice? *Nutrients* **2024**, *16*, doi:https://dx.doi.org/10.3390/nu16040469.

41. Marashi-Hosseini, L.; Jafarirad, S.; Hadianfard, A.M. A fuzzy based dietary clinical decision support system for patients with multiple chronic conditions (MCCs). *Scientific reports* **2023**, *13*, 12166, doi:https://dx.doi.org/10.1038/s41598-023-39371-4.
42. Naja, F.; Taktouk, M.; Matbouli, D.; Khaleel, S.; Maher, A.; Uzun, B.; Alameddine, M.; Nasreddine, L. Artificial intelligence chatbots for the nutrition management of diabetes and the metabolic syndrome. *European Journal of Clinical Nutrition* **2024**, doi:10.1038/s41430-024-01476-y.
43. Silva, V.C.; Gorgulho, B.; Marchioni, D.M.; Alvim, S.M.; Giatti, L.; de Araujo, T.A.; Alonso, A.C.; Santos, I.D.S.; Lotufo, P.A.; Bensenor, I.M. Recommender System Based on Collaborative Filtering for Personalized Dietary Advice: A Cross-Sectional Analysis of the ELSA-Brasil Study. *International Journal of Environmental Research and Public Health* **2022**, *19*, 14934, doi:https://dx.doi.org/10.3390/ijerph192214934.
44. Bohn, T.; Ferrini, K.; Stahl, C. LIFANA - toward developing a meal recommender system as a dietary support app for the elderly. *International journal for vitamin and nutrition research. Internationale Zeitschrift fur Vitamin- und Ernahrungsforschung. Journal international de vitaminologie et de nutrition* **2024**, *94*, 221-238, doi:https://dx.doi.org/10.1024/0300-9831/a000795.
45. Lázaro, J.P.; Fides, A.; Navarro, A.; Guillén, S. Ambient Assisted Nutritional Advisor for elderly people living at home. In Proceedings of the Annual International Conference of the IEEE Engineering in Medicine and Biology Society, 2010; pp. 198-203.
46. Martino, F.D.; Delmastro, F.; Dolciotti, C. Malnutrition Risk Assessment in Frail Older Adults using m-Health and Machine Learning. In Proceedings of the ICC 2021 - IEEE International Conference on Communications, 2021; pp. 1-6.
47. Valero-Ramon, Z.; Fernandez-Llatas, C.; Martinez-Millana, A.; Traver, V. A Dynamic Behavioral Approach to Nutritional Assessment using Process Mining. In Proceedings of the 2019 IEEE 32nd International Symposium on Computer-Based Medical Systems (CBMS), 2019; pp. 398-404.
48. Buchan, M.L.; Goel, K.; Schneider, C.K.; Steullet, V.; Bratton, S.; Basch, E. National Implementation of an Artificial Intelligence-Based Virtual Dietitian for Patients With Cancer. *JCO clinical cancer informatics* **2024**, *8*, e2400085, doi:https://dx.doi.org/10.1200/CCI.24.00085.
49. De Marchi, F.; Serioli, M.; Collo, A.; Belotti, E.G.; Alloatti, F.; Biroli, G.; Bolioli, A.; Cantello, R.; Riso, S.; Mazzini, L. A Telehealth Intervention for Nutritional Counseling in Amyotrophic Lateral Sclerosis Patients. *Journal of Clinical Medicine* **2022**, *11*, 4286, doi:https://dx.doi.org/10.3390/jcm11154286.
50. Niszczota, P.; Rybicka, I. The credibility of dietary advice formulated by ChatGPT: Robo-diets for people with food allergies. *Nutrition (Burbank, Los Angeles County, Calif.)* **2023**, *112*, 112076, doi:https://dx.doi.org/10.1016/j.nut.2023.112076.
51. Amft, O.; Kusserow, M.; Troster, G. Automatic Identification of Temporal Sequences in Chewing Sounds. In Proceedings of the 2007 IEEE International Conference on Bioinformatics and Biomedicine (BIBM 2007), 2007; pp. 194-201.
52. Amft, O.; Kusserow, M.; Troster, G. Bite Weight Prediction From Acoustic Recognition of Chewing. *IEEE Transactions on Biomedical Engineering* **2009**, *56*, 1663-1672, doi:10.1109/TBME.2009.2015873.
53. Amft, O.; Troster, G. Recognition of dietary activity events using on-body sensors. *Artificial intelligence in medicine* **2008**, *42*, 121-136, doi:https://dx.doi.org/10.1016/j.artmed.2007.11.007.
54. Chen, J.; Wang, X.; Li, J.; Chernyshov, G.; Huang, Y.; Kunze, K.; Huang, J.; Starner, T.; Zhang, Q. First Bite/Chew: distinguish different types of food by first biting/chewing and the corresponding hand movement. In Proceedings of the CHI EA '23: Extended Abstracts of the 2023 CHI Conference on Human Factors in Computing Systems, <conf-loc>, <city>Hamburg</city>, <country>Germany</country>, </conf-loc>, 2023; p. Article 138.
55. Chin, E.L.; Simmons, G.; Bouzid, Y.Y.; Kan, A.; Burnett, D.J.; Tagkopoulos, I.; Lemay, D.G. Nutrient Estimation from 24-Hour Food Recalls Using Machine Learning and Database Mapping: A Case Study with Lactose. *Nutrients* **2019**, *11*, doi:https://dx.doi.org/10.3390/nu11123045.

56. Farooq, M.; Sazonov, E. A Novel Wearable Device for Food Intake and Physical Activity Recognition. *Sensors (Basel, Switzerland)* **2016**, *16*, doi:https://dx.doi.org/10.3390/s16071067.
57. Fontana, J.M.; Farooq, M.; Sazonov, E. Automatic ingestion monitor: a novel wearable device for monitoring of ingestive behavior. *IEEE transactions on bio-medical engineering* **2014**, *61*, 1772-1779, doi:https://dx.doi.org/10.1109/TBME.2014.2306773.
58. Fontana, J.M.; Sazonov, E.S. A robust classification scheme for detection of food intake through non-invasive monitoring of chewing. In Proceedings of the Annual International Conference of the IEEE Engineering in Medicine and Biology Society. , 2012; pp. 4891-4894.
59. Garcia, M.B. Plan-Cook-Eat: A Meal Planner App with Optimal Macronutrient Distribution of Calories Based on Personal Total Daily Energy Expenditure. In Proceedings of the 2019 IEEE 11th International Conference on Humanoid, Nanotechnology, Information Technology, Communication and Control, Environment, and Management (HNICEM), 2019; pp. 1-5.
60. Hezarjaribi, N.; Mazrouee, S.; Ghasemzadeh, H. Speech2Health: A Mobile Framework for Monitoring Dietary Composition From Spoken Data. *IEEE journal of biomedical and health informatics* **2018**, *22*, 252-264, doi:https://dx.doi.org/10.1109/JBHI.2017.2709333.
61. Hossain, D.; Imtiaz, M.H.; Ghosh, T.; Bhaskar, V.; Sazonov, E. Real-Time Food Intake Monitoring Using Wearable Egocentric Camera. In Proceedings of the 2020 42nd Annual International Conference of the IEEE Engineering in Medicine & Biology Society (EMBC), 2020; pp. 4191-4195.
62. Hsiao, M.; Yeh, Y.F.; Hsueh, P.Y.; Lee, S. Intelligent Nutrition Service for Personalized Dietary Guidelines and Lifestyle Intervention. In Proceedings of the 2011 International Joint Conference on Service Sciences, 2011; pp. 11-16.
63. Hsu, C.-Y.; Huang, L.-C.; Chen, T.M.; Chen, L.-F.; Chao, J.C.J. A web-based decision support system for dietary analysis and recommendations. *Telemedicine and e-Health* **2011**, *17*, 68-75, doi:https://dx.doi.org/10.1089/tmj.2010.0104.
64. Ji, Y.; Plourde, H.; Bouzo, V.; Kilgour, R.D.; Cohen, T.R. Validity and usability of a smartphone image-based dietary assessment app compared to 3-day food diaries in assessing dietary intake among Canadian adults: Randomized controlled trial. *JMIR mHealth and uHealth* **2020**, *8*, doi:https://dx.doi.org/10.2196/16953.
65. Karmakar, A.; Nishida, M.; Nishimura, M. Eating and Drinking Behavior Recognition Using Multimodal Fusion. In Proceedings of the 2023 IEEE 12th Global Conference on Consumer Electronics (GCCE), 2023; pp. 210-213.
66. Khan, M.T.; Ghaffarzadegan, S.; Feng, Z.; Hasan, T. A Fabric-based Inexpensive Wearable Neckband for Accurate and Reliable Dietary Activity Monitoring. In Proceedings of the 2022 25th International Conference on Computer and Information Technology (ICCIT), 2022; pp. 994-997.
67. Kirk, D.; Van Eijnatten, E.; Camps, G. Comparison of Answers between ChatGPT and Human Dieticians to Common Nutrition Questions. *J. Nutr. Metab.* **2023**, *2023*, doi:10.1155/2023/5548684.
68. Lee, J.; Paudyal, P.; Banerjee, A.; Gupta, S.K.S. FIT-EVE&ADAM: Estimation of Velocity & Energy for Automated Diet Activity Monitoring. In Proceedings of the 16th IEEE International Conference on Machine Learning and Applications, 2017; pp. 1071-1074.
69. Lee, K.-S. Joint Audio-Ultrasound Food Recognition for Noisy Environments. *IEEE journal of biomedical and health informatics* **2020**, *24*, 1477-1489, doi:https://dx.doi.org/10.1109/JBHI.2019.2938627.
70. Lin, Z.; Xie, Y.; Guo, X.; Ren, Y.; Chen, Y.; Wang, C. WiEat: Fine-grained Device-free Eating Monitoring Leveraging Wi-Fi Signals. In Proceedings of the 2020 29th International Conference on Computer Communications and Networks (ICCCN), 2020; pp. 1-9.
71. Liu, M.; Zhou, B.; Rey, V.F.; Bian, S.; Lukowicz, P. iEat: automatic wearable dietary monitoring with bio-impedance sensing. *Scientific reports* **2024**, *14*, 17873, doi:https://dx.doi.org/10.1038/s41598-024-67765-5.
72. Mertes, G.; Ding, L.; Chen, W.; Hallez, H.; Jia, J.; Vanrumste, B. Measuring and Localizing Individual Bites Using a Sensor Augmented Plate During Unrestricted Eating for the Aging

Population. *IEEE journal of biomedical and health informatics* **2020**, *24*, 1509-1518, doi:https://dx.doi.org/10.1109/JBHI.2019.2932011.
73. Papapanagiotou, V.; Diou, C.; Zhou, L.; van den Boer, J.; Mars, M.; Delopoulos, A. A Novel Chewing Detection System Based on PPG, Audio, and Accelerometry. *IEEE journal of biomedical and health informatics* **2017**, *21*, 607-618, doi:https://dx.doi.org/10.1109/JBHI.2016.2625271.
74. Papapanagiotou, V.; Ganotakis, S.; Delopoulos, A. Bite-Weight Estimation Using Commercial Ear Buds. In Proceedings of the Annual International Conference of the IEEE Engineering in Medicine and Biology Society., 2021; pp. 7182-7185.
75. Papathanail, I.; Abdur Rahman, L.; Brigato, L.; Bez, N.S.; Vasiloglou, M.F.; van der Horst, K.; Mougiakakou, S. The Nutritional Content of Meal Images in Free-Living Conditions-Automatic Assessment with goFOODTM. *Nutrients* **2023**, *15*, doi:https://dx.doi.org/10.3390/nu15173835.
76. Salloum, G.; Semaan, E.; Tekli, J. PIN Prototype for Intelligent Nutrition Assessment and Meal Planning. In Proceedings of the 2018 IEEE International Conference on Cognitive Computing (ICCC), 2018; pp. 110-113.
77. Sano, A.; Johns, P.; Czerwinski, M. HealthAware: An advice system for stress, sleep, diet and exercise. In Proceedings of the 2015 International Conference on Affective Computing and Intelligent Interaction (ACII), 2015; pp. 546-552.
78. Schiboni, G.; Wasner, F.; Amft, O. A Privacy-Preserving Wearable Camera Setup for Dietary Event Spotting in Free-Living. In Proceedings of the 2018 IEEE International Conference on Pervasive Computing and Communications Workshops (PerCom Workshops), 2018; pp. 872-877.
79. Shao, Z.; Fang, S.; Mao, R.; He, J.; Wright, J.L.; Kerr, D.A.; Boushey, C.J.; Zhu, F. Towards Learning Food Portion From Monocular Images With Cross-Domain Feature Adaptation. In Proceedings of the 2021 IEEE 23rd International Workshop on Multimedia Signal Processing (MMSP), 2021; pp. 1-6.
80. Shoneye, C.L.; Dhaliwal, S.S.; Pollard, C.M.; Boushey, C.J.; Delp, E.J.; Harray, A.J.; Howat, P.A.; Hutchesson, M.J.; Rollo, M.E.; Zhu, F.; et al. Image-Based Dietary Assessment and Tailored Feedback Using Mobile Technology: Mediating Behavior Change in Young Adults. *Nutrients* **2019**, *11*, doi:https://dx.doi.org/10.3390/nu11020435.
81. Sun, M.; Burke, L.E.; Baranowski, T.; Fernstrom, J.D.; Zhang, H.; Chen, H.-C.; Bai, Y.; Li, Y.; Li, C.; Yue, Y.; et al. An exploratory study on a chest-worn computer for evaluation of diet, physical activity and lifestyle. *Journal of healthcare engineering* **2015**, *6*, 1-22, doi:https://dx.doi.org/10.1260/2040-2295.6.1.1.
82. Thames, Q.; Karpur, A.; Norris, W.; Xia, F.; Panait, L.; Weyand, T.; Sim, J. Nutrition5k: Towards Automatic Nutritional Understanding of Generic Food. In Proceedings of the 2021 IEEE/CVF Conference on Computer Vision and Pattern Recognition (CVPR), 2021; pp. 8899-8907.
83. Walker, W.P.; Bhatia, D.K. Automated Ingestion Detection for a Health Monitoring System. *IEEE Journal of Biomedical and Health Informatics* **2014**, *18*, 682-692, doi:10.1109/JBHI.2013.2279193.
84. Wang, C.; Kumar, T.S.; Raedt, W.D.; Camps, G.; Hallez, H.; Vanrumste, B. Eat-Radar: Continuous Fine-Grained Intake Gesture Detection Using FMCW Radar and 3D Temporal Convolutional Network With Attention. *IEEE Journal of Biomedical and Health Informatics* **2024**, *28*, 1000-1011, doi:10.1109/JBHI.2023.3339703.
85. Wang, X.; Li, Z.; Wu, H. Personalized Recommendation Method of “Carbohydrate-Protein” Supplement Based on Machine Learning and Enumeration Method. *IEEE Access* **2023**, *11*, 100573-100586, doi:10.1109/ACCESS.2023.3314699.
86. Chotwanvirat, P.; Prachansuwan, A.; Sridonpai, P.; Kriengsinyos, W. Automated Artificial Intelligence-Based Thai Food Dietary Assessment System: Development and Validation. *Current Developments in Nutrition* **2024**, *8*, 102154, doi:https://dx.doi.org/10.1016/j.cdnut.2024.102154.
87. Cohen, R.; Fernie, G.; Fekr, A.R. Contactless Drink Intake Monitoring Using Depth Data. *IEEE Access* **2023**, *11*, 12218-12225, doi:10.1109/ACCESS.2023.3241835.

88. Hieronimus, B.; Hammann, S.; Podszun, M.C. Can the AI tools ChatGPT and Bard generate energy, macro- and micro-nutrient sufficient meal plans for different dietary patterns? *Nutrition research (New York, N.Y.)* **2024**, *128*, 105-114, doi:https://dx.doi.org/10.1016/j.nutres.2024.07.002.
89. Lee, H.-A.; Liu, C.-Y.; Hsu, C.-Y. Precision Nutrition Management in Continuous Care: Leveraging AI for User-Reported Dietary Data Analysis. *Studies in health technology and informatics* **2024**, *315*, 256-261, doi:https://dx.doi.org/10.3233/SHTI240148.
90. Li, X.; Yin, A.; Choi, H.Y.; Chan, V.; Allman-Farinelli, M.; Chen, J. Evaluating the Quality and Comparative Validity of Manual Food Logging and Artificial Intelligence-Enabled Food Image Recognition in Apps for Nutrition Care. *Nutrients* **2024**, *16*, doi:10.3390/nu16152573.
91. Liao, L.-L.; Chang, L.-C.; Lai, I.J. Assessing the Quality of ChatGPT's Dietary Advice for College Students from Dietitians' Perspectives. *Nutrients* **2024**, *16*, doi:https://dx.doi.org/10.3390/nu16121939.
92. Nakaoka, R.; Nakamura, Y.; Matsuda, Y.; Misaki, S.; Yasumoto, K. eat2pic: Food-tech Design as a Healthy Nudge with Smart Chopsticks and Canvas. In Proceedings of the 2021 IEEE International Conference on Pervasive Computing and Communications Workshops and other Affiliated Events (PerCom Workshops), 2021; pp. 389-391.
93. Ocay, A.B.; Fernandez, J.M.; Palaoag, T.D. NutriTrack: Android-based food recognition app for nutrition awareness. In Proceedings of the 2017 3rd IEEE International Conference on Computer and Communications (ICCC), 2017; pp. 2099-2104.
94. Papastratis, I.; Konstantinidis, D.; Daras, P.; Dimitropoulos, K. AI nutrition recommendation using a deep generative model and ChatGPT. *Scientific Reports* **2024**, *14*, 14620, doi:10.1038/s41598-024-65438-x.
95. Papathanail, I.; Vasiloglou, M.F.; Stathopoulou, T.; Ghosh, A.; Baumann, M.; Faeh, D.; Mougiakakou, S. A feasibility study to assess Mediterranean Diet adherence using an AI-powered system. *Scientific reports* **2022**, *12*, 17008, doi:https://dx.doi.org/10.1038/s41598-022-21421-y.
96. Qarajeh, A.; Tangpanithandee, S.; Thongprayoon, C.; Suppadungsuk, S.; Krisanapan, P.; Aiumtrakul, N.; Garcia Valencia, O.A.; Miao, J.; Qureshi, F.; Cheungpasitporn, W. AI-Powered Renal Diet Support: Performance of ChatGPT, Bard AI, and Bing Chat. *Clinics and Practice* **2023**, *13*, 1160-1172, doi:https://dx.doi.org/10.3390/clinpract13050104.
97. Vasiloglou, M.F.; Lu, Y.; Stathopoulou, T.; Papathanail, I.; Fah, D.; Ghosh, A.; Baumann, M.; Mougiakakou, S. Assessing Mediterranean Diet Adherence with the Smartphone: The Medipiatto Project. *Nutrients* **2020**, *12*, doi:https://dx.doi.org/10.3390/nu12123763.
